# Stability of operational taxonomic units: an important but neglected property for analyzing microbial diversity

**DOI:** 10.1186/s40168-015-0081-x

**Published:** 2015-05-20

**Authors:** Yan He, J Gregory Caporaso, Xiao-Tao Jiang, Hua-Fang Sheng, Susan M Huse, Jai Ram Rideout, Robert C Edgar, Evguenia Kopylova, William A Walters, Rob Knight, Hong-Wei Zhou

**Affiliations:** State Key Laboratory of Organ Failure Research, Department of Environmental Health, Guangdong Provincial Key Laboratory of Tropical Disease Research, School of Public Health and Tropical Medicine, Southern Medical University, Guangzhou, 510515 China; Department of Biological Sciences, Northern Arizona University, PO Box 5640, Flagstaff, AZ 86011-5640 USA; Center for Microbial Genetics and Genomics, Northern Arizona University, PO Box 4073, Flagstaff, AZ 86011-4073 USA; Department of Pathology and Laboratory Science, Warren Alpert Medical School, Brown University, 70 Ship Street, Providence, RI 02912 USA; ., Tiburon, CA 94920 USA; Department of Pediatrics, University of California at San Diego, 9500 Gilman Drive MC0763, La Jolla, CA 92093-0763 USA; Department of Molecular Biology and Genetics, Cornell University, 526 Campus Road, Ithaca, NY 14853 USA; Department of Computer Science and Engineering, University of California at San Diego, 9500 Gilman Drive MC0763, La Jolla, CA 92093-0763 USA

## Abstract

**Background:**

The operational taxonomic unit (OTU) is widely used in microbial ecology. Reproducibility in microbial ecology research depends on the reliability of OTU-based 16S ribosomal subunit RNA (rRNA) analyses.

**Results:**

Here, we report that many hierarchical and greedy clustering methods produce unstable OTUs, with membership that depends on the number of sequences clustered. If OTUs are regenerated with additional sequences or samples, sequences originally assigned to a given OTU can be split into different OTUs. Alternatively, sequences assigned to different OTUs can be merged into a single OTU. This OTU instability affects alpha-diversity analyses such as rarefaction curves, beta-diversity analyses such as distance-based ordination (for example, Principal Coordinate Analysis (PCoA)), and the identification of differentially represented OTUs. Our results show that the proportion of unstable OTUs varies for different clustering methods. We found that the closed-reference method is the only one that produces completely stable OTUs, with the caveat that sequences that do not match a pre-existing reference sequence collection are discarded.

**Conclusions:**

As a compromise to the factors listed above, we propose using an open-reference method to enhance OTU stability. This type of method clusters sequences against a database and includes unmatched sequences by clustering them via a relatively stable *de novo* clustering method. OTU stability is an important consideration when analyzing microbial diversity and is a feature that should be taken into account during the development of novel OTU clustering methods.

**Electronic supplementary material:**

The online version of this article (doi:10.1186/s40168-015-0081-x) contains supplementary material, which is available to authorized users.

## Background

Rapid advances in DNA sequencing technologies over the past decade have allowed us to study communities of microorganisms in far greater depth than was previously possible. Many of these studies involve PCR amplification and sequencing of marker genes (often the 16S small ribosomal subunit RNA (rRNA)) from complex communities of organisms, which can then be compared to databases of known sequences to identify the taxa present in the microbial community. These methods have led to the discovery of new organisms at a much faster rate than taxonomists can describe and name. To facilitate taxonomy-independent analyses and to reduce the computational resources necessary for such, marker gene sequence reads are typically clustered based on sequence similarity, under the assumption that sequences with greater similarity represent more phylogenetically similar organisms. These clusters, or operational taxonomic units (OTUs), are widely used as an analytical unit in microbial ecology studies [1].

Due to the lack of a gold standard of ‘correct’ OTUs, several measurements have been used to evaluate the performance of clustering methods, for example, rationality of OTU structure [2,3], computational efficiency (that is, runtime and memory requirements) [4], and the ability to cope with OTU inflation [5]. However, OTU stability has rarely been studied to date, despite the importance of this property. Here, we define the stability of an OTU by whether it contains the same clustered sequence(s) regardless of the number of sequences that are clustered. If OTUs are found to be unstable when clustering different numbers of sequences in different clustering runs, the sequences in a given OTU may be assigned to different OTUs. Alternatively, sequences assigned to different OTUs can be assigned to a single OTU.

**Figure 1:**
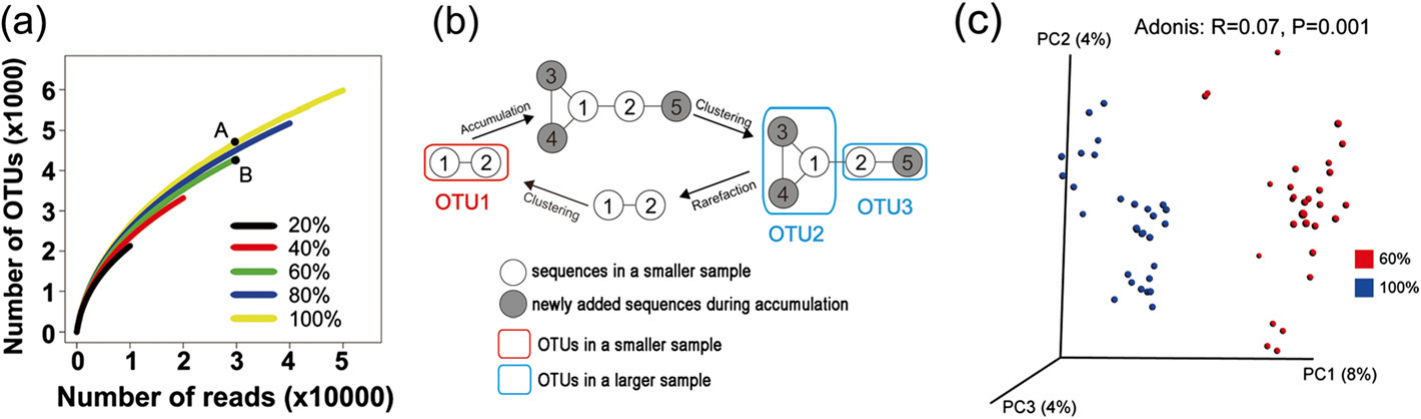
Rarefaction curves, principles underlying unstable complete linkage (CL) clustering, and PCoA based on the Bray-Curtis distance. **(a)** Rarefaction curves generated with CL clustering at five different depths. Point A is the number of OTUs at 30,000 sequences from the 100% dataset, and point B is the number of OTUs at 30,000 sequences from the 60% dataset. **(b)** Principles underlying unstable CL clustering at two sampling depths. White circles indicate individual sequences that were included in both the small and the large subsamples, and dark circles indicate sequences that were added only in the large subsample. Lines indicate pairs of sequences with distances equal to or smaller than the threshold, which could therefore be linked into a single OTU. Large circles in red or blue indicate OTUs in the small and the large subsamples, respectively. **(c)** PCoA based on the Bray-Curtis distance, comparing 60% subsamples with the full datasets using CL. All of the subsamples were rarefied to 30,000 sequences per sample to be included in this analysis.

Roesch *et al.* [6] reported the above detailed clustering artifact soon after next-generation sequencing was applied to 16S rRNA. Using six different sequence subset sizes (ranging from 10,000 to 53,632 sequences) from a single Canadian soil dataset, they showed that larger input sequence counts produced steeper rarefaction curves (Figure 1a). Rarefaction curves plot the alpha-diversity (for example, the number of species or OTUs) found within a given number of observations (DNA sequences). Rarefaction curves are widely used to test whether an environment has been sufficiently sequenced to observe all taxa and to extrapolate the total diversity of the sampled community [1,3]. A rarefaction curve where the slope changes when calculated from a different number of initial sequences directly conflicts with the expected behavior of such a curve and challenges the fundamental principle that the diversity of a whole community can be estimated from a sequenced sample.

In this study, we reveal that unstable OTUs lead to non-overlapping rarefaction curves. We further show that these unstable OTUs can also affect beta-diversity analyses. We also evaluated existing *de novo* and reference-based clustering methods to show that all *de novo* clustering methods are unstable to some extent, while closed-reference clustering generates stable OTUs. Closed-reference clustering has the considerable limitation of excluding any OTUs that are not defined in a pre-existing reference set, which in turn excludes novel OTUs from analysis. As a compromise between generating OTU instability and the potential elimination of novel taxa, we recommend using open-reference OTU clustering [7], which we show to result in more stable OTUs than fully *de novo* clustering methods.

## Results and discussion

### Changing membership of OTUs at different sequencing depths (OTU instability) - a neglected but important property for analyses of microbial diversity

To illustrate the problem created by unstable OTUs, we reproduced the non-overlapping rarefaction curves using the same dataset (Canada soil dataset) and the same clustering method (complete linkage clustering, referred to as CL clustering) employed by Roesch *et al.* (Figure 1a). We randomly subsampled the raw sequences at four sequencing depths (20%, 40%, 60%, and 80% of the input sequences) using 30 replicates of each. We then used complete linkage (CL) clustering to cluster each of the subsamples (definitions of all clustering methods can be found in Additional file 1) and generated rarefaction curves for each sampling depth. In the case of CL clustering, the rarefaction curve produced by a larger subsample is steeper than that produced by a smaller subsample.

One goal when generating rarefaction curves is to support interpolation, meaning that if we create a rarefaction curve from a full dataset, we would like to use that curve to determine how many species would be observed for some number of sequences that amounts to less than the total. For example, when we interpolate from the rarefaction curve created from a full dataset, we estimate that we have approximately 4,500 species if we randomly select 30,000 sequences from the full dataset (point A in Figure 1a). The problem that non-overlapping rarefaction curves pose for interpolation, however, is that if we instead randomly subsampled 30,000 sequences from an 80% subsample of the full dataset, we would estimate that only 4,200 species are represented by these 30,000 sequences (point B in Figure 1a). This scenario would essentially be true in cases where only a few sequences were collected per sample, a phenomenon that conflicts with the expected behavior of rarefaction curves.

We have observed that the non-overlapping of rarefaction curves, as illustrated in Figure 1a, is actually caused by the instability of OTU clustering methods. In other words, the cluster that a sequence is assigned to can be affected by the number of sequences being clustered. An illustration of this hypothesis is shown in Figure 1b. If we observe only two sequences, S1 and S2, within the similarity threshold (indicated by linking with a bar), they are clustered into a single OTU (OTU1). We then add three more sequences, S3, S4, and S5, which could be linked to S1 or to S2, but several pairwise distances exceed the threshold (these pairs are not linked by bars). By definition of CL, pairwise distances for all sequences assigned to a single OTU must fit within the distance threshold [8,9], which could allow S1 and S2 to be separated into OTU2 and OTU3. OTU1 disappears at this sequencing depth, and its sequences are reassigned to two different OTUs, illustrating the problem of OTU instability. Theoretically, adding more sequences tends to split existing OTUs when using the CL algorithm. As a result, when being clustered with a larger dataset versus a smaller dataset, the same sequences will be grouped into more OTUs. This will result in a steepening of the rarefaction curve that is derived from the larger sample and the conclusion that it has a higher alpha-diversity. Rarefaction curves that arise from CL are therefore more sensitive to sequencing depth. Though this effect is weak, it still partially illustrates why, in some cases, the collecting of a number of sequences that is based on a smaller sample size would be expected to produce a rarefaction curve that reaches a plateau, and instead a continually increasing rarefaction curve is produced. This phenomenon of an individual being assigned to different OTUs simply because of increased or decreased sampling depth is obviously problematic. An analogous situation based on traditional (macro-scale) ecology would be if counting different numbers of birds within a fixed area led to the redefinition of which individual birds group together as a species. However, the above-described instability is not due to the occasional identification of novel species, as might be the case in traditional ecology. In contrast, these changes to OTU membership occur systematically within a large proportion of the sequences being reassigned between OTUs.

To further investigate the effect of unstable OTUs on biological interpretation, we next explored beta-diversity using ordination. Using Principal Coordinate Analysis (PCoA), we compared the microbial communities against the full dataset using subsamples comprising 60% of the full dataset. We repeated this subsampling 30 times to create replicates. We then used CL clustering to cluster all of the subsamples, as well as the full dataset, and combined the clustering results by representative OTU sequence (defined as the most abundant sequence in each OTU). The samples were then randomly rarefied to include 30,000 sequences per sample, including the 30 replicate rarefactions that resulted from the clustering of the full dataset. Following rarefaction, all samples contained the same number of sequences so that the only differences among them were the number of sequences that were initially clustered. PCoA demonstrated that these samples separated according to the number of sequences that were initially clustered, indicating that OTU instability results in the same samples appearing to have different compositions (Figure 1c). A similar result was observed when comparing the 20%, 40%, and 80% subsamples against the full dataset (Additional file 2: Figure S1). Furthermore, 125 OTUs (after false discovery rate (FDR) correction) and 26 OTUs (after Bonferroni correction) were determined to be significantly different between these two groups using the Mann-Whitney *U* test. We also tested the effect that unstable OTUs have on calculating taxonomic composition and found the effect to be very limited (Additional file 3: Figure S2 and Additional file 4). This is because these OTUs are still assigned to the same taxa as a consequence of their phylogenetic proximity, despite the fact that they are changing when more sequences are added using CL (also discussed below in the section detailing the tolerance of PCoA to using phylogenetic metrics with unstable OTUs).

### Alternative hierarchical and greedy clustering methods also produce unstable OTUs

**Figure 2:**
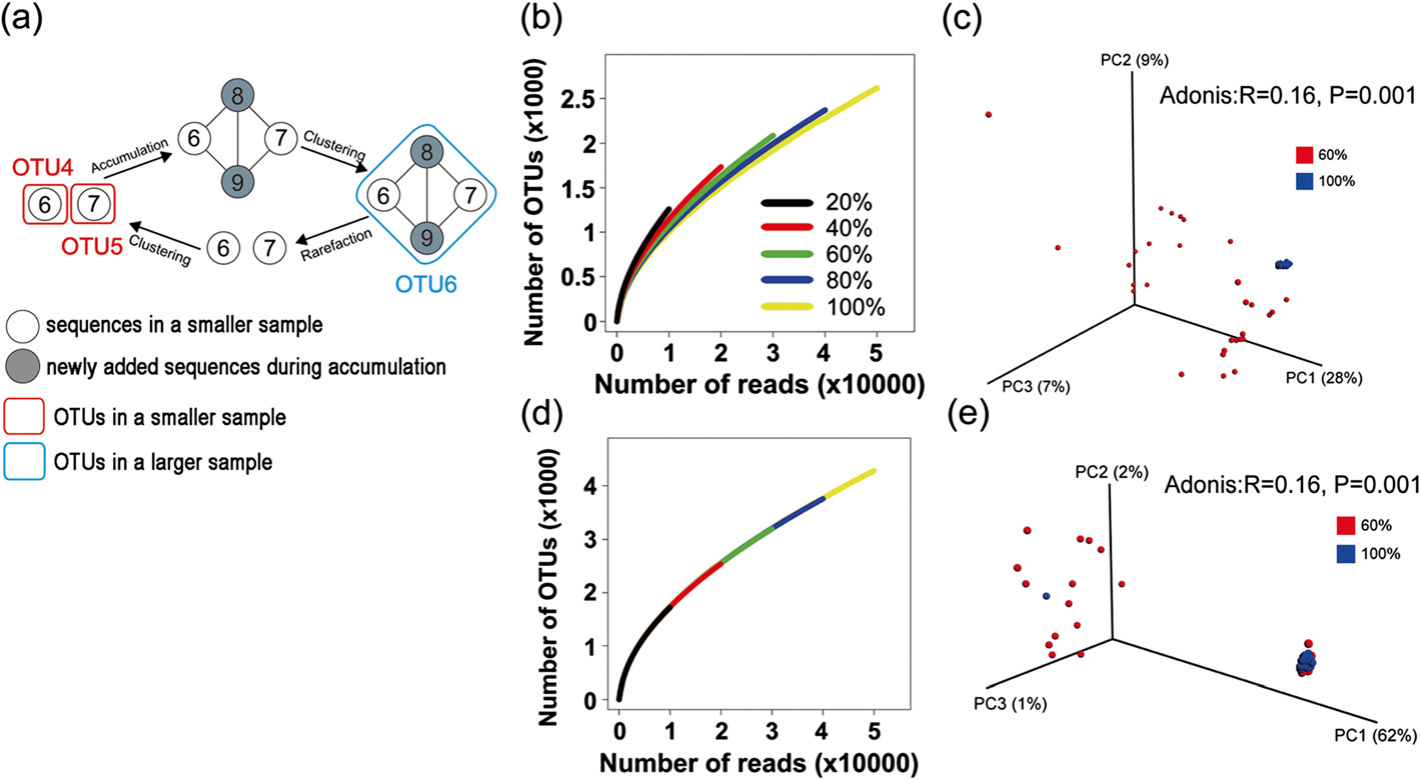
Principles underlying unstable single linkage (SL) clustering, rarefaction curves, and PCoA based on the Bray-Curtis distance. **(a)** Principles underlying unstable SL clustering at two sampling depths. White circles indicate individual sequences that were included in both the small and the large subsamples, and dark circles indicate sequences that are added only in the large subsample. Lines indicate pairs of sequences with distances equal to or smaller than the threshold, which could therefore be linked into a single OTU. Large circles in red or blue indicate OTUs in the small and the large subsamples, respectively. **(b, d)** Rarefaction curves generated with SL **(b)** and average linkage (AL) **(d)** clustering at five different depths. **(c, e)** PCoA based on the Bray-Curtis distance, comparing 60% subsamples with the full datasets using SL **(c)** and AL **(e)**. All of the subsamples were rarefied to 30,000 sequences per sample to be included in this analysis.

All hierarchical methods that are used to determine OTU membership are based on pairwise distances between the sequences in OTUs. CL clustering requires the pairwise distance between all sequences in one OTU to fit within the distance threshold. Single linkage (SL) clustering requires the pairwise distance between any pair of sequences in one OTU to fit within the distance threshold. Average linkage (AL) clustering requires the average pairwise distances between all sequences in one OTU to fit within the distance threshold. As would be expected when using SL clustering (Figure 2a), OTUs tend to be merged when more sequences are added, which is the opposite of the splitting problem that is observed with CL. Accordingly, rarefaction curves created using SL become less steep as subsample size increases (Figure 2b). Beta-diversity is also affected by unstable SL clustering of OTUs (Figure 2c). For example, 167 OTUs (after FDR correction) and 36 OTUs (after Bonferroni correction) were determined to be differentially represented across both the 60% subsample and the full dataset.

The instability produced by average linkage is more complicated because both OTU splitting and OTU merging can occur. These conflicting effects lead to more subtle differences in OTU counts, and the resultant rarefaction curves that are created with AL overlap at different depths (Figure 2d). Furthermore, the AL OTUs themselves are unstable (Additional file 5: Figure S3) due to the large number of OTU splitting and merging events that occur. Additionally, even though these unstable OTUs affect beta-diversity (Adonis, *R* = 0.16, *P* = 0.001), the major separation in PCoA appears to be caused by factors other than sample size; for example, the possible inclusion of differences that result from the input order of the sequences or the presence or absence of certain key sequences within different subsamples (Figure 2e). This observation may result from the sensitivity of AL to the order of input sequences, which would result in different clustering patterns. When using AL, 804 OTUs (after FDR correction) and 5 OTUs (after Bonferroni correction) were differentially represented across the two sampling depths.

Greedy clustering, such as that which is implemented in USEARCH, is another commonly used *de novo* clustering method that is more computationally efficient than CL, SL, and AL. When using greedy clustering, a sequence must be within the distance threshold of a single OTU centroid to be clustered in that OTU. Furthermore, sequences are processed in a defined order, and each query sequence will either be assigned to an existing OTU or as the centroid of a new OTU. If one query sequence is within the distance threshold of multiple existing OTU centroids, it can be assigned to either the closest centroid (here referred to as distance-based greedy clustering (DGC)) or the most abundant centroid (here referred to as abundance-based greedy clustering (AGC)) (Additional file 1). Alternative approaches exist for breaking such ties; however, we chose to limit our focus to those that are the most commonly employed. In the present study, we evaluate USEARCH as a method for greedy clustering (we did not evaluate UPARSE because its clustering algorithm is the same as that used in USEARCH).

**Figure 3:**
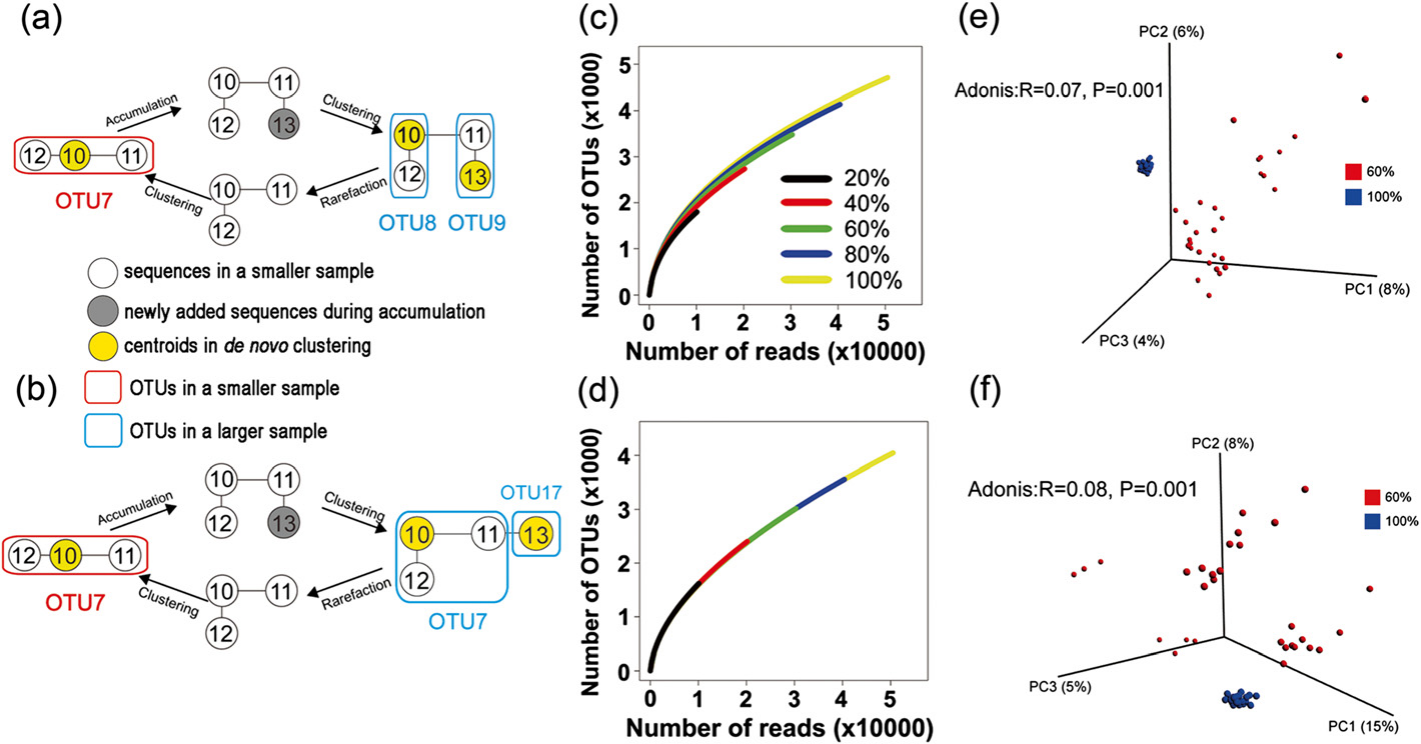
Principles underlying unstable distance-based greedy clustering (DGC) and abundance-based greedy clustering (AGC), rarefaction curves, and PCoA based on the Bray-Curtis distance. **(a, b)** Principles underlying unstable DGC (a) and AGC (b) at two sampling depths. White circles indicate individual sequences that were included in both the small and the large subsamples, and dark circles indicate sequences that were added only in the large subsample. Yellow dots indicate OTU centroids. Lines indicate pairs of sequences with distances equal to or smaller than the threshold, which could therefore be linked into a single OTU. Large circles in red or blue indicate OTUs in the small and the large subsamples, respectively. **(c, d)** Rarefaction curves generated with DGC **(c)** and AGC **(d)** at five different depths. **(e, f)** PCoA based on the Bray-Curtis distance, comparing 60% subsamples with the full datasets using AGC **(e)** and DGC **(f)**. All of the subsamples were rarefied to 30,000 sequences per sample to be included in this analysis.

OTU instability is also a problem in greedy clustering methods and arises from several sources. First, the choosing of centroids is highly dependent on the order in which sequences are processed. Therefore, when the size of a sample is changed, the order of sequences may also be changed. Second, when using DGC, even if the choice of centroids remains stable when the size of the sample is increased, the added sequences can become new centroids and attract members from existing OTUs (this generally will not happen in AGC). For example, imagine that S10, S11, and S12 form OTU7 with S10 as the centroid (Figure 3a,b). If in a subsequent sequencing run another sequence, S13, is added, the processing order of the larger sample may become S10, S13, S11, and S12. In this case, S10 will still be a centroid, but S13 will also become a centroid. S13 then recruits S11, as the distance between the two is smaller than the distance between S11 and S10. In DGC, S11 will end up clustering with S13 rather than S10, and the original OTU7 will be split into OTU8 and OTU9 (Figure 3a). In AGC, S11 will still cluster with S10 and the original OTU7 will retain its original structure (Figure 3b).

We used greedy clustering on alpha rarefaction curves and beta-diversity PCoA to analyze the effects generated by unstable OTUs. As stated above, DGC and AGC both suffer from centroid changeability (this effect is not biased towards OTU splitting or merging), and DGC additionally suffers from the splitting of existing OTUs. As a result, DGC and CL clustering produced similar curves, which became steeper as subsample size increased (Figure 3c). In contrast, AGC produced overlapped curves that were unaffected by depth (Figure 3d). However, as with AL clustering, this does not mean that the OTUs were stable, but only that similar numbers of (possibly different) OTUs were obtained at the different subsampling depths. The unstable OTUs produced in DGC and AGC effect estimations of beta-diversity (Figure 3e,f). In the case of AGC, 392 OTUs (after FDR correction) and 14 OTUs (after Bonferroni correction) were determined to be differentially represented across the two depths, and in the case of DGC, these numbers were 370 and 15, respectively.

**Figure 4:**
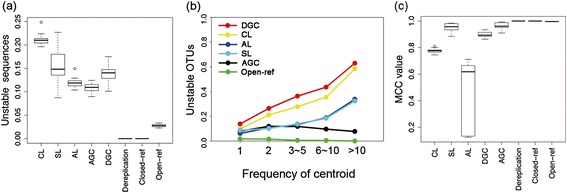
Proportion of unstable sequences, proportion of unstable OTUs, and MCC value of each method. **(a)** Proportion of unstable sequences as created by method. Unstable sequences are defined as sequences that are clustered to one centroid in the 60% subsample but clustered to a different centroid in the 100% (full) dataset. **(b)** Proportion of unstable OTUs as created by method and by frequency of cluster centroids (the values for closed-reference and dereplication are zero and are thus not included in this figure). If an OTU was identical in the 60% and 100% datasets (not including sequences that are not present in the 60% subsample), it is defined as stable. **(c)** MCC value of each method. Higher values correspond to greater stability.

To quantify the differences between these unstable methods, we compared the proportion of unstable sequences and unstable OTUs (Figure 4a,b; Additional file 6: Table S1). CL produced the highest proportion of unstable sequences (approximately 22%), while AL (13%) and AGC (12%) performed slightly better than SL (15%) and DGC (14%). These results were not always consistent when comparing the use of alternative datasets (Additional file 7: Figure S6); however, AGC generally demonstrated the best performance versus the other *de novo* methods. For unstable OTUs, CL and DGC produced the highest proportion of unstable OTUs: approximately 60% of OTUs with centroids with frequencies greater than or equal to 10 were observed to be unstable in each of the methods (>90% were found to be unstable when analyzing certain datasets, as shown in Additional file 7: Figure S6). AL and SL are more stable than either CL or DGC but still resulted in greater than 30% OTU instability for centroids being observed at least 10 times. AGC was found to be the most stable *de novo* method, especially for OTUs with highly abundant centroids.

One *de novo* clustering method that does produce stable OTUs is dereplication or the clustering of sequences that are identical and of equal length (Additional file 8: Figure S4a). As with closed-reference OTU clustering, all OTUs remain absolutely stable across different sequencing depths because clustering is not affected by the composition of the sequence collection being clustered. As a result, rarefaction curves produced using dereplication are overlapping across different depths (Additional file 8: Figure S4b), and beta-diversity is not affected by the size of the subsamples (Additional file 8: Figure S4c). Moreover, not a single OTU is determined to be significantly different between the two groups. It is important to note that dereplication is highly vulnerable to identifying spurious OTUs that result from sequencing error. Due to its stability in binning OTUs, it also produces overlapping rarefaction curves across different depths, indicating that unstable OTUs (rather than sequencing errors) are the main cause of non-overlapping rarefaction curves. Furthermore, the stability of the dereplication method suggests that a higher similarity threshold for clustering may reduce the occurrence of unstable OTUs, as *de novo* clustering methods become more similar to dereplication as the similarity threshold increases. In practice, dereplication clustering yields high numbers of OTUs, which is computationally expensive to employ downstream. Thus, modern dataset sizes prevent us from working with sequences that have only been dereplicated. It is possible that future methods may use approaches based on dereplication to manage the problem of OTU instability. Another extreme example would be the clustering of all sequences into one OTU while that OTU remains absolutely stable. Nevertheless, unlike dereplication, OTUs can be utilized in further analyses, such as alpha-diversity, beta-diversity, and taxonomic composition. Furthermore, clustering all sequences into one OTU can hardly be called ‘clustering’ and is completely useless for downstream analysis.

### Reference-based methods minimize the problem of unstable OTUs

**Figure 5:**
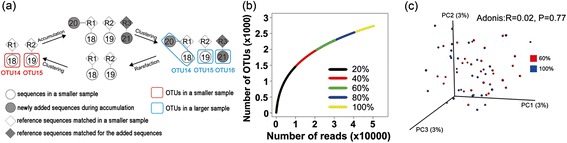
Principles underlying stable closed-reference clustering, rarefaction curves, and PCoA based on the Bray-Curtis distance. **(a)** Principles underlying stable closed-reference clustering at two sampling depths. White circles indicate individual sequences that were included in both the small and the large subsamples, and dark circles indicate sequences that were added only in the large subsample. Diamonds indicate reference sequences. Lines indicate pairs of sequences with distances equal to or smaller than the threshold, which could therefore be linked into a single OTU. Large circles in red or blue indicate OTUs in the small and the large subsamples, respectively. **(b)** Rarefaction curves generated with closed-reference clustering at five different depths. **(c)** PCoA based on the Bray-Curtis distance, comparing 60% subsamples with the full datasets using closed reference clustering. All of the subsamples were rarefied to 30,000 sequences per sample to be included in this analysis.

One feature that all unstable clustering methods have in common is that cluster definitions are dependent upon the input sequences. Closed-reference OTU clustering avoids this dependence with one major practical limitation: during closed-reference OTU clustering, reads are clustered against a reference dataset (for example, the Greengenes database [9]) of pre-calculated centroids and no new centroids are created during clustering, which results in perfectly stable OTUs (Figure 5a). As a result, alpha- and beta-diversity estimations based on closed-reference clustering are not affected by the size of samples (Figure 5b,c), and no OTUs are determined to be significantly different between the two depths. In addition to producing stable OTUs, closed-reference clustering provides several other convenient features. First, the names of the reference sequences can be used as universal OTU identifiers rather than using arbitrarily assigned names, thus facilitating the direct comparison of OTUs across studies. Second, sequence reads from different marker gene regions can be clustered together if the reference dataset consists of full-length marker genes. Finally, closed-reference clustering can parallelize OTU clustering for large datasets. The major limitation of closed-reference OTU clustering is that reads that are outside the similarity threshold to any reference centroids are discarded, such that only the OTUs that are already represented in the database can be ‘observed.’ In processing the Canada soil dataset, approximately 14% of the sequences could not be matched to the reference sequences and were therefore discarded after clustering. This limitation of closed-reference OTU clustering may become trivial as projected improvements are made to reference datasets, leading the corresponding references needed for specific research projects (for example, the gut microbiome) to become more highly developed.

To overcome the limitations of closed-reference OTU clustering, open-reference OTU clustering can be used. Open-reference clustering begins in the same way as closed-reference clustering but continues to cluster the sequences that do not match the reference collection in a *de novo* manner. Although existing *de novo* clustering methods produce unstable OTUs, open-reference clustering can be much more stable than such methods because many sequences are initially clustered by the closed-reference approach. We evaluated OTU stability in open-reference clustering using AGC for the *de novo* clustering step (Figure 4a,b,c) and found it to be a much more effective method than using *de novo* methods alone. The majority of the unstable OTUs were low abundance sequences with no reference match (a category of sequences that is commonly considered to be error-prone). Open-reference OTU clustering produces overlapping rarefaction curves (Additional file 9: Figure S5a), and even though the instability of open-reference OTU clustering still affects PCoA analysis (Additional file 9: Figure S5b), the PC and *R* value (by ADONIS, *R* = 0.03) is lower than with any other *de novo* method alone, as is the number of OTUs that are differentially represented across the two groups (104 OTUs after FDR correction and 2 OTUs after Bonferroni correction). We compared open-reference clustering methods with other *de novo* methods on additional datasets, focusing on the proportion of unstable sequences and unstable OTUs and found that these results are generally consistent across environment types and sequencing technologies (Additional file 7: Figure S6).

In addition to quantifying the instability of OTUs, we used the MCC index to investigate how the clustering of sequence pairs changed based on clustering of the full dataset versus the 60% subset (Figure 4b, Additional file 6: Table S2). It is clear that the two reference-based methods and dereplication clustering have the highest stability by this metric and that AGC is the most stable of the *de novo* clustering methods (Kruskal-Wallis test, *P* < 0.05). AL had the lowest MCC value, indicating that the clustering of many sequences pairs changed when using this method. Alternatively, SL produced a higher MCC value than most of the *de novo* methods, including AL and CL. Nevertheless, part of the reason for the high MCC value of SL is that its FP value equals 0 (sequences that are separated in a smaller subsample will be merged into a single OTU in a larger subsample, but the reverse situation does not happen at all). Thus, due to its severe problems with OTU merging, SL should not be considered a much more stable method.

### Phylogenetic beta-diversity metrics minimize the effect of OTU instability

Unlike non-phylogenetic metrics, where all OTUs are considered equally dissimilar from each other, phylogenetic metrics such as UniFrac take into account the phylogenetic relationship between OTUs when calculating distances between samples. Unstable OTU clustering methods will move sequences between OTUs that would usually be closely related evolutionarily so that the calculated distance between samples should generally remain more similar than it would when using non-phylogenetic diversity metrics. We re-analyzed the effect of unstable OTUs on beta-diversity using CL, SL, AL, AGC, and DGC based on UniFrac distance (Additional file 10: Figure S7). The results show that unstable OTUs of CL, AGC, and DGC minimally affect beta-diversity using UniFrac distance, confirming the hypothesis that when sequences are changing between closely related OTUs with these unstable methods, phylogenetic metrics are more tolerant to that instability. Nevertheless, in SL clustering, distantly related OTUs can ultimately be joined into a single OTU, so that beta-diversity can be affected even when using UniFrac distance. In AL, the major separation is still caused by different clustering patterns, as with the non-phylogenetic metrics.

## Conclusions

Assigning an organism to a different species based on the number of individuals included in a given census is obviously problematic in traditional macro-ecology. Unfortunately, due to an artifact of many OTU clustering methods, the equivalent situation is common in microbial ecology studies that use taxonomic-independent methods. This study demonstrated for the first time that the problem of depth-dependent rarefaction curves that is inherent in most of the *de novo* clustering algorithms arises from unstable OTUs and not from sequencing errors. Furthermore, unstable OTUs may also contribute to depth-dependent beta-diversity estimates and OTU membership. Our results demonstrate that the closed-reference OTU clustering method provides stable OTUs, while all other *de novo* clustering methods (except dereplication) produce unstable OTUs. Unfortunately, the closed-reference OTU method is limited by the availability of databases of known marker gene sequences, which excludes novel OTUs from analysis. To balance this caveat of closed-reference OTU clustering against the problem of OTU instability, we recommend using open-reference OTU clustering employing AGC (for example, as implemented using uclust [4] in QIIME [7]), a relatively stable *de novo* method, to cluster sequences that did not map to the reference database. This allows for clustering of all sequences and introduces minimal OTU instability compared to *de novo* OTU clustering methods (which also cluster all sequences). As new OTU clustering algorithms continue to be rapidly developed, we suggest that OTU stability should be an important consideration in future endeavors. However, we do not argue that OTU stability is the most important quality of clustering methods. Other attributes of clustering methods, such as diminishing sequencing errors, rational OTU structure, niche consistency, and time efficiency, should also be considered when choosing a method for a specific type of analysis or evaluating a novel approach to clustering. Furthermore, as the 16S rRNA gene database is expanding, closed-reference OTU clustering and new algorithms that bypass OTU clustering altogether during microbiome analysis may render problems concerning OTU instability obsolete.

## Methods

The ‘Canada soil’ dataset [GenBank:EF308591-EF361836] was originally used to describe that rarefaction curves generated from the same dataset but at different depths often do not overlap [6] and is used here to demonstrate unstable OTUs. This dataset was one of the earliest reported 454 datasets, and quality information was not available for the sequences contained therein. Although we demonstrated in the results section (via the dereplication method) that OTU instability is introduced by clustering algorithms and not by sequencing errors, we nevertheless removed sequences that contained ambiguous bases (N), potential chimeras identified by UCHIME using the *de novo* mode (using --minchunk 20 --xn 7 –noskipgaps 2) [10], and sequences that were of a length that ranged outside of two standard deviations from the mean. Such sequences were removed for quality control purposes (in the absence of quality scores). The original dataset contained a total of 53,246 sequences, and 13,469 unique sequences remained after dereplication. Following our quality control measures, a total of 50,542 sequences with 13,293 unique tags remained, ranging in length from 86 to 120 bp.

The hierarchical clustering algorithms, CL, AL, and SL, were run using MOTHUR 1.27 [11]. The same pairwise Needleman-Wunsch distance matrix (with default parameters) was calculated for each pair of unique sequences and used in the three clustering algorithms at a 97% identity threshold. We used the QIIME 1.7.0 [12] USEARCH 6.1 wrapper [4] for the AGC and DGC greedy clustering algorithms. We used QIIME 1.7.0 [12] with the Greengenes 13_8 database for closed- and open-reference clustering. These clustering methods are further defined in Additional file 1, along with commands used the execute them.

To investigate the effect of sequencing depths on rarefaction curves, we subsampled our input data (prior to OTU clustering) at 5 depths (20%, 40%, 60%, 80%, and 100% of the total data), with 30 replicates at each depth. We then clustered each of these datasets with each of the clustering methods and created rarefaction curves. The presented data included the average rarefaction curve across all replicates for each clustering method at each depth.

To demonstrate the effect of sequencing depth on beta-diversity, we subsampled our input data to include 60% of sequences prior to OTU clustering and compared it with the full dataset (the first round of subsampling was to create a 60% subsample to compare with the full dataset). Because *de novo* clustering methods do not provide universal OTU identifiers, after clustering each of these datasets, we combined the clustering results according to a representative sequence of each OTU (which we chose to be the most abundant sequence in each OTU). In this way, OTUs with the same representative sequences were merged into a single OTU. We then created a BIOM file [13] from the merged OTUs for further analyses. We computed beta-diversity using QIIME 1.7.0 with all samples rarefied to 30,000 sequences per sample (60% of the total dataset, the second round of subsampling; to diminish the effect of depth on PCoA, we only tested the effect of unstable OTUs on PCoA). To accomplish this, we employed the Bray-Curtis distance (a non-phylogenetic metric) and weighted UniFrac (a phylogenetic metric). Adonis was used to test whether the samples from the full dataset and the 60% subset clustered independently of each other and to quantify the size of that effect (this was performed by running compare_categories.py --method adonis -i dm.txt -m Map.txt -c Treatment -o adonis_out -n 999).

We used a proportion of unstable sequences, a proportion of unstable OTUs, and Matthews’s correlation coefficient (MCC) to quantify OTU stability. Unstable sequences were defined to include unique sequences that were represented by different OTU centroids at the different sequencing depths. Unstable OTUs were defined to include OTUs whose membership changed at different sequencing depths (in other words, if an OTU is composed of the same sequences at different sequencing depths, excluding sequences that were not included in the smaller subsample, then that OTU is defined as being stable). To compute the percent of unstable sequences and unstable OTUs, we compared the clustering result of the full dataset with that of the 60% subsample using different clustering methods to analyze 30 replicates for each method. If a unique sequence was represented by the same representative sequence in both the 60% and the full dataset (excluding sequences not present in the 60% dataset), we considered the sequence to be stable. Otherwise, the sequence was considered to be unstable. If an OTU in the 60% subsample contained all of the same sequences as the full dataset (not including any sequences not present in the 60% subsample), we considered the OTU to be stable. Otherwise, we considered the OTU to be unstable. We additionally grouped all of the OTUs according to the frequency of their centroid at counts of 1, 2, 3 to 5, 6 to 10 and higher than 10, for the purpose of evaluating the stability of each of these groups.

To compute MCC, we recorded how each pair of sequences was clustered in the 60% subsample and the full dataset. The MCC value is calculated as follows:

$$ \mathrm{M}\mathrm{C}\mathrm{C}=\frac{\left(\mathrm{T}\mathrm{P}\times \mathrm{T}\mathrm{N}-\mathrm{F}\mathrm{P}\times \mathrm{F}\mathrm{N}\right)}{\sqrt{\left(\mathrm{T}\mathrm{P}+\mathrm{F}\mathrm{P}\right)\times \left(\mathrm{T}\mathrm{P}+\mathrm{F}\mathrm{N}\right)\times \left(\mathrm{T}\mathrm{N}+\mathrm{F}\mathrm{P}\right)\times \left(\mathrm{T}\mathrm{N}+\mathrm{F}\mathrm{N}\right)}} $$

If two sequences were clustered together in the full dataset and also in the 60% subsample, we recorded it as a true positive (TP). If two sequences were clustered separately in the full dataset and also in the 60% subsample, we recorded it as a true negative (TN). If two sequences were clustered together in the full dataset but not in the 60% subsample, we recorded it as a false negative (FN). Finally, if two sequences were clustered separately in the full dataset but together in the 60% subsample, we recorded it as a false positive (FP). Higher MCC values indicate enhanced stability of the clustering method.

## Additional files

Additional file 1: Box 1. Definitions of OTU clustering algorithms and executing commands, as used in this paper. Additional file 2: Figure S1. PCoA based on the Bray-Curtis distance, comparing 20%, 40%, 60%, and 80% subsamples with the full datasets using CL. All of the subsamples were rarefied to 10,000 sequences per sample (20% of the full dataset) to be included in this analysis. Additional file 3: Figure S2. Phylum level composition, comparing 60% and full datasets using CL. All of the subsamples were rarefied to 30,000 sequences per sample (60% of the full dataset) to be included in this analysis. Additional file 4:
**Taxonomic composition from phylum to genus level, comparing 60% and full datasets using CL.** All of the subsamples were rarefied to 30,000 sequences per sample (60% of the full dataset) to be included in this analysis. Additional file 5: Figure S3. Cytoscape network diagram showing the changes in OTUs at 60% and 100% subsamples of datasets using the AL method. Red dots represent OTUs in the 60% dataset, and blue dots represent OTUs in the full dataset. The size of the OTU is in proportion to the frequency of the centroid or representative sequence in each OTU. OTUs that changed between datasets but that share the same sequences are linked, and the line width is in proportion to the number of shared sequences between the two OTUs. OTUs that are exactly the same in the two datasets are not shown in the picture, such that each dot in this figure represents an unstable OTU. Additional file 6: Tables S1 and S2
**Table 1.** Multiple comparisons of unstable sequences between different clustering methods after the Kruskal-Wallis test. **Table 2.** Multiple comparisons of MCC values between different clustering methods after the Kruskal-Wallis test. Additional file 7: Figure S6. Rarefaction curve analyses and percentage of changed OTUs with seven additional datasets (a1-f1) AGC; (a2-f2) closed-reference; (a3-f3) open-reference; (a4-f4) proportion of unstable sequences by method; (a5-f5) percentage of unstable OTUs for different centroid frequencies. (a1-a5) Illumina sequencing of V6 regions from Azorean shallow marine vents (SRX011425); (b1-b5) 454 sequencing of V3 V5 regions of HMP project data male stool (SRS011410); (c1-c5) 454 sequencing of V5 V6 regions from Little Sippewissett Marsh (SRX210127); (d1-d5) 454 sequencing of V3 region of a stool sample (SRP005150); (e1-e5) 454 sequencing of V3 region of a stool sample (SRS052471); (f1-f5) Illumina sequencing of V6 region (overlapped sequence) of a mangrove sediment sample (MG-RAST 4490068.3). Additional file 8: Figure S4. Principles underlying stable dereplication clustering, rarefaction curves, and PCoA based on the Bray-Curtis distance. (a) Principles underlying stable dereplication clustering at two sampling depths. White circles indicate individual sequences that were included in both the small and the large subsamples, and dark circles indicate sequences that were added only in the large subsample. Large circles in red or blue indicate OTUs in the small and the large subsamples, respectively. (b) Rarefaction curves generated with dereplication clustering at five different depths. (c) PCoA based on the Bray-Curtis distance, comparing 60% subsamples with the full datasets using de-replication clustering. All of the subsamples were rarefied to 30,000 sequences per sample to be included in this analysis. Additional file 9: Figure S5. Rarefaction curves and PCoA based on the Bray-Curtis distance. (a) Rarefaction curves generated with open-reference OTU clustering at five different depths. (b) PCoA based on the Bray-Curtis distance, comparing 60% subsamples with the full datasets using open-reference OTU clustering. All of the subsamples were rarefied to 30,000 sequences per sample to be included in this analysis. Additional file 10: Figure S7. PCoA with weighted UniFrac, comparing 60% subsamples with the full datasets using *de novo* clustering methods. All of the subsamples were rarefied to 30,000 sequences per sample to be included in this analysis.
