# Erratum to: Stability of operational taxonomic units: an important but neglected property for analyzing microbial diversity

**DOI:** 10.1186/s40168-015-0098-1

**Published:** 2015-07-31

**Authors:** Yan He, J. Gregory Caporaso, Xiao-Tao Jiang, Hua-Fang Sheng, Susan M. Huse, Jai Ram Rideout, Robert C. Edgar, Evguenia Kopylova, William A. Walters, Rob Knight, Hong-Wei Zhou

**Affiliations:** State Key Laboratory of Organ Failure Research, Department of Environmental Health, Guangdong Provincial Key Laboratory of Tropical Disease Research, School of Public Health and Tropical Medicine, Southern Medical University, Guangzhou, 510515 China; Department of Biological Sciences, Northern Arizona University, PO Box 5640, Flagstaff, AZ 86011-5640 USA; Center for Microbial Genetics and Genomics, Northern Arizona University, PO Box 4073, Flagstaff, AZ 86011-4073 USA; Department of Pathology and Laboratory Science, Warren Alpert Medical School, Brown University, 70 Ship Street, Providence, RI 02912 USA; Tiburon, CA 94920 USA; Department of Pediatrics, University of California at San Diego, 9500 Gilman Drive MC0763, La Jolla, CA 92093-0763 USA; Department of Molecular Biology and Genetics, Cornell University, 526 Campus Road, Ithaca, NY 14853 USA; Department of Computer Science and Engineering, University of California at San Diego, 9500 Gilman Drive MC0763, La Jolla, CA 92093-0763 USA

## Correction

After publication of this work [1], we noted that one of the references in the introduction was not described properly. With respect to the original authors, we clarify that this sentence in introduction: “Due to the lack of a gold standard of ‘correct’ OTUs, several measurements have been used to evaluate the performance of clustering methods, for example, rationality of OTU structure [2, 3], computational efficiency (that is, runtime and memory requirements) [4], and the ability to cope with OTU inflation [5]” should be stated as “Due to the lack of a gold standard of ‘correct’ OTUs, several measurements have been used to evaluate the performance of clustering methods, for example, rationality of OTU structure [2], use of the Matthew’s Correlation Coefficient [3], computational efficiency (that is, runtime and memory requirements) [4], and the ability to cope with OTU inflation [5]”.
