# Supplementary material for: Stability of operational taxonomic units: an important but neglected property for analyzing microbial diversity
Source: Microbiome. 2015 May 20;3:20. doi: 10.1186/s40168-015-0081-x (PMC4438525; doi:10.1186/s40168-015-0081-x)
Supplement: Additional file 1: Box 1. — Definitions of OTU clustering algorithms and executing commands, as used in this paper. [file 40168_2015_81_MOESM1_ESM.docx]

Stable operational taxonomic units for studying microbial ecology

He *et al.*

**Box 1: Definitions of OTU clustering algorithms and executing commands, as used in this paper.**

**complete-linkage (CL) clustering**

Sequences are clustered such that the distances between all pairs of sequences in a cluster is less than or equal to the similarity threshold.

cluster(column=unique.dist,name=names,cutoff=0.05,precision=1000,method=furthest)

**single-linkage (SL) clustering**

Sequences are clustered such that each sequence in a cluster has a distance less than or equal to the similarity threshold to at least one other sequence in the cluster.

cluster(column=unique.dist,name=names,cutoff=0.05,precision=1000,method=nearest)

**average-linkage (AL) clustering**

Sequences are clustered such each sequence in a cluster has an average distance less than or equal to the similarity threshold to all other sequences in the cluster.

cluster(column=unique.dist,name=names,cutoff=0.05,precision=1000,method=average)

**dereplication**

Sequences that are 100% identical in length and composition are clustered together.

***de novo* clustering**

Cluster centroids are chosen from the collection of query sequences. Sequences are clustered with a centroid if the distance between the sequence and the cluster centroid is less than or equal to a user-defined similarity threshold.

**distance-based greedy** ***de novo* clustering (DGC)**

An approach for *de novo* clustering where, when a sequence matches more than one cluster centroid at less than or equal to the similarity threshold, the sequence is clustered with the closest centroid.

pick_de_novo_otus.py -i fasta –o output_dir –p dgc.params.txt

content in dgc.params.txt:

pick_otus:otu_picking_method usearch61

pick_otus:max_accepts 16

pick_otus:max_rejects 64

pick_otus:enable_rev_strand_match True

pick_otus:minlen 30

**abundance-based greedy** ***de novo* clustering (AGC)**

An approach for *de novo* clustering where, when a sequence matches more than one cluster centroid at less than or equal to the similarity threshold, the sequence is clustered with the most abundant centroid (i.e., the centroid sequence which has been observed more times in the data set).

pick_de_novo_otus.py -i fasta –o output_dir –p agc.params.txt

content in agc.params.txt:

pick_otus:otu_picking_method usearch61

pick_otus:sizeorder True

pick_otus:max_accepts 16

pick_otus:max_rejects 64

pick_otus:enable_rev_strand_match True

pick_otus:minlen 30

**closed-reference clustering**

Sequences are clustered against an external reference database of centroid sequences, and assigned to a cluster if the distance between a sequence and a reference centroid is less than or equal to a user-defined similarity threshold. Sequences that fail to hit a reference centroid are discarded. When a sequence matches more than one cluster centroid at less than or equal to the similarity threshold, the sequence is clustered with the centroid to which it has the smaller distance.

pick_closed_reference_otus.py -i fasta -o output_dir –r reference_dataset -p closedref.params.txt

content in closedref.params.txt:

pick_otus:otu_picking_method usearch61_ref

pick_otus:max_accepts 16

pick_otus:max_rejects 64

pick_otus:enable_rev_strand_match True

pick_otus:minlen 30

**open-reference clustering**

Sequences are clustered against an external reference database of centroid sequences, and assigned to a cluster if the distance between a sequence and a reference centroid is less than or equal to a user-defined similarity threshold. Sequences that fail to hit a reference centroid are subsequently clustered de novo. The present study uses AGC for de novo clustering, as it is the most stable methods compared to other *de novo* methods.

pick_open_reference_otus.py -i fasta -o output_dir -m usearch61 -r reference_dataset -s 1 -p openref.params.txt --min_otu_size 1 --prefilter_percent_id 0.0

openref.param.txt:

pick_otus:sizeorder True

pick_otus:max_accepts 16

pick_otus:max_rejects 64

pick_otus:enable_rev_strand_match True
