# Supplementary figures and images for: Stability of operational taxonomic units: an important but neglected property for analyzing microbial diversity
Source: Microbiome. 2015 May 20;3:20. doi: 10.1186/s40168-015-0081-x (PMC4438525; doi:10.1186/s40168-015-0081-x)

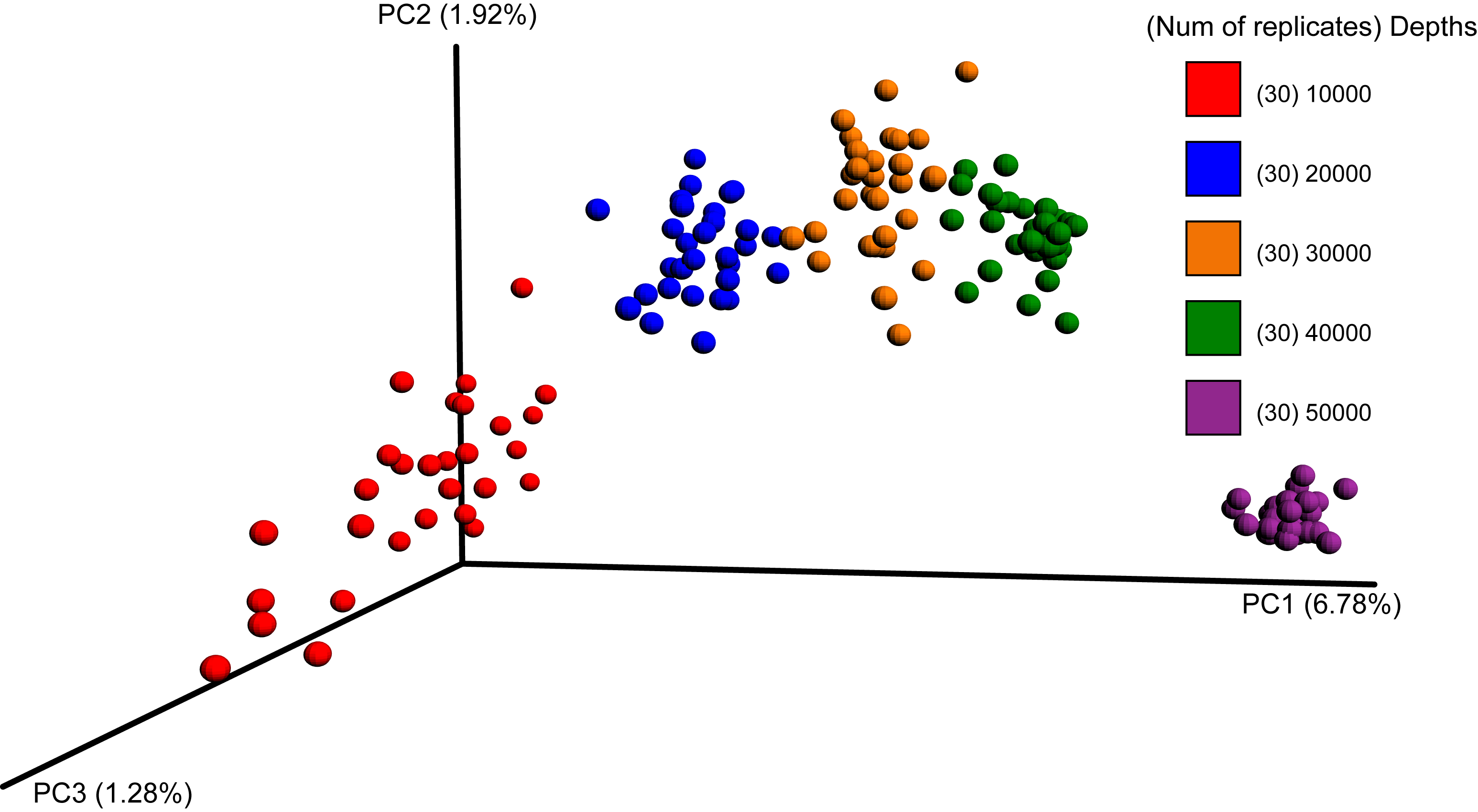

Supplement: Additional file 2: Figure S1. — PCoA based on the Bray-Curtis distance, comparing 20%, 40%, 60%, and 80% subsamples with the full datasets using CL. All of the subsamples were rarefied to 10,000 sequences per sample (20% of the full dataset) to be included in this analysis. [file 40168_2015_81_MOESM2_ESM.tiff]

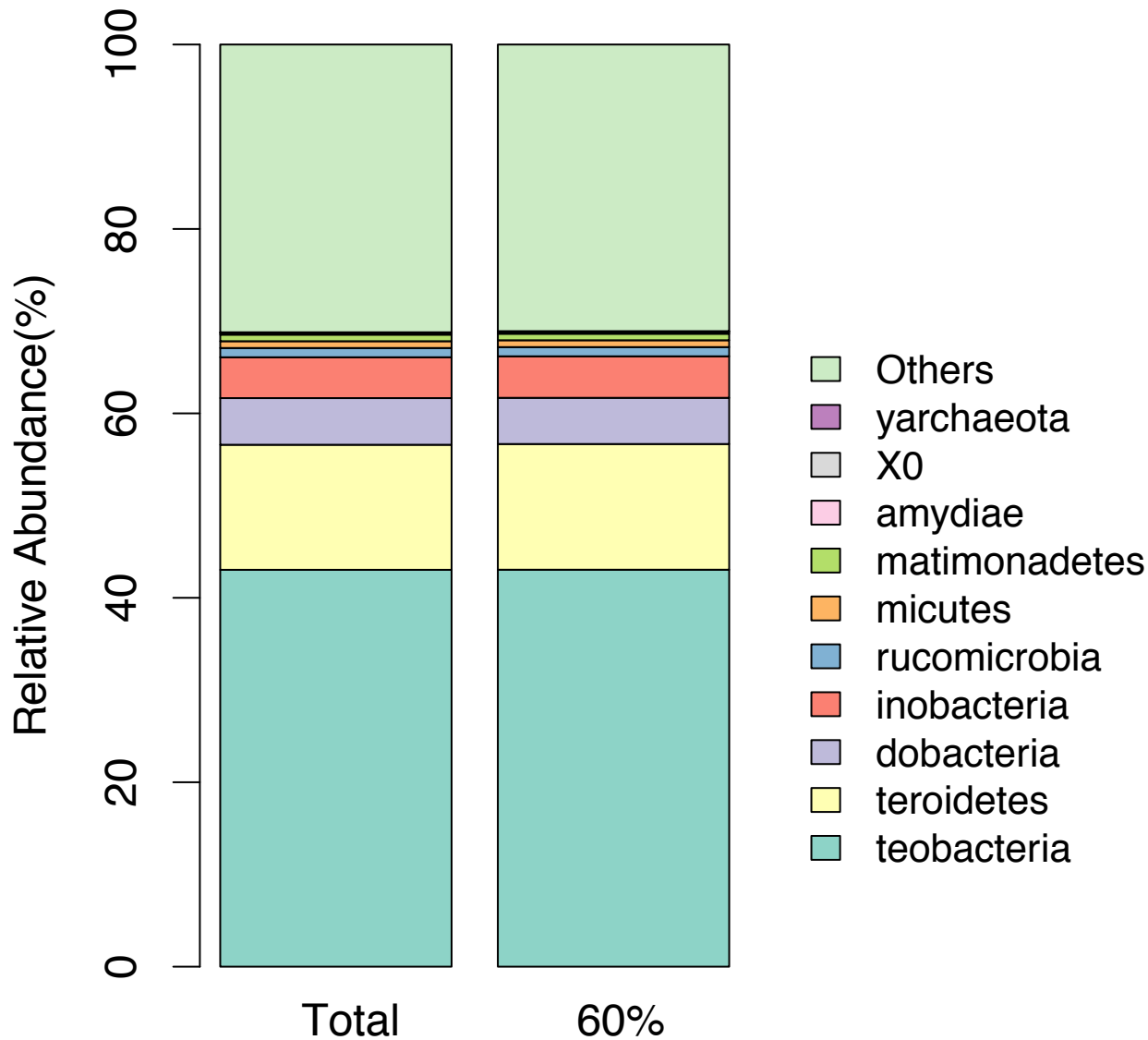

Supplement: Additional file 3: Figure S2. — Phylum level composition, comparing 60% and full datasets using CL. All of the subsamples were rarefied to 30,000 sequences per sample (60% of the full dataset) to be included in this analysis. [file 40168_2015_81_MOESM3_ESM.pdf]

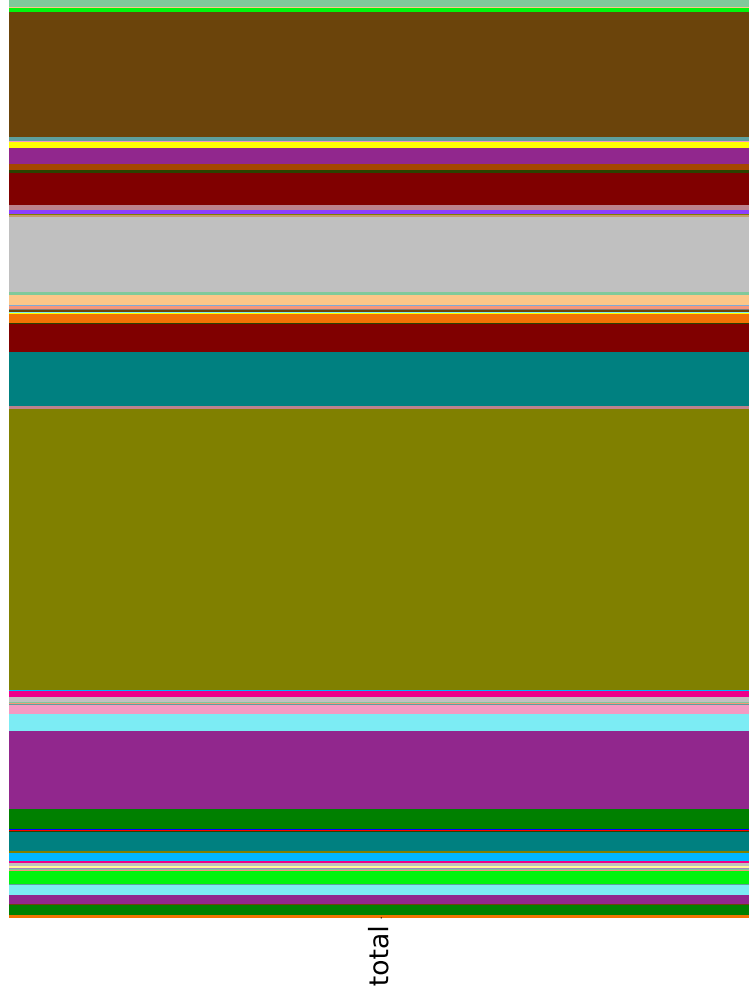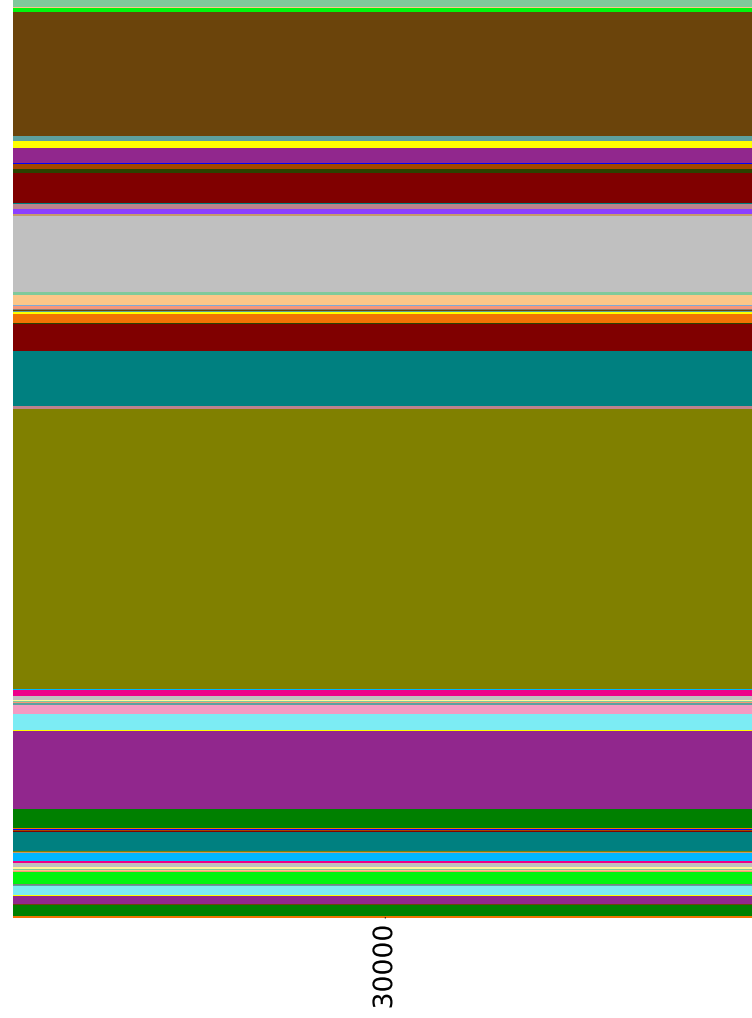

Supplement: Additional file 4: — Taxonomic composition from phylum to genus level, comparing 60% and full datasets using CL. All of the subsamples were rarefied to 30,000 sequences per sample (60% of the full dataset) to be included in this analysis. [file 40168_2015_81_MOESM4_ESM.zip › taxa_summary_plots/charts/8k7dxfRgKflkct7RocEuzIPR4WFqpU.pdf]

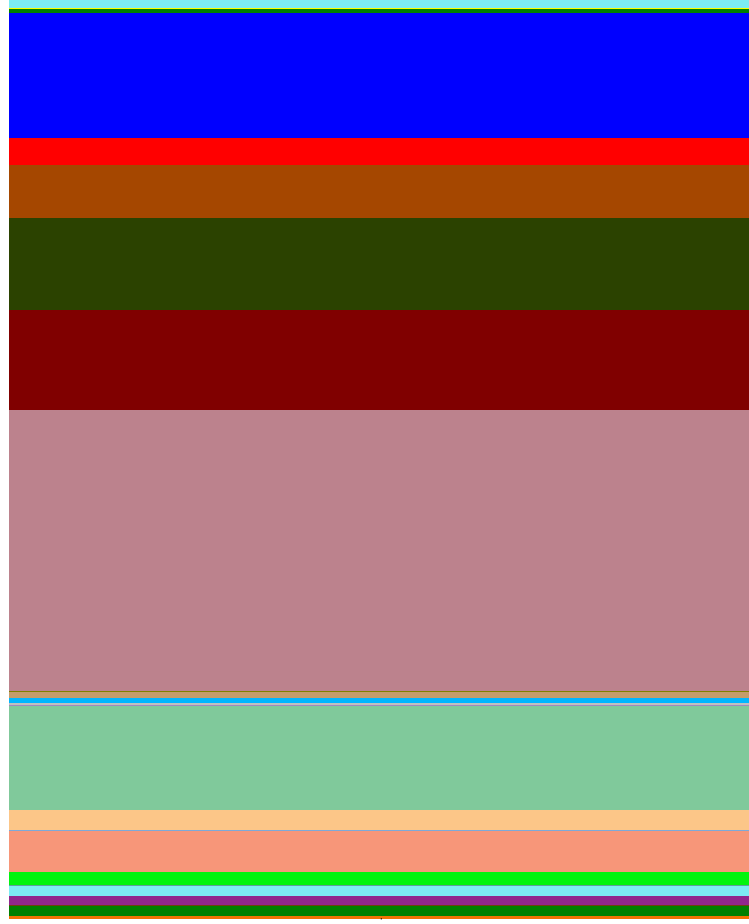

total

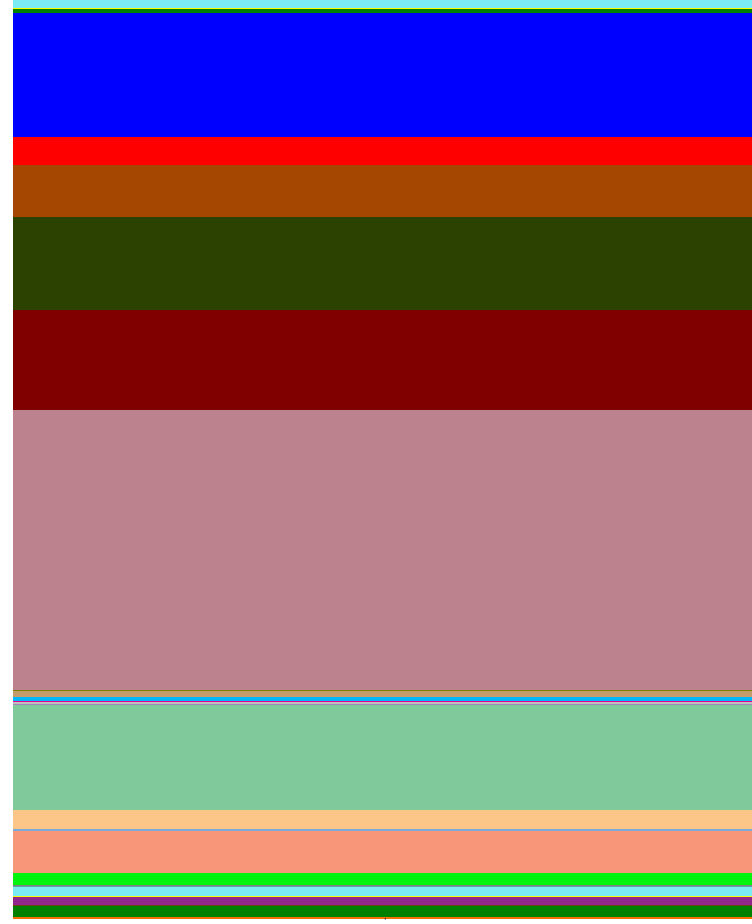

30000

Supplement: Additional file 4: — Taxonomic composition from phylum to genus level, comparing 60% and full datasets using CL. All of the subsamples were rarefied to 30,000 sequences per sample (60% of the full dataset) to be included in this analysis. [file 40168_2015_81_MOESM4_ESM.zip › taxa_summary_plots/charts/atIb36Wj0xLmU5OMOYWOqisgxZz8jU.pdf]

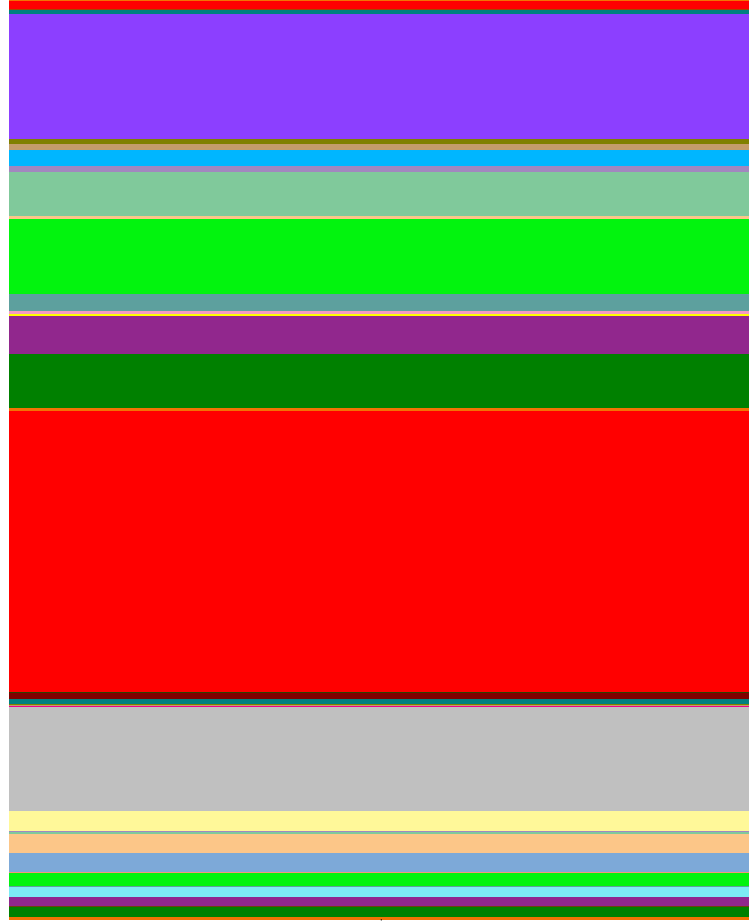

total

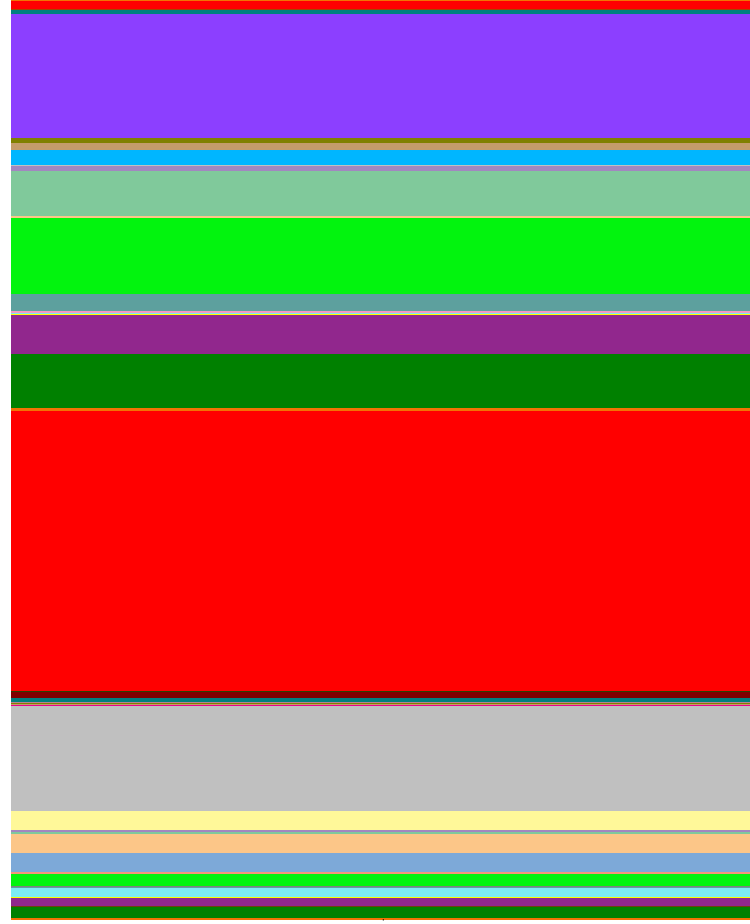

30000

Supplement: Additional file 4: — Taxonomic composition from phylum to genus level, comparing 60% and full datasets using CL. All of the subsamples were rarefied to 30,000 sequences per sample (60% of the full dataset) to be included in this analysis. [file 40168_2015_81_MOESM4_ESM.zip › taxa_summary_plots/charts/bDaO8tJyTUWKGcppdoRPptS3Xddb92.pdf]

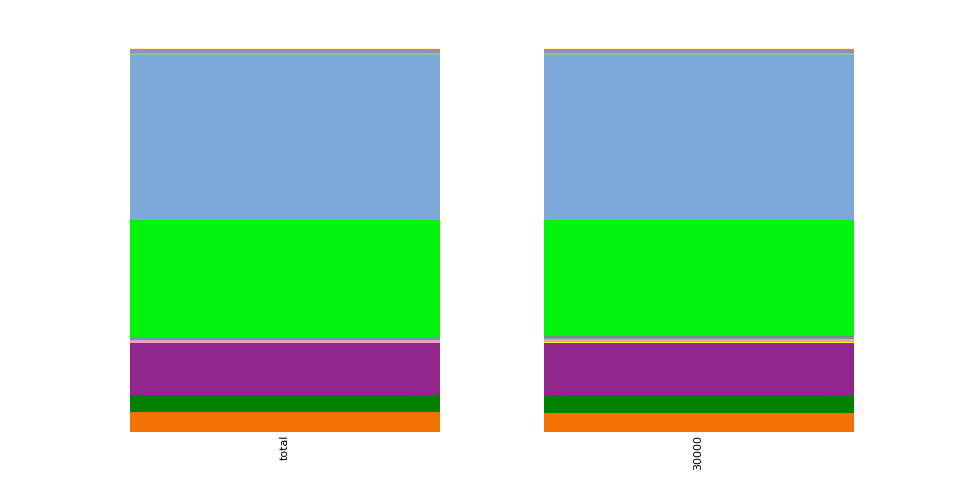

Supplement: Additional file 4: — Taxonomic composition from phylum to genus level, comparing 60% and full datasets using CL. All of the subsamples were rarefied to 30,000 sequences per sample (60% of the full dataset) to be included in this analysis. [file 40168_2015_81_MOESM4_ESM.zip › taxa_summary_plots/charts/hf670wKSbglWONZT8pChIUUaa0gc4r.png]

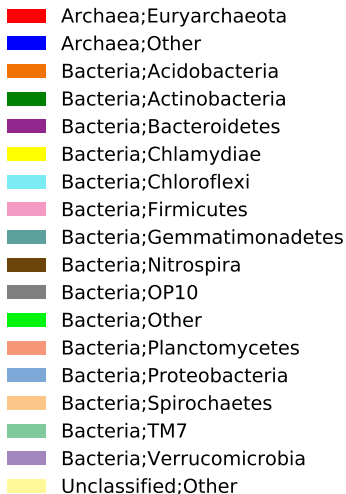

Supplement: Additional file 4: — Taxonomic composition from phylum to genus level, comparing 60% and full datasets using CL. All of the subsamples were rarefied to 30,000 sequences per sample (60% of the full dataset) to be included in this analysis. [file 40168_2015_81_MOESM4_ESM.zip › taxa_summary_plots/charts/hf670wKSbglWONZT8pChIUUaa0gc4r_legend.pdf]

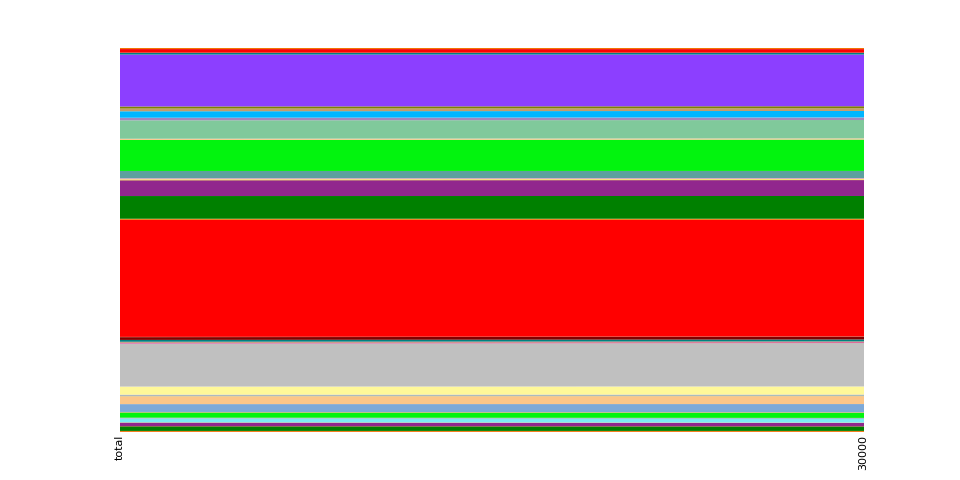

Supplement: Additional file 4: — Taxonomic composition from phylum to genus level, comparing 60% and full datasets using CL. All of the subsamples were rarefied to 30,000 sequences per sample (60% of the full dataset) to be included in this analysis. [file 40168_2015_81_MOESM4_ESM.zip › taxa_summary_plots/charts/hFil570eejJ8DnmNhaYwtifCTk1CDc.png]

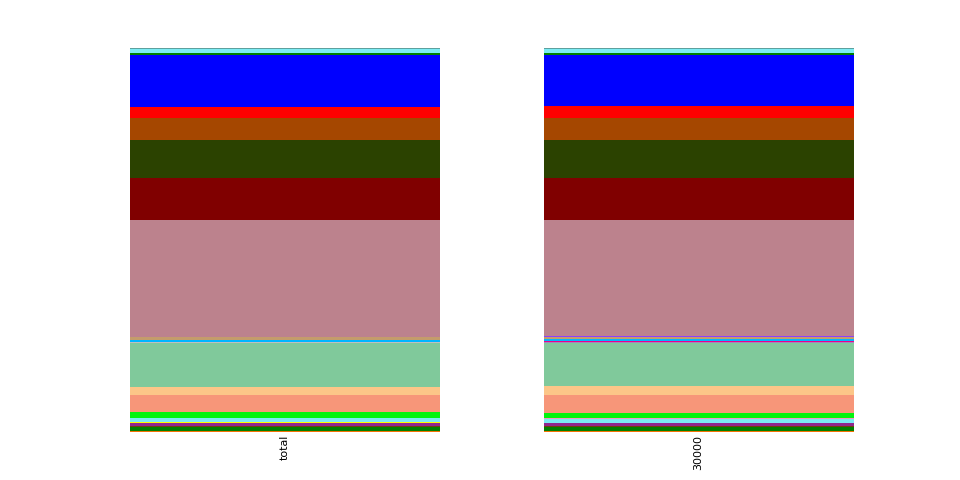

Supplement: Additional file 4: — Taxonomic composition from phylum to genus level, comparing 60% and full datasets using CL. All of the subsamples were rarefied to 30,000 sequences per sample (60% of the full dataset) to be included in this analysis. [file 40168_2015_81_MOESM4_ESM.zip › taxa_summary_plots/charts/K3qZYZ0up21F32Dsgr7nHTQFP37H0g.png]

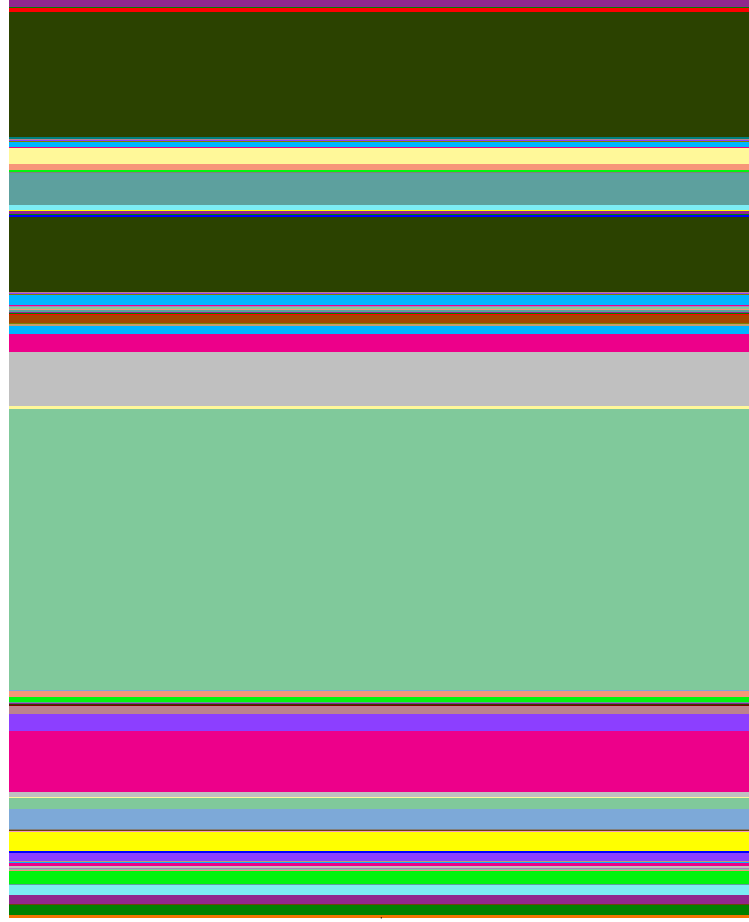

total

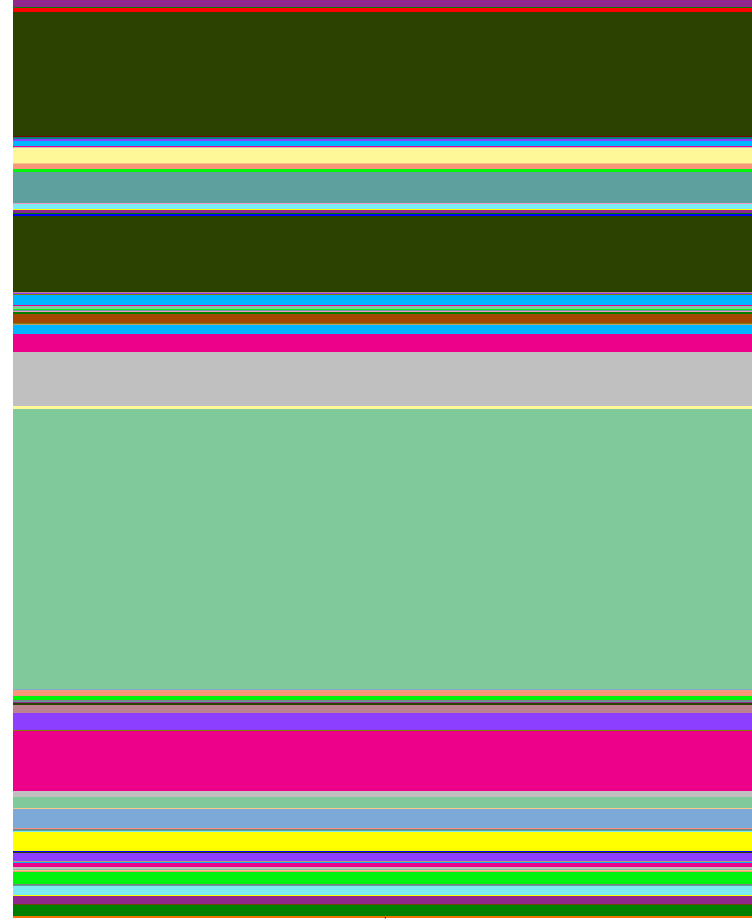

30000

Supplement: Additional file 4: — Taxonomic composition from phylum to genus level, comparing 60% and full datasets using CL. All of the subsamples were rarefied to 30,000 sequences per sample (60% of the full dataset) to be included in this analysis. [file 40168_2015_81_MOESM4_ESM.zip › taxa_summary_plots/charts/kp90ZfCJrbSq110nNPUOwSZhzZsJq1.pdf]

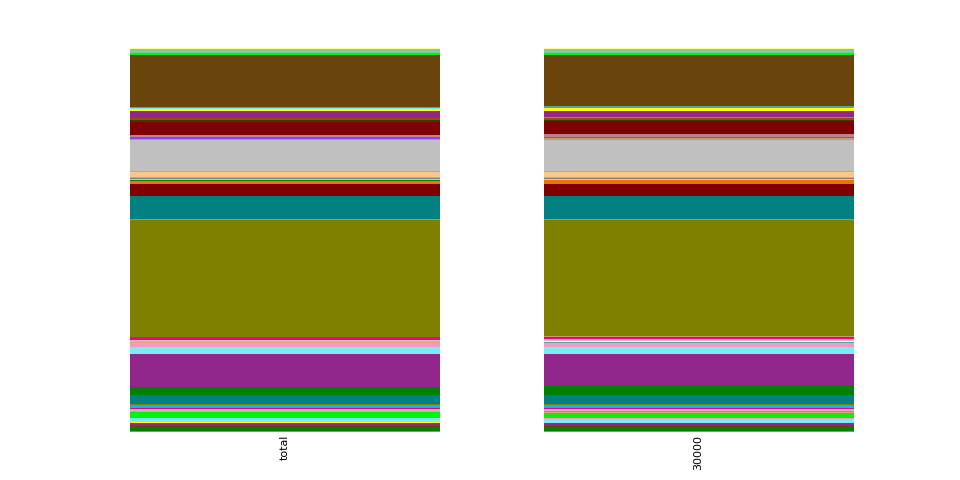

Supplement: Additional file 4: — Taxonomic composition from phylum to genus level, comparing 60% and full datasets using CL. All of the subsamples were rarefied to 30,000 sequences per sample (60% of the full dataset) to be included in this analysis. [file 40168_2015_81_MOESM4_ESM.zip › taxa_summary_plots/charts/Liij9PGrAWTg0zzZJksuYGI0OszmZ8.png]

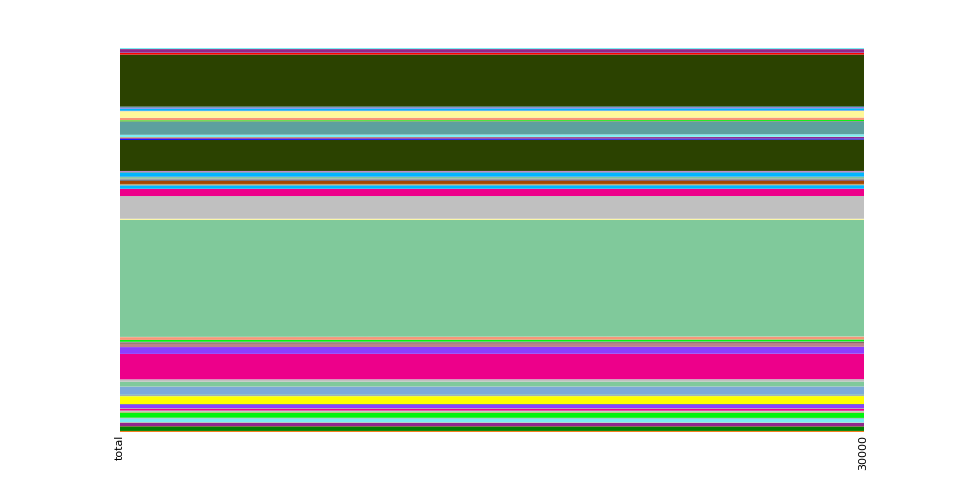

Supplement: Additional file 4: — Taxonomic composition from phylum to genus level, comparing 60% and full datasets using CL. All of the subsamples were rarefied to 30,000 sequences per sample (60% of the full dataset) to be included in this analysis. [file 40168_2015_81_MOESM4_ESM.zip › taxa_summary_plots/charts/nzI3lUHt8zstgmO0xSpzzfZZ2Gm1NW.png]

total

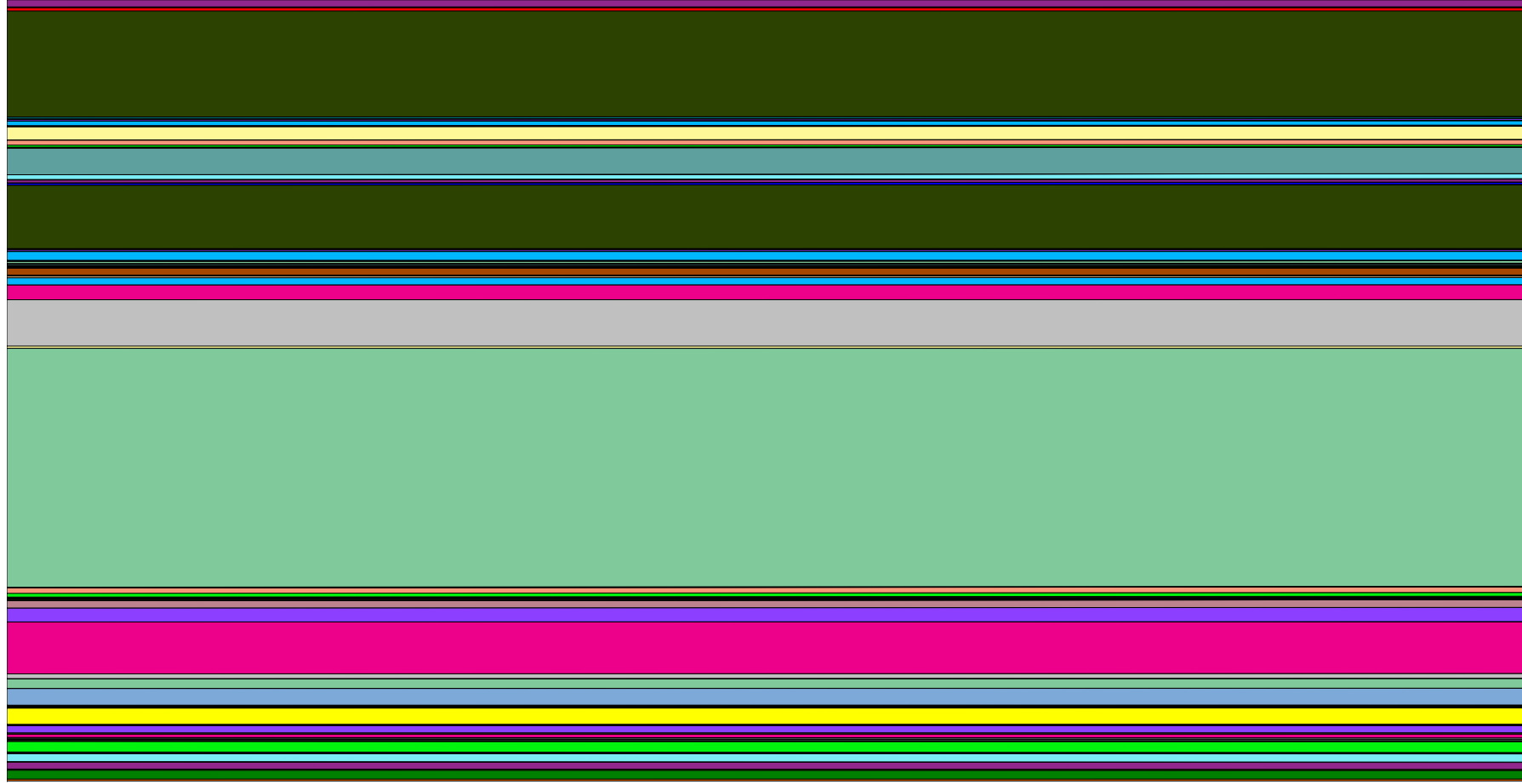

30000

Supplement: Additional file 4: — Taxonomic composition from phylum to genus level, comparing 60% and full datasets using CL. All of the subsamples were rarefied to 30,000 sequences per sample (60% of the full dataset) to be included in this analysis. [file 40168_2015_81_MOESM4_ESM.zip › taxa_summary_plots/charts/OccEOjRtxLoo2K0MNc9jkWwrsdJGpF.pdf]

total

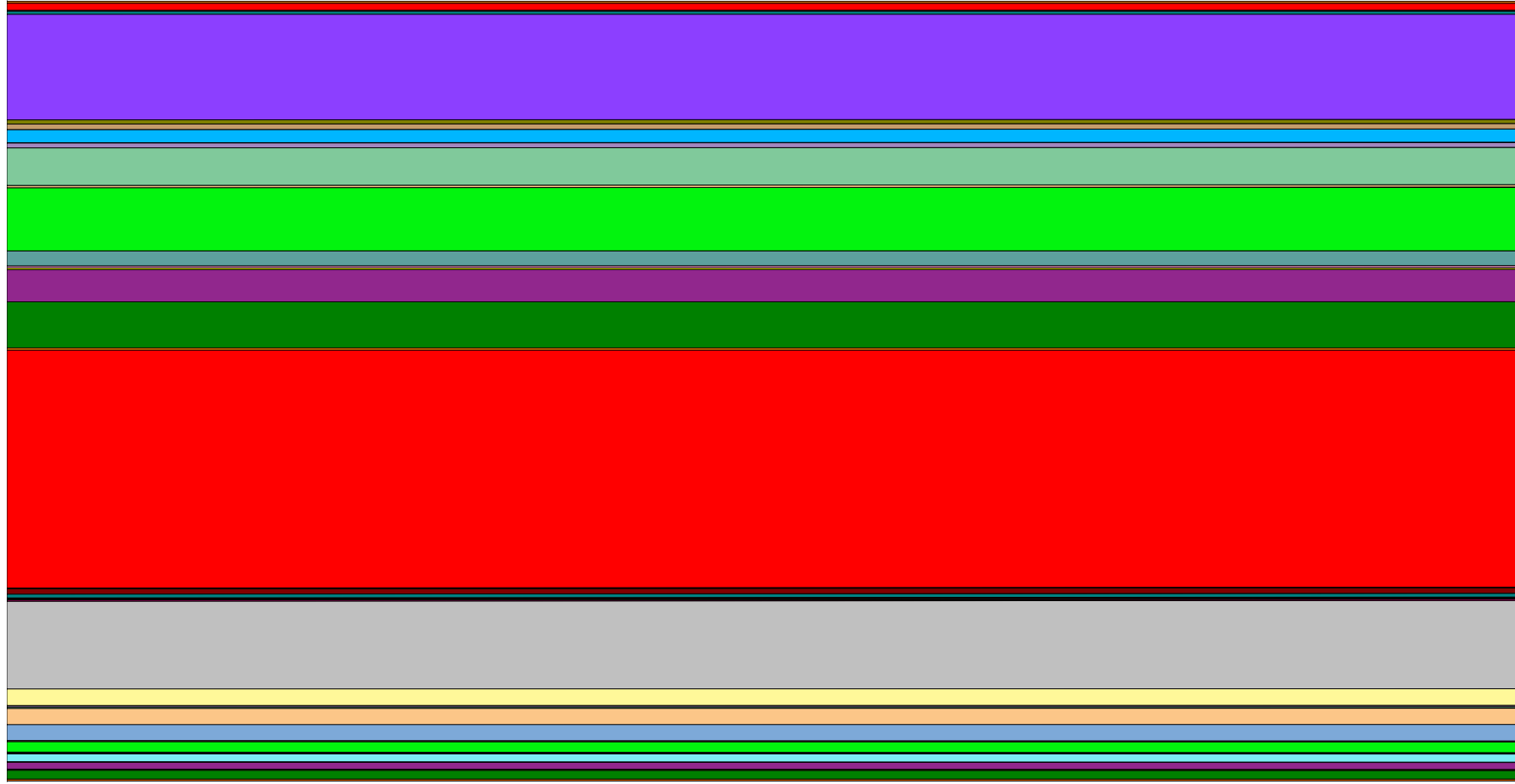

30000

Supplement: Additional file 4: — Taxonomic composition from phylum to genus level, comparing 60% and full datasets using CL. All of the subsamples were rarefied to 30,000 sequences per sample (60% of the full dataset) to be included in this analysis. [file 40168_2015_81_MOESM4_ESM.zip › taxa_summary_plots/charts/OkhtHFbUfC9ZTFhSHU0oCg0ZDOIszO.pdf]

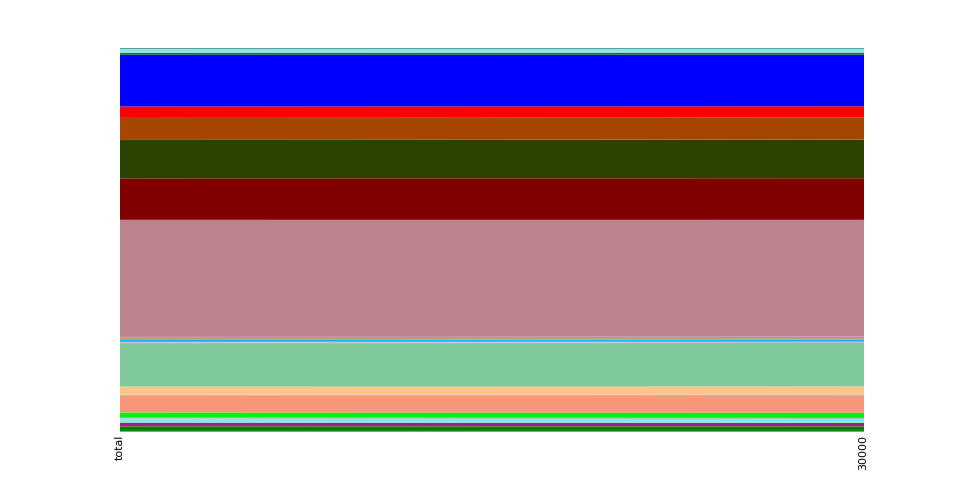

Supplement: Additional file 4: — Taxonomic composition from phylum to genus level, comparing 60% and full datasets using CL. All of the subsamples were rarefied to 30,000 sequences per sample (60% of the full dataset) to be included in this analysis. [file 40168_2015_81_MOESM4_ESM.zip › taxa_summary_plots/charts/OkI2Rm0mCjbKaij1mhXOkRiJd2gCcg.png]

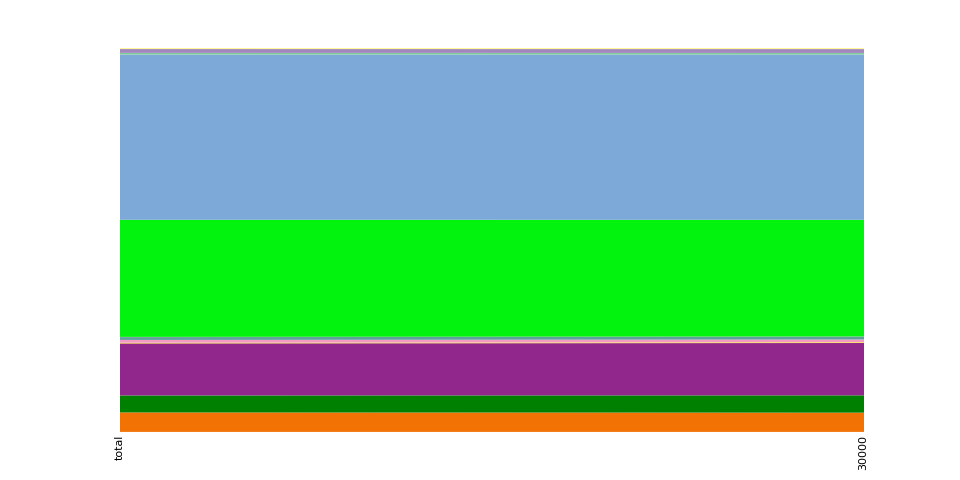

Supplement: Additional file 4: — Taxonomic composition from phylum to genus level, comparing 60% and full datasets using CL. All of the subsamples were rarefied to 30,000 sequences per sample (60% of the full dataset) to be included in this analysis. [file 40168_2015_81_MOESM4_ESM.zip › taxa_summary_plots/charts/PTPj1w01at8zLfj00t50G0pR2wXwo4.png]

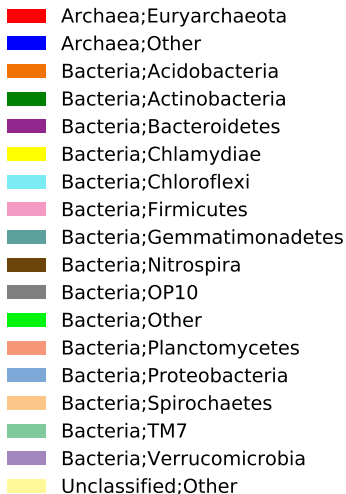

Supplement: Additional file 4: — Taxonomic composition from phylum to genus level, comparing 60% and full datasets using CL. All of the subsamples were rarefied to 30,000 sequences per sample (60% of the full dataset) to be included in this analysis. [file 40168_2015_81_MOESM4_ESM.zip › taxa_summary_plots/charts/PTPj1w01at8zLfj00t50G0pR2wXwo4_legend.pdf]

total

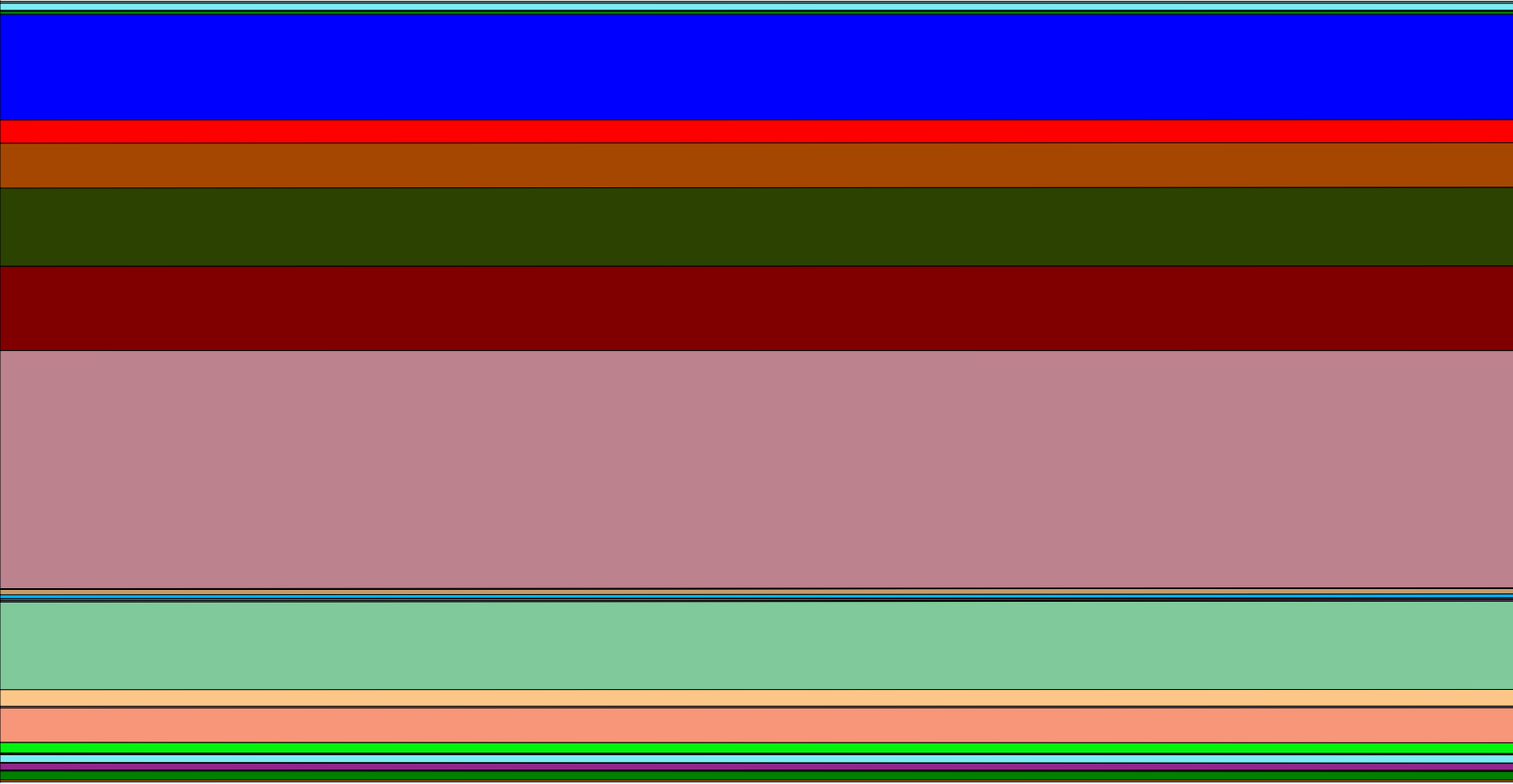

30000

Supplement: Additional file 4: — Taxonomic composition from phylum to genus level, comparing 60% and full datasets using CL. All of the subsamples were rarefied to 30,000 sequences per sample (60% of the full dataset) to be included in this analysis. [file 40168_2015_81_MOESM4_ESM.zip › taxa_summary_plots/charts/PxYS1LQDQDtwl1Ua6KCsyyTZ8e95CU.pdf]

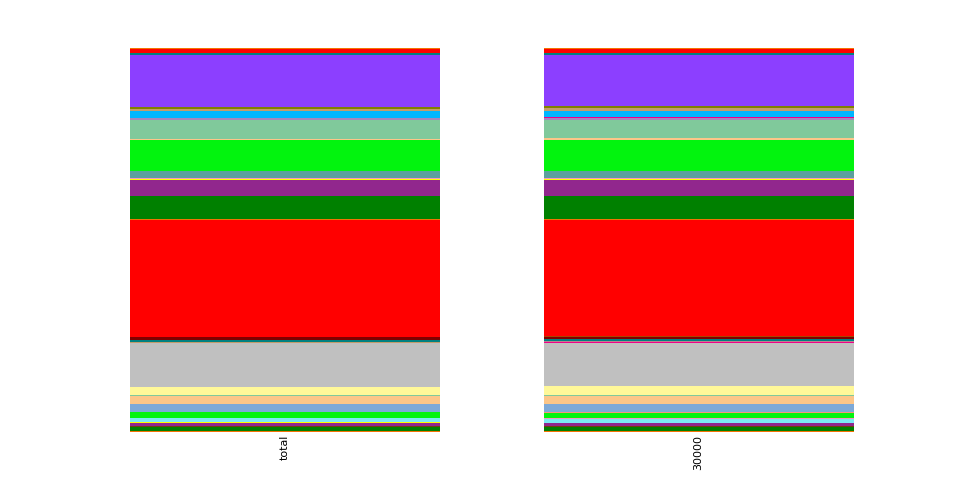

Supplement: Additional file 4: — Taxonomic composition from phylum to genus level, comparing 60% and full datasets using CL. All of the subsamples were rarefied to 30,000 sequences per sample (60% of the full dataset) to be included in this analysis. [file 40168_2015_81_MOESM4_ESM.zip › taxa_summary_plots/charts/SBD5aLcQ5MNjcnTZukNiRZU0jgzo2j.png]

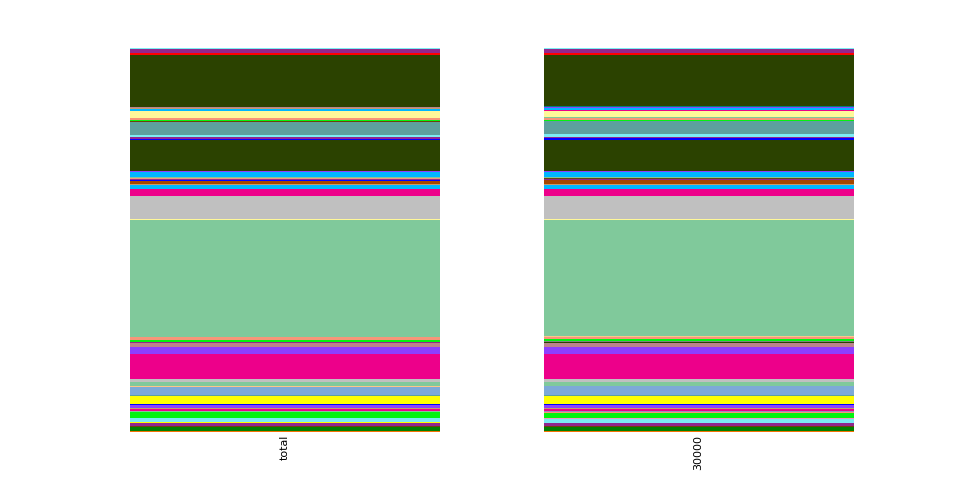

Supplement: Additional file 4: — Taxonomic composition from phylum to genus level, comparing 60% and full datasets using CL. All of the subsamples were rarefied to 30,000 sequences per sample (60% of the full dataset) to be included in this analysis. [file 40168_2015_81_MOESM4_ESM.zip › taxa_summary_plots/charts/U1OMuJLTM2RUABdjYb4p4hGsLemhBl.png]

total

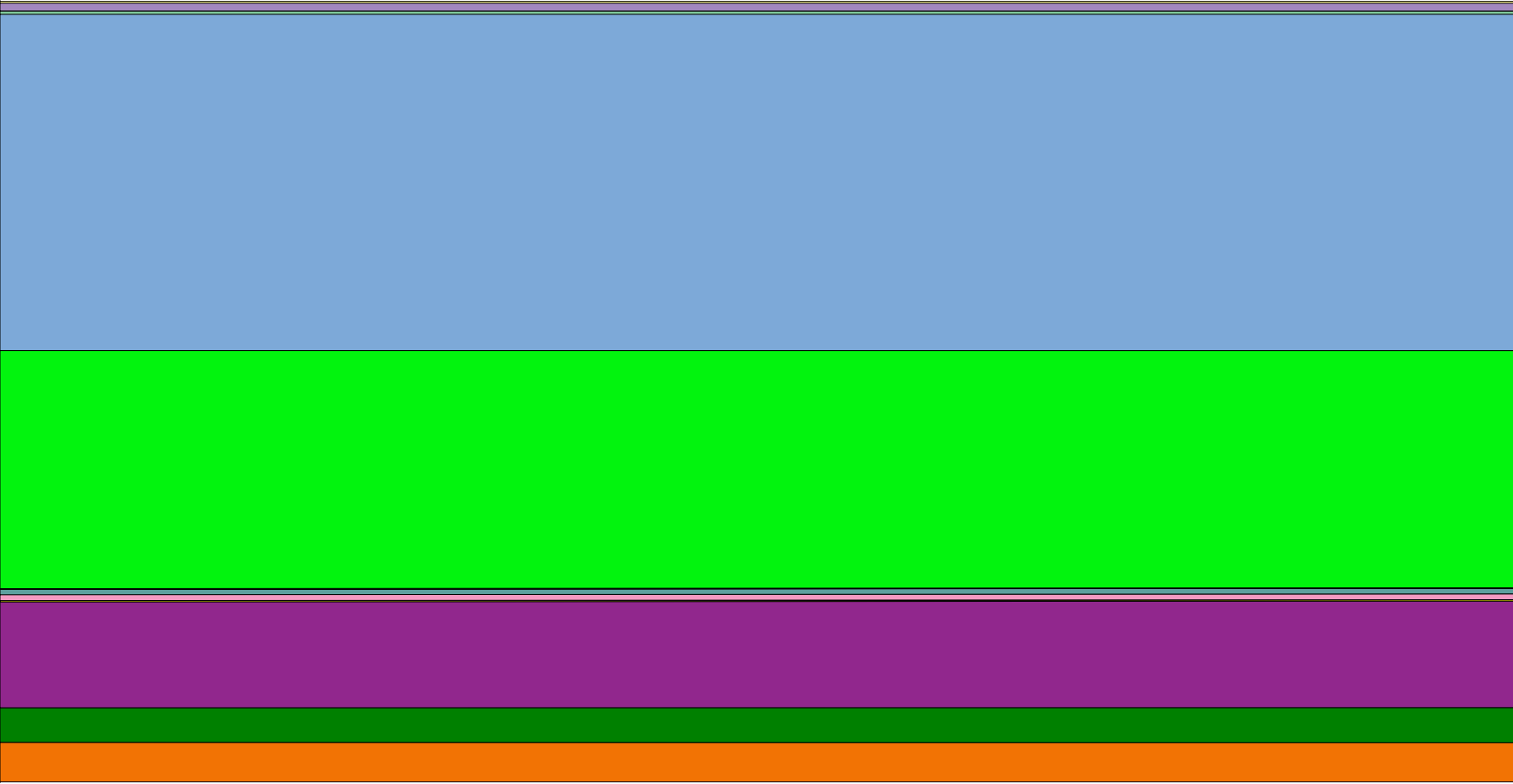

30000

Supplement: Additional file 4: — Taxonomic composition from phylum to genus level, comparing 60% and full datasets using CL. All of the subsamples were rarefied to 30,000 sequences per sample (60% of the full dataset) to be included in this analysis. [file 40168_2015_81_MOESM4_ESM.zip › taxa_summary_plots/charts/UW1K0NdgNl1LCHKBmqaT0IjkA0YylH.pdf]

total

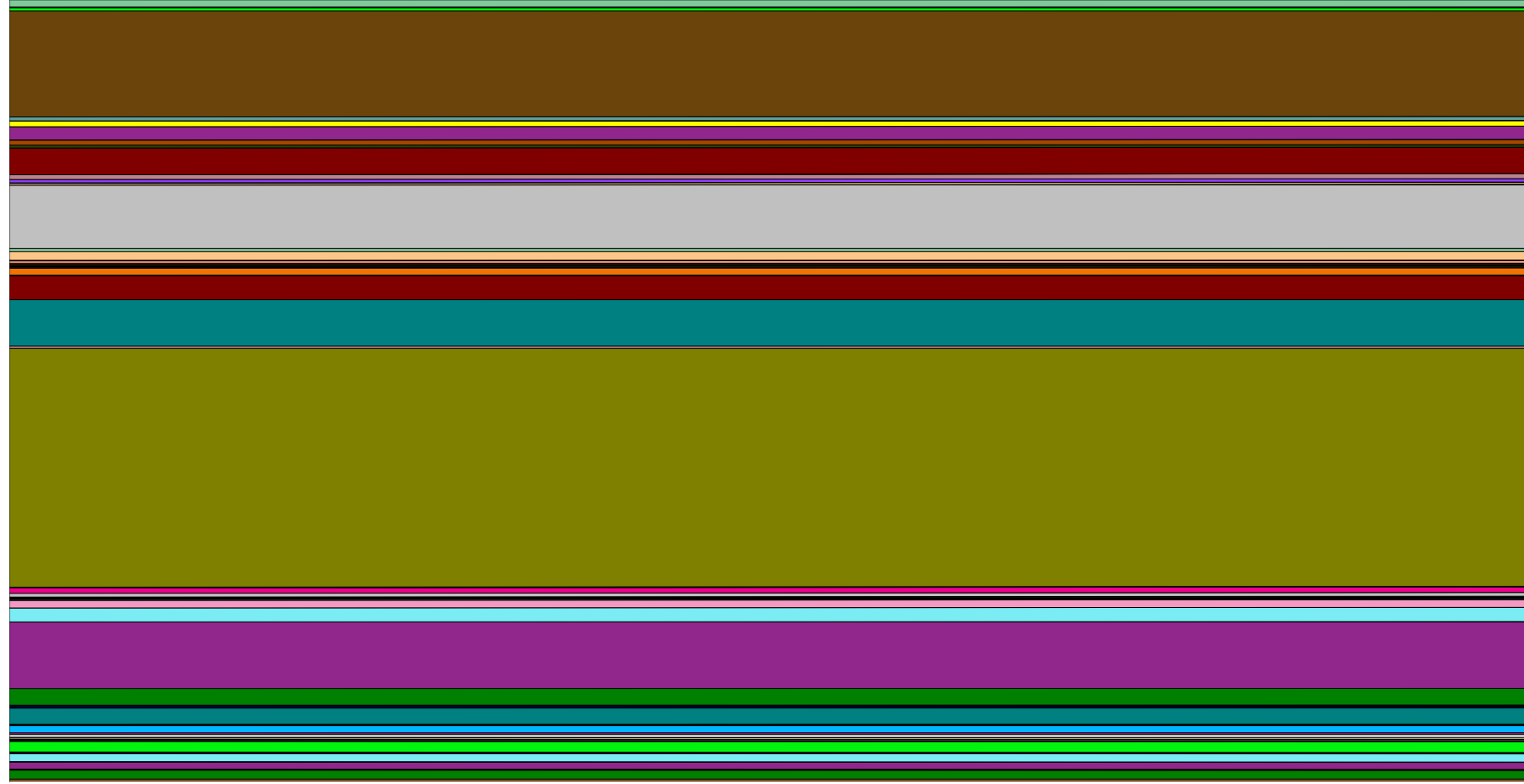

30000

Supplement: Additional file 4: — Taxonomic composition from phylum to genus level, comparing 60% and full datasets using CL. All of the subsamples were rarefied to 30,000 sequences per sample (60% of the full dataset) to be included in this analysis. [file 40168_2015_81_MOESM4_ESM.zip › taxa_summary_plots/charts/zC5Jk3iIc605qyP2MXswRe0y0UsbOc.pdf]

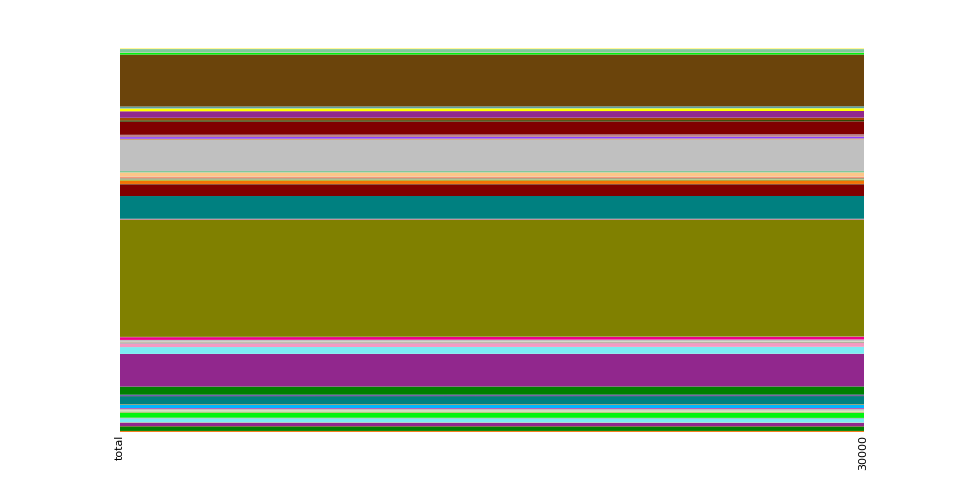

Supplement: Additional file 4: — Taxonomic composition from phylum to genus level, comparing 60% and full datasets using CL. All of the subsamples were rarefied to 30,000 sequences per sample (60% of the full dataset) to be included in this analysis. [file 40168_2015_81_MOESM4_ESM.zip › taxa_summary_plots/charts/ZJKaAcRrGK6PIfWygzzx67CF9O17c3.png]

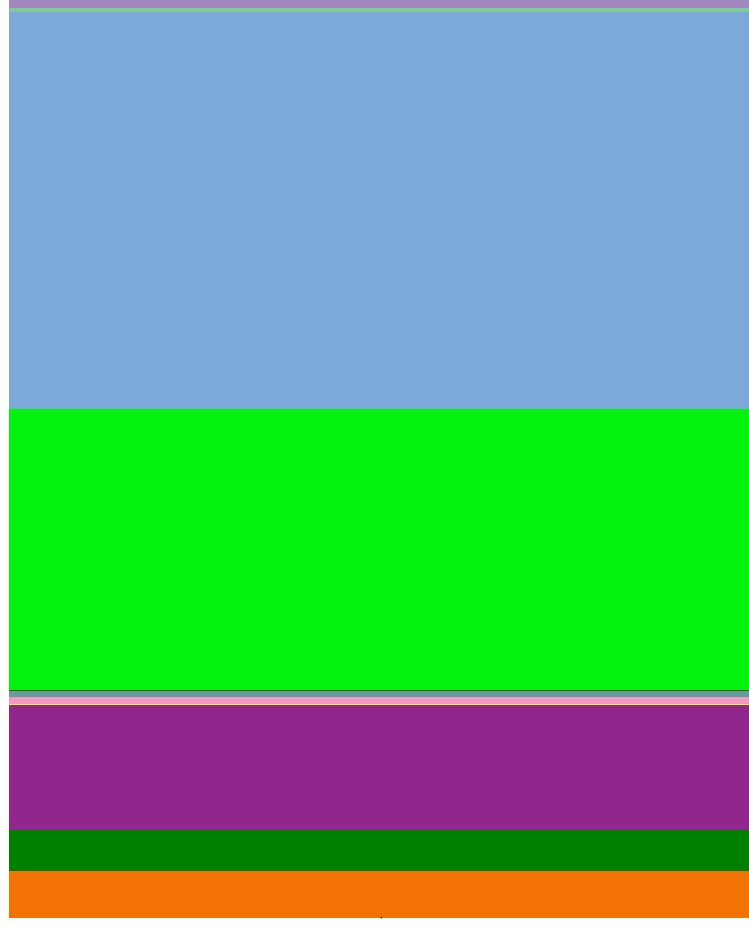

total

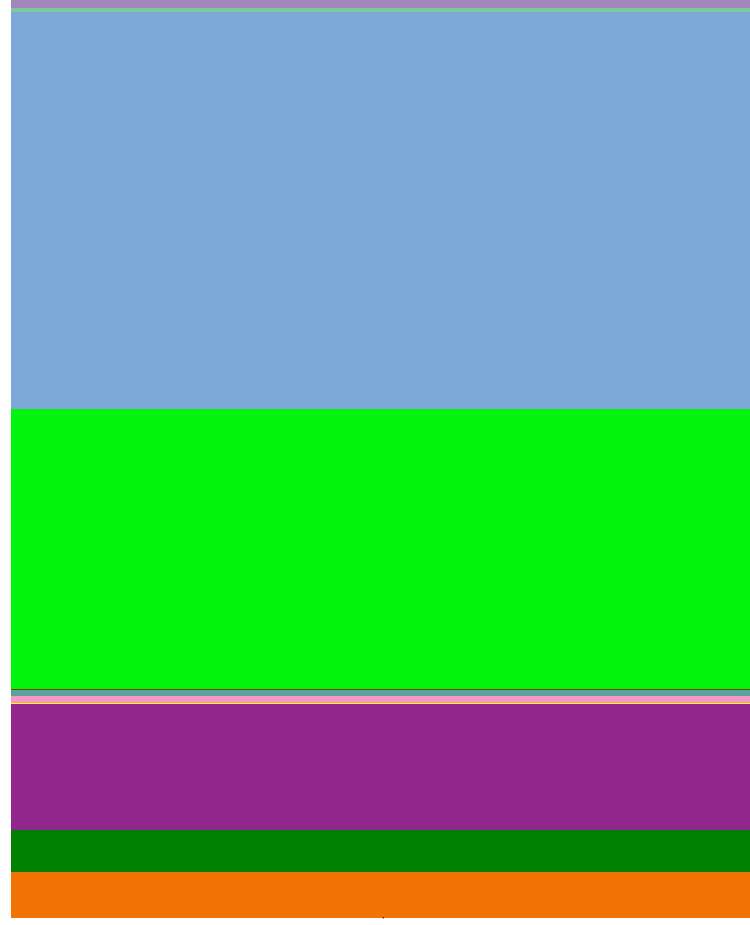

30000

Supplement: Additional file 4: — Taxonomic composition from phylum to genus level, comparing 60% and full datasets using CL. All of the subsamples were rarefied to 30,000 sequences per sample (60% of the full dataset) to be included in this analysis. [file 40168_2015_81_MOESM4_ESM.zip › taxa_summary_plots/charts/zQYpNuozUIAfSKixW0YlbPM5ZFnYZB.pdf]

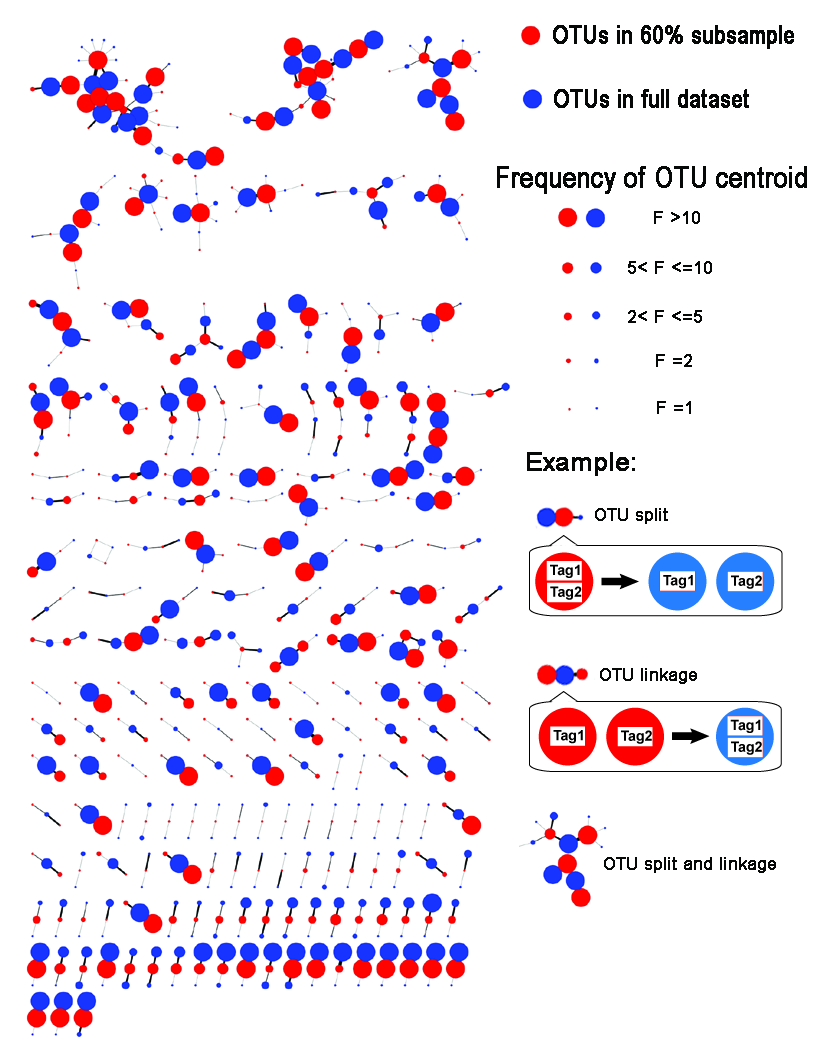

Supplement: Additional file 5: Figure S3. — Cytoscape network diagram showing the changes in OTUs at 60% and 100% subsamples of datasets using the AL method. Red dots represent OTUs in the 60% dataset, and blue dots represent OTUs in the full dataset. The size of the OTU is in proportion to the frequency of the centroid or representative sequence in each OTU. OTUs that changed between datasets but that share the same sequences are linked, and the line width is in proportion to the number of shared sequences between the two OTUs. OTUs that are exactly the same in the two datasets are not shown in the picture, such that each dot in this figure represents an unstable OTU. [file 40168_2015_81_MOESM5_ESM.tiff]

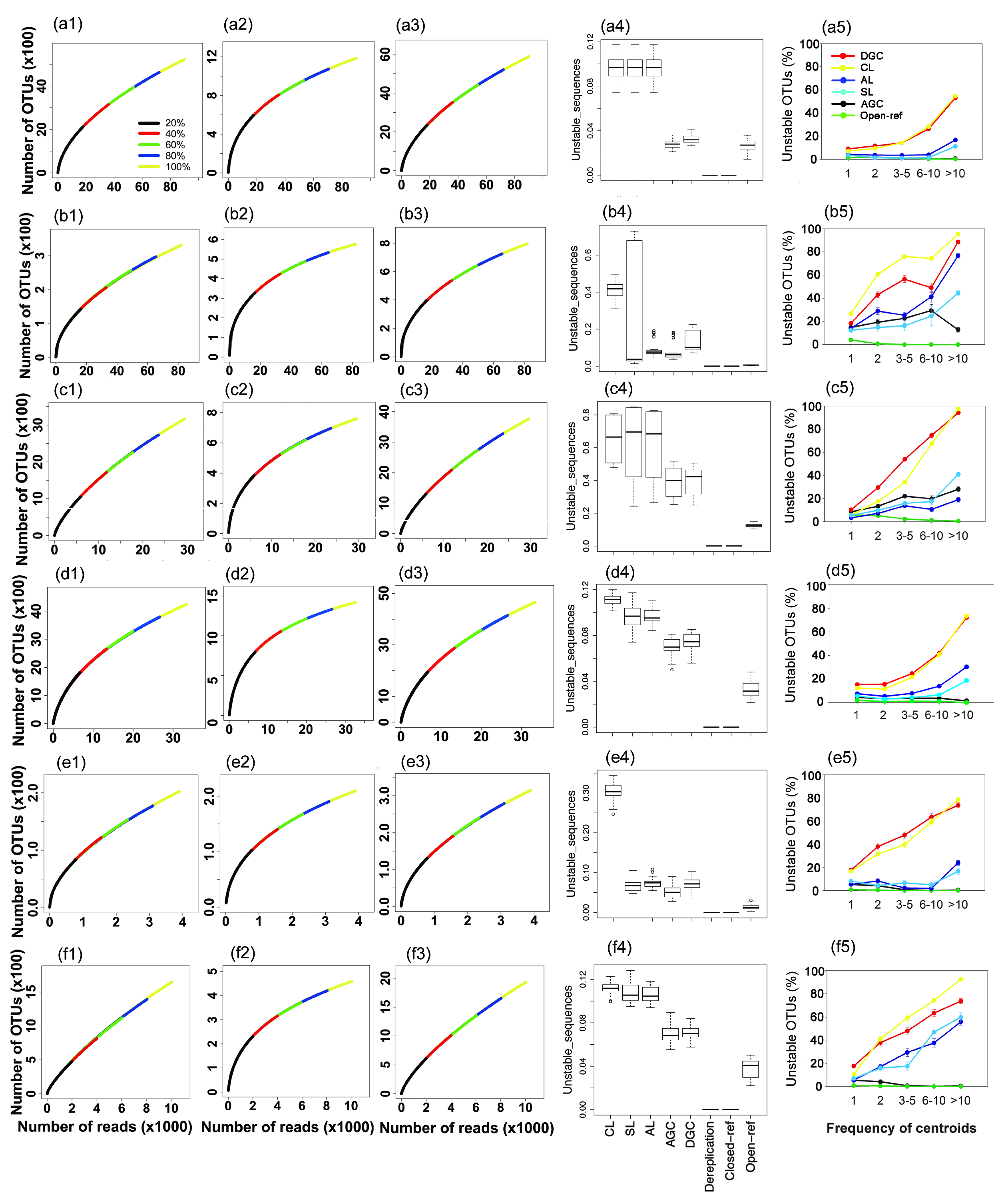

Supplement: Additional file 7: Figure S6. — Rarefaction curve analyses and percentage of changed OTUs with seven additional datasets (a1-f1) AGC; (a2-f2) closed-reference; (a3-f3) open-reference; (a4-f4) proportion of unstable sequences by method; (a5-f5) percentage of unstable OTUs for different centroid frequencies. (a1-a5) Illumina sequencing of V6 regions from Azorean shallow marine vents (SRX011425); (b1-b5) 454 sequencing of V3 V5 regions of HMP project data male stool (SRS011410); (c1-c5) 454 sequencing of V5 V6 regions from Little Sippewissett Marsh (SRX210127); (d1-d5) 454 sequencing of V3 region of a stool sample (SRP005150); (e1-e5) 454 sequencing of V3 region of a stool sample (SRS052471); (f1-f5) Illumina sequencing of V6 region (overlapped sequence) of a mangrove sediment sample (MG-RAST 4490068.3). [file 40168_2015_81_MOESM7_ESM.tiff]

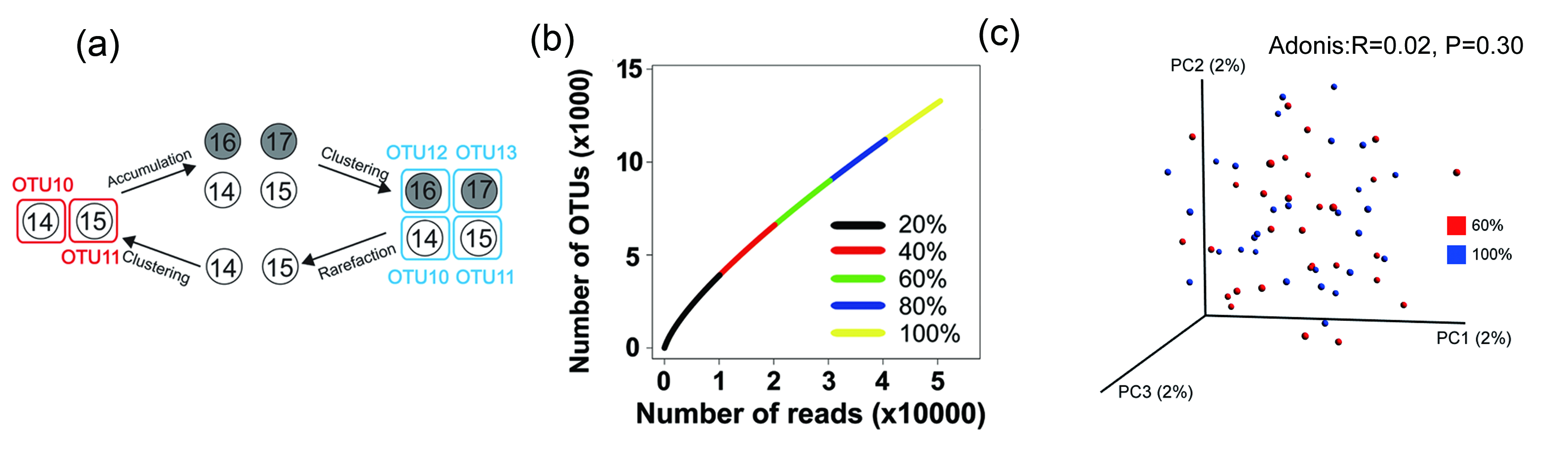

Supplement: Additional file 8: Figure S4. — Principles underlying stable dereplication clustering, rarefaction curves, and PCoA based on the Bray-Curtis distance. (a) Principles underlying stable dereplication clustering at two sampling depths. White circles indicate individual sequences that were included in both the small and the large subsamples, and dark circles indicate sequences that were added only in the large subsample. Large circles in red or blue indicate OTUs in the small and the large subsamples, respectively. (b) Rarefaction curves generated with dereplication clustering at five different depths. (c) PCoA based on the Bray-Curtis distance, comparing 60% subsamples with the full datasets using de-replication clustering. All of the subsamples were rarefied to 30,000 sequences per sample to be included in this analysis. [file 40168_2015_81_MOESM8_ESM.tiff]

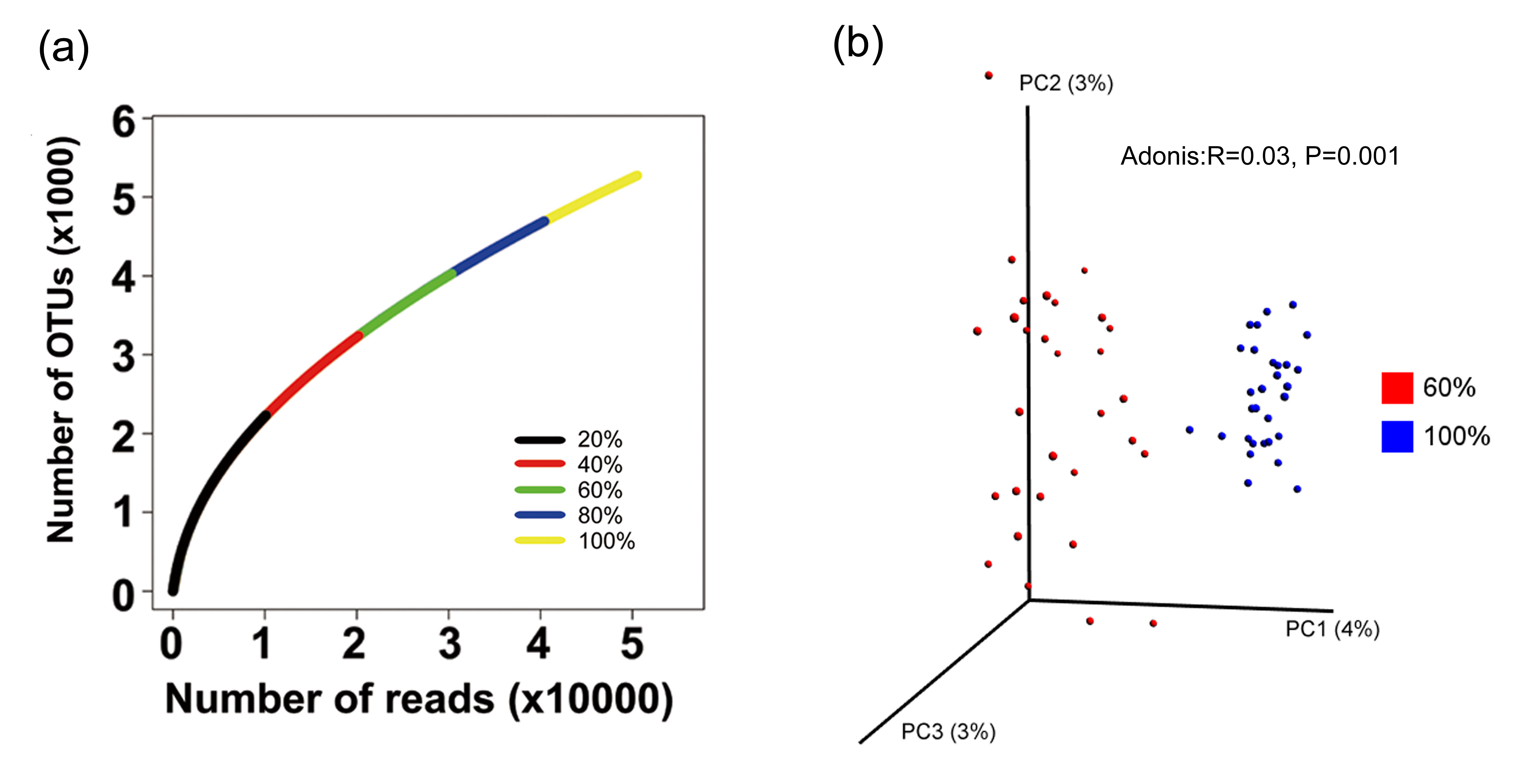

Supplement: Additional file 9: Figure S5. — Rarefaction curves and PCoA based on the Bray-Curtis distance. (a) Rarefaction curves generated with open-reference OTU clustering at five different depths. (b) PCoA based on the Bray-Curtis distance, comparing 60% subsamples with the full datasets using open-reference OTU clustering. All of the subsamples were rarefied to 30,000 sequences per sample to be included in this analysis. [file 40168_2015_81_MOESM9_ESM.tiff]

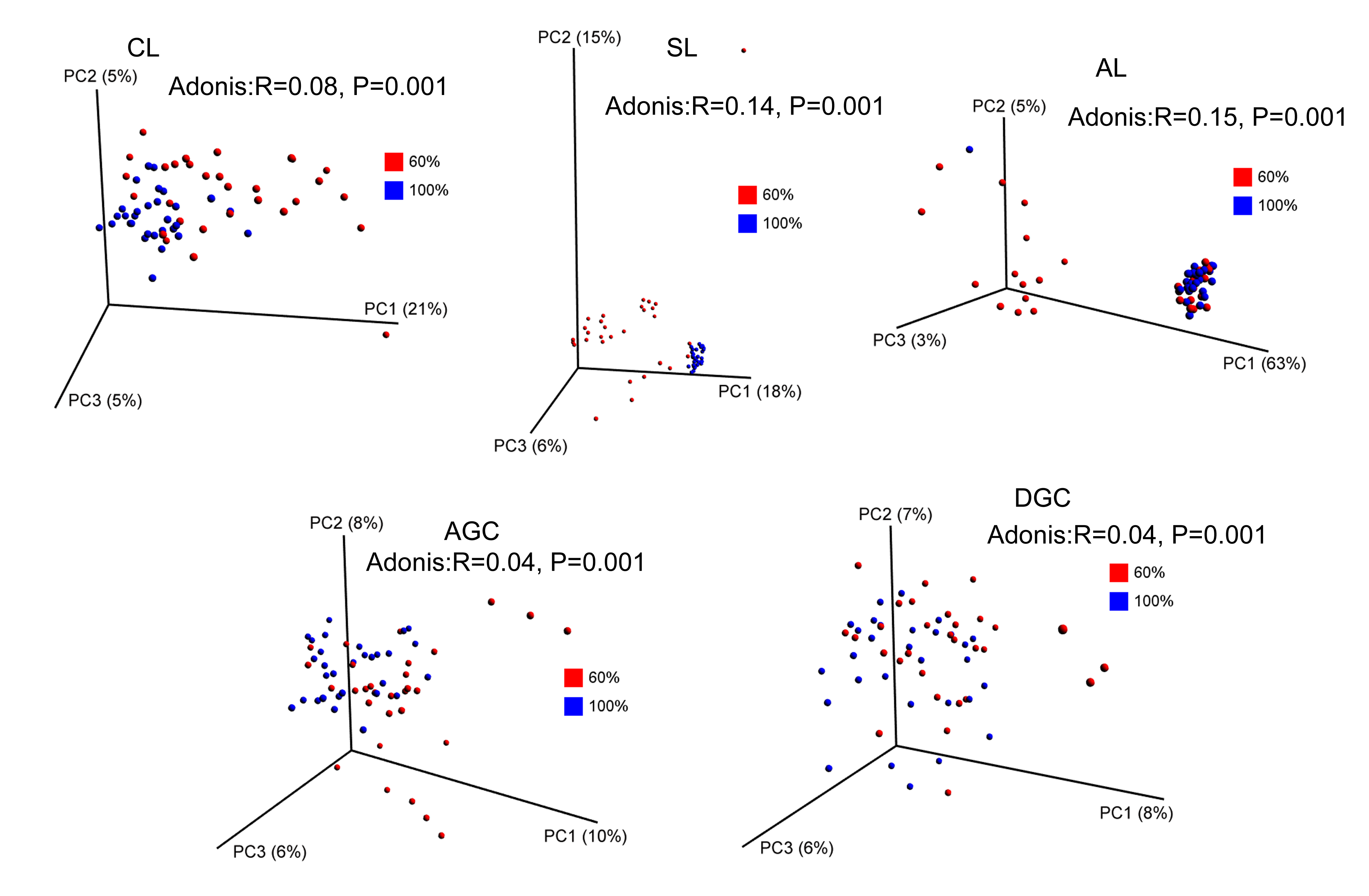

Supplement: Additional file 10: Figure S7. — PCoA with weighted UniFrac, comparing 60% subsamples with the full datasets using de novo clustering methods. All of the subsamples were rarefied to 30,000 sequences per sample to be included in this analysis. [file 40168_2015_81_MOESM10_ESM.tiff]
