# Supplementary material for: Stability of operational taxonomic units: an important but neglected property for analyzing microbial diversity
Source: Microbiome. 2015 May 20;3:20. doi: 10.1186/s40168-015-0081-x (PMC4438525; doi:10.1186/s40168-015-0081-x)
Supplement: Additional file 4: — Taxonomic composition from phylum to genus level, comparing 60% and full datasets using CL. All of the subsamples were rarefied to 30,000 sequences per sample (60% of the full dataset) to be included in this analysis. [file 40168_2015_81_MOESM4_ESM.zip › taxa_summary_plots/bar_charts.html]

Taxa Summaries


|  |  |
| --- | --- |
|  | |
| Taxonomy Summary. Current Level: | |
| View Figure (.pdf)  View Legend (.pdf) |  |
|  |


|  |
| --- |
| View Table (.txt) |

|  |  |  |  |  |  |
| --- | --- | --- | --- | --- | --- |
|  | | Total | | total | 30000 |
| Legend | Taxonomy | count | % | % | % |
|  | Archaea;Euryarchaeota | 0 | 0.0% | 0.0% | 0.0% |
|  | Archaea;Other | 0 | 0.0% | 0.0% | 0.0% |
|  | Bacteria;Acidobacteria | 0 | 5.0% | 5.1% | 5.0% |
|  | Bacteria;Actinobacteria | 0 | 4.5% | 4.4% | 4.5% |
|  | Bacteria;Bacteroidetes | 0 | 13.6% | 13.6% | 13.6% |
|  | Bacteria;Chlamydiae | 0 | 0.2% | 0.2% | 0.2% |
|  | Bacteria;Chloroflexi | 0 | 0.0% | 0.0% | 0.0% |
|  | Bacteria;Firmicutes | 0 | 0.7% | 0.7% | 0.7% |
|  | Bacteria;Gemmatimonadetes | 0 | 0.7% | 0.7% | 0.7% |
|  | Bacteria;Nitrospira | 0 | 0.0% | 0.0% | 0.0% |
|  | Bacteria;OP10 | 0 | 0.1% | 0.1% | 0.1% |
|  | Bacteria;Other | 1 | 30.4% | 30.5% | 30.4% |
|  | Bacteria;Planctomycetes | 0 | 0.0% | 0.0% | 0.0% |
|  | Bacteria;Proteobacteria | 1 | 43.0% | 43.0% | 43.0% |
|  | Bacteria;Spirochaetes | 0 | 0.0% | 0.0% | 0.0% |
|  | Bacteria;TM7 | 0 | 0.4% | 0.4% | 0.4% |
|  | Bacteria;Verrucomicrobia | 0 | 1.0% | 1.0% | 1.0% |
|  | Unclassified;Other | 0 | 0.3% | 0.3% | 0.3% |

|  |  |
| --- | --- |
|  | |
| Taxonomy Summary. Current Level: | |
| View Figure (.pdf)  View Legend (.pdf) |  |
|  |


|  |
| --- |
| View Table (.txt) |

|  |  |  |  |  |  |
| --- | --- | --- | --- | --- | --- |
|  | | Total | | total | 30000 |
| Legend | Taxonomy | count | % | % | % |
|  | Archaea;Euryarchaeota;Other | 0 | 0.0% | 0.0% | 0.0% |
|  | Archaea;Other;Other | 0 | 0.0% | 0.0% | 0.0% |
|  | Bacteria;Acidobacteria;Acidobacteria\_Gp1 | 0 | 0.2% | 0.2% | 0.2% |
|  | Bacteria;Acidobacteria;Acidobacteria\_Gp2 | 0 | 1.1% | 1.1% | 1.1% |
|  | Bacteria;Acidobacteria;Acidobacteria\_Gp22 | 0 | 0.1% | 0.1% | 0.1% |
|  | Bacteria;Acidobacteria;Acidobacteria\_Gp3 | 0 | 0.9% | 0.9% | 0.9% |
|  | Bacteria;Acidobacteria;Acidobacteria\_Gp4 | 0 | 0.1% | 0.1% | 0.1% |
|  | Bacteria;Acidobacteria;Acidobacteria\_Gp5 | 0 | 1.0% | 1.0% | 1.0% |
|  | Bacteria;Acidobacteria;Acidobacteria\_Gp6 | 0 | 0.1% | 0.1% | 0.0% |
|  | Bacteria;Acidobacteria;Acidobacteria\_Gp7 | 0 | 0.1% | 0.1% | 0.1% |
|  | Bacteria;Acidobacteria;Holophagae | 0 | 0.0% | 0.0% | 0.0% |
|  | Bacteria;Acidobacteria;Other | 0 | 1.4% | 1.4% | 1.4% |
|  | Bacteria;Actinobacteria;Actinobacteria | 0 | 4.5% | 4.4% | 4.5% |
|  | Bacteria;Bacteroidetes;Flavobacteria | 0 | 0.2% | 0.2% | 0.2% |
|  | Bacteria;Bacteroidetes;Other | 0 | 2.1% | 2.1% | 2.1% |
|  | Bacteria;Bacteroidetes;Sphingobacteria | 0 | 11.3% | 11.3% | 11.3% |
|  | Bacteria;Chlamydiae;Chlamydiae | 0 | 0.2% | 0.2% | 0.2% |
|  | Bacteria;Chloroflexi;Other | 0 | 0.0% | 0.0% | 0.0% |
|  | Bacteria;Firmicutes;Bacilli | 0 | 0.2% | 0.2% | 0.2% |
|  | Bacteria;Firmicutes;Clostridia | 0 | 0.1% | 0.1% | 0.1% |
|  | Bacteria;Firmicutes;Other | 0 | 0.5% | 0.5% | 0.5% |
|  | Bacteria;Gemmatimonadetes;Gemmatimonadetes | 0 | 0.7% | 0.7% | 0.7% |
|  | Bacteria;Nitrospira;Nitrospira | 0 | 0.0% | 0.0% | 0.0% |
|  | Bacteria;OP10;OP10\_genera\_incertae\_sedis | 0 | 0.1% | 0.1% | 0.1% |
|  | Bacteria;Other;Other | 1 | 30.4% | 30.5% | 30.4% |
|  | Bacteria;Planctomycetes;Planctomycetacia | 0 | 0.0% | 0.0% | 0.0% |
|  | Bacteria;Proteobacteria;Alphaproteobacteria | 0 | 10.8% | 10.8% | 10.8% |
|  | Bacteria;Proteobacteria;Betaproteobacteria | 0 | 10.0% | 10.0% | 10.1% |
|  | Bacteria;Proteobacteria;Deltaproteobacteria | 0 | 5.7% | 5.7% | 5.7% |
|  | Bacteria;Proteobacteria;Gammaproteobacteria | 0 | 3.0% | 3.0% | 3.0% |
|  | Bacteria;Proteobacteria;Other | 0 | 13.5% | 13.5% | 13.5% |
|  | Bacteria;Spirochaetes;Spirochaetes | 0 | 0.0% | 0.0% | 0.0% |
|  | Bacteria;TM7;TM7\_genera\_incertae\_sedis | 0 | 0.4% | 0.4% | 0.4% |
|  | Bacteria;Verrucomicrobia;Other | 0 | 0.1% | 0.1% | 0.1% |
|  | Bacteria;Verrucomicrobia;Spartobacteria | 0 | 0.0% | 0.0% | 0.0% |
|  | Bacteria;Verrucomicrobia;Subdivision3 | 0 | 0.9% | 0.9% | 0.9% |
|  | Bacteria;Verrucomicrobia;Subdivision5 | 0 | 0.0% | 0.0% | 0.0% |
|  | Unclassified;Other;Other | 0 | 0.3% | 0.3% | 0.3% |

|  |  |
| --- | --- |
|  | |
| Taxonomy Summary. Current Level: | |
| View Figure (.pdf)  View Legend (.pdf) |  |
|  |


|  |
| --- |
| View Table (.txt) |

|  |  |  |  |  |  |
| --- | --- | --- | --- | --- | --- |
|  | | Total | | total | 30000 |
| Legend | Taxonomy | count | % | % | % |
|  | Archaea;Euryarchaeota;Other;Other | 0 | 0.0% | 0.0% | 0.0% |
|  | Archaea;Other;Other;Other | 0 | 0.0% | 0.0% | 0.0% |
|  | Bacteria;Acidobacteria;Acidobacteria\_Gp1;Gp1 | 0 | 0.2% | 0.2% | 0.2% |
|  | Bacteria;Acidobacteria;Acidobacteria\_Gp2;Gp2 | 0 | 1.1% | 1.1% | 1.1% |
|  | Bacteria;Acidobacteria;Acidobacteria\_Gp22;Gp22 | 0 | 0.1% | 0.1% | 0.1% |
|  | Bacteria;Acidobacteria;Acidobacteria\_Gp3;Gp3 | 0 | 0.9% | 0.9% | 0.9% |
|  | Bacteria;Acidobacteria;Acidobacteria\_Gp4;Gp4 | 0 | 0.1% | 0.1% | 0.1% |
|  | Bacteria;Acidobacteria;Acidobacteria\_Gp5;Gp5 | 0 | 1.0% | 1.0% | 1.0% |
|  | Bacteria;Acidobacteria;Acidobacteria\_Gp6;Gp6 | 0 | 0.1% | 0.1% | 0.0% |
|  | Bacteria;Acidobacteria;Acidobacteria\_Gp7;Gp7 | 0 | 0.1% | 0.1% | 0.1% |
|  | Bacteria;Acidobacteria;Holophagae;Holophagales | 0 | 0.0% | 0.0% | 0.0% |
|  | Bacteria;Acidobacteria;Other;Other | 0 | 1.4% | 1.4% | 1.4% |
|  | Bacteria;Actinobacteria;Actinobacteria;Acidimicrobiales | 0 | 0.1% | 0.1% | 0.1% |
|  | Bacteria;Actinobacteria;Actinobacteria;Actinomycetales | 0 | 2.1% | 2.0% | 2.1% |
|  | Bacteria;Actinobacteria;Actinobacteria;Other | 0 | 2.1% | 2.1% | 2.1% |
|  | Bacteria;Actinobacteria;Actinobacteria;Solirubrobacterales | 0 | 0.2% | 0.2% | 0.2% |
|  | Bacteria;Bacteroidetes;Flavobacteria;Flavobacteriales | 0 | 0.2% | 0.2% | 0.2% |
|  | Bacteria;Bacteroidetes;Other;Other | 0 | 2.1% | 2.1% | 2.1% |
|  | Bacteria;Bacteroidetes;Sphingobacteria;Sphingobacteriales | 0 | 11.3% | 11.3% | 11.3% |
|  | Bacteria;Chlamydiae;Chlamydiae;Chlamydiales | 0 | 0.2% | 0.2% | 0.2% |
|  | Bacteria;Chloroflexi;Other;Other | 0 | 0.0% | 0.0% | 0.0% |
|  | Bacteria;Firmicutes;Bacilli;Bacillales | 0 | 0.1% | 0.1% | 0.1% |
|  | Bacteria;Firmicutes;Bacilli;Other | 0 | 0.1% | 0.0% | 0.1% |
|  | Bacteria;Firmicutes;Clostridia;Clostridiales | 0 | 0.0% | 0.0% | 0.0% |
|  | Bacteria;Firmicutes;Clostridia;Other | 0 | 0.1% | 0.1% | 0.1% |
|  | Bacteria;Firmicutes;Other;Other | 0 | 0.5% | 0.5% | 0.5% |
|  | Bacteria;Gemmatimonadetes;Gemmatimonadetes;Gemmatimonadales | 0 | 0.7% | 0.7% | 0.7% |
|  | Bacteria;Nitrospira;Nitrospira;Nitrospirales | 0 | 0.0% | 0.0% | 0.0% |
|  | Bacteria;OP10;OP10\_genera\_incertae\_sedis;Other | 0 | 0.1% | 0.1% | 0.1% |
|  | Bacteria;Other;Other;Other | 1 | 30.4% | 30.5% | 30.4% |
|  | Bacteria;Planctomycetes;Planctomycetacia;Planctomycetales | 0 | 0.0% | 0.0% | 0.0% |
|  | Bacteria;Proteobacteria;Alphaproteobacteria;Caulobacterales | 0 | 0.3% | 0.3% | 0.3% |
|  | Bacteria;Proteobacteria;Alphaproteobacteria;Other | 0 | 5.9% | 5.9% | 5.9% |
|  | Bacteria;Proteobacteria;Alphaproteobacteria;Rhizobiales | 0 | 4.1% | 4.1% | 4.1% |
|  | Bacteria;Proteobacteria;Alphaproteobacteria;Rhodospirillales | 0 | 0.2% | 0.2% | 0.2% |
|  | Bacteria;Proteobacteria;Alphaproteobacteria;Rickettsiales | 0 | 0.0% | 0.0% | 0.0% |
|  | Bacteria;Proteobacteria;Alphaproteobacteria;Sphingomonadales | 0 | 0.3% | 0.3% | 0.3% |
|  | Bacteria;Proteobacteria;Betaproteobacteria;Burkholderiales | 0 | 1.9% | 1.9% | 1.9% |
|  | Bacteria;Proteobacteria;Betaproteobacteria;Neisseriales | 0 | 0.0% | 0.0% | 0.0% |
|  | Bacteria;Proteobacteria;Betaproteobacteria;Nitrosomonadales | 0 | 0.0% | 0.0% | 0.0% |
|  | Bacteria;Proteobacteria;Betaproteobacteria;Other | 0 | 8.1% | 8.1% | 8.2% |
|  | Bacteria;Proteobacteria;Betaproteobacteria;Rhodocyclales | 0 | 0.0% | 0.0% | 0.0% |
|  | Bacteria;Proteobacteria;Deltaproteobacteria;Bdellovibrionales | 0 | 0.0% | 0.0% | 0.0% |
|  | Bacteria;Proteobacteria;Deltaproteobacteria;Desulfuromonadales | 0 | 0.3% | 0.3% | 0.3% |
|  | Bacteria;Proteobacteria;Deltaproteobacteria;Myxococcales | 0 | 4.8% | 4.8% | 4.8% |
|  | Bacteria;Proteobacteria;Deltaproteobacteria;Other | 0 | 0.6% | 0.6% | 0.6% |
|  | Bacteria;Proteobacteria;Gammaproteobacteria;Enterobacteriales | 0 | 0.0% | 0.0% | 0.0% |
|  | Bacteria;Proteobacteria;Gammaproteobacteria;Gammaproteobacteria\_incertae\_sedis | 0 | 0.0% | 0.0% | 0.0% |
|  | Bacteria;Proteobacteria;Gammaproteobacteria;Legionellales | 0 | 0.0% | 0.0% | 0.0% |
|  | Bacteria;Proteobacteria;Gammaproteobacteria;Other | 0 | 1.7% | 1.7% | 1.7% |
|  | Bacteria;Proteobacteria;Gammaproteobacteria;Pseudomonadales | 0 | 0.7% | 0.7% | 0.7% |
|  | Bacteria;Proteobacteria;Gammaproteobacteria;Xanthomonadales | 0 | 0.5% | 0.5% | 0.5% |
|  | Bacteria;Proteobacteria;Other;Other | 0 | 13.5% | 13.5% | 13.5% |
|  | Bacteria;Spirochaetes;Spirochaetes;Spirochaetales | 0 | 0.0% | 0.0% | 0.0% |
|  | Bacteria;TM7;TM7\_genera\_incertae\_sedis;Other | 0 | 0.4% | 0.4% | 0.4% |
|  | Bacteria;Verrucomicrobia;Other;Other | 0 | 0.1% | 0.1% | 0.1% |
|  | Bacteria;Verrucomicrobia;Spartobacteria;Other | 0 | 0.0% | 0.0% | 0.0% |
|  | Bacteria;Verrucomicrobia;Spartobacteria;Spartobacteria\_genera\_incertae\_sedis | 0 | 0.0% | 0.0% | 0.0% |
|  | Bacteria;Verrucomicrobia;Subdivision3;Subdivision3\_genera\_incertae\_sedis | 0 | 0.9% | 0.9% | 0.9% |
|  | Bacteria;Verrucomicrobia;Subdivision5;Subdivision5\_genera\_incertae\_sedis | 0 | 0.0% | 0.0% | 0.0% |
|  | Unclassified;Other;Other;Other | 0 | 0.3% | 0.3% | 0.3% |

|  |  |
| --- | --- |
|  | |
| Taxonomy Summary. Current Level: | |
| View Figure (.pdf)  View Legend (.pdf) |  |
|  |


|  |
| --- |
| View Table (.txt) |

|  |  |  |  |  |  |
| --- | --- | --- | --- | --- | --- |
|  | | Total | | total | 30000 |
| Legend | Taxonomy | count | % | % | % |
|  | Archaea;Euryarchaeota;Other;Other;Other | 0 | 0.0% | 0.0% | 0.0% |
|  | Archaea;Other;Other;Other;Other | 0 | 0.0% | 0.0% | 0.0% |
|  | Bacteria;Acidobacteria;Acidobacteria\_Gp1;Gp1;Other | 0 | 0.2% | 0.2% | 0.2% |
|  | Bacteria;Acidobacteria;Acidobacteria\_Gp2;Gp2;Other | 0 | 1.1% | 1.1% | 1.1% |
|  | Bacteria;Acidobacteria;Acidobacteria\_Gp22;Gp22;Other | 0 | 0.1% | 0.1% | 0.1% |
|  | Bacteria;Acidobacteria;Acidobacteria\_Gp3;Gp3;Other | 0 | 0.9% | 0.9% | 0.9% |
|  | Bacteria;Acidobacteria;Acidobacteria\_Gp4;Gp4;Other | 0 | 0.1% | 0.1% | 0.1% |
|  | Bacteria;Acidobacteria;Acidobacteria\_Gp5;Gp5;Other | 0 | 1.0% | 1.0% | 1.0% |
|  | Bacteria;Acidobacteria;Acidobacteria\_Gp6;Gp6;Other | 0 | 0.1% | 0.1% | 0.0% |
|  | Bacteria;Acidobacteria;Acidobacteria\_Gp7;Gp7;Other | 0 | 0.1% | 0.1% | 0.1% |
|  | Bacteria;Acidobacteria;Holophagae;Holophagales;Holophagaceae | 0 | 0.0% | 0.0% | 0.0% |
|  | Bacteria;Acidobacteria;Other;Other;Other | 0 | 1.4% | 1.4% | 1.4% |
|  | Bacteria;Actinobacteria;Actinobacteria;Acidimicrobiales;Iamiaceae | 0 | 0.1% | 0.1% | 0.1% |
|  | Bacteria;Actinobacteria;Actinobacteria;Acidimicrobiales;Other | 0 | 0.0% | 0.0% | 0.0% |
|  | Bacteria;Actinobacteria;Actinobacteria;Actinomycetales;Geodermatophilaceae | 0 | 0.0% | 0.0% | 0.0% |
|  | Bacteria;Actinobacteria;Actinobacteria;Actinomycetales;Microbacteriaceae | 0 | 0.1% | 0.2% | 0.1% |
|  | Bacteria;Actinobacteria;Actinobacteria;Actinomycetales;Micrococcaceae | 0 | 0.0% | 0.0% | 0.0% |
|  | Bacteria;Actinobacteria;Actinobacteria;Actinomycetales;Micromonosporaceae | 0 | 0.2% | 0.2% | 0.2% |
|  | Bacteria;Actinobacteria;Actinobacteria;Actinomycetales;Nocardiaceae | 0 | 0.4% | 0.4% | 0.5% |
|  | Bacteria;Actinobacteria;Actinobacteria;Actinomycetales;Nocardioidaceae | 0 | 0.2% | 0.2% | 0.2% |
|  | Bacteria;Actinobacteria;Actinobacteria;Actinomycetales;Other | 0 | 0.9% | 0.9% | 0.9% |
|  | Bacteria;Actinobacteria;Actinobacteria;Actinomycetales;Pseudonocardiaceae | 0 | 0.0% | 0.0% | 0.0% |
|  | Bacteria;Actinobacteria;Actinobacteria;Actinomycetales;Streptomycetaceae | 0 | 0.1% | 0.1% | 0.1% |
|  | Bacteria;Actinobacteria;Actinobacteria;Actinomycetales;Streptosporangiaceae | 0 | 0.0% | 0.0% | 0.0% |
|  | Bacteria;Actinobacteria;Actinobacteria;Actinomycetales;Thermomonosporaceae | 0 | 0.0% | 0.0% | 0.0% |
|  | Bacteria;Actinobacteria;Actinobacteria;Other;Other | 0 | 2.1% | 2.1% | 2.1% |
|  | Bacteria;Actinobacteria;Actinobacteria;Solirubrobacterales;Other | 0 | 0.1% | 0.1% | 0.1% |
|  | Bacteria;Actinobacteria;Actinobacteria;Solirubrobacterales;Patulibacteraceae | 0 | 0.1% | 0.1% | 0.1% |
|  | Bacteria;Actinobacteria;Actinobacteria;Solirubrobacterales;Solirubrobacteraceae | 0 | 0.1% | 0.1% | 0.1% |
|  | Bacteria;Bacteroidetes;Flavobacteria;Flavobacteriales;Cryomorphaceae | 0 | 0.0% | 0.0% | 0.0% |
|  | Bacteria;Bacteroidetes;Flavobacteria;Flavobacteriales;Flavobacteriaceae | 0 | 0.2% | 0.2% | 0.2% |
|  | Bacteria;Bacteroidetes;Flavobacteria;Flavobacteriales;Other | 0 | 0.0% | 0.0% | 0.0% |
|  | Bacteria;Bacteroidetes;Other;Other;Other | 0 | 2.1% | 2.1% | 2.1% |
|  | Bacteria;Bacteroidetes;Sphingobacteria;Sphingobacteriales;Chitinophagaceae | 0 | 8.5% | 8.5% | 8.5% |
|  | Bacteria;Bacteroidetes;Sphingobacteria;Sphingobacteriales;Cytophagaceae | 0 | 0.0% | 0.0% | 0.0% |
|  | Bacteria;Bacteroidetes;Sphingobacteria;Sphingobacteriales;Other | 0 | 1.8% | 1.8% | 1.8% |
|  | Bacteria;Bacteroidetes;Sphingobacteria;Sphingobacteriales;Sphingobacteriaceae | 0 | 1.0% | 1.0% | 1.0% |
|  | Bacteria;Chlamydiae;Chlamydiae;Chlamydiales;Other | 0 | 0.1% | 0.1% | 0.2% |
|  | Bacteria;Chlamydiae;Chlamydiae;Chlamydiales;Parachlamydiaceae | 0 | 0.0% | 0.0% | 0.0% |
|  | Bacteria;Chloroflexi;Other;Other;Other | 0 | 0.0% | 0.0% | 0.0% |
|  | Bacteria;Firmicutes;Bacilli;Bacillales;Bacillaceae | 0 | 0.0% | 0.0% | 0.0% |
|  | Bacteria;Firmicutes;Bacilli;Bacillales;Other | 0 | 0.1% | 0.1% | 0.1% |
|  | Bacteria;Firmicutes;Bacilli;Bacillales;Paenibacillaceae | 0 | 0.0% | 0.0% | 0.0% |
|  | Bacteria;Firmicutes;Bacilli;Bacillales;Thermoactinomycetaceae | 0 | 0.0% | 0.0% | 0.0% |
|  | Bacteria;Firmicutes;Bacilli;Other;Other | 0 | 0.1% | 0.0% | 0.1% |
|  | Bacteria;Firmicutes;Clostridia;Clostridiales;Clostridiaceae | 0 | 0.0% | 0.0% | 0.0% |
|  | Bacteria;Firmicutes;Clostridia;Other;Other | 0 | 0.1% | 0.1% | 0.1% |
|  | Bacteria;Firmicutes;Other;Other;Other | 0 | 0.5% | 0.5% | 0.5% |
|  | Bacteria;Gemmatimonadetes;Gemmatimonadetes;Gemmatimonadales;Gemmatimonadaceae | 0 | 0.7% | 0.7% | 0.7% |
|  | Bacteria;Nitrospira;Nitrospira;Nitrospirales;Nitrospiraceae | 0 | 0.0% | 0.0% | 0.0% |
|  | Bacteria;OP10;OP10\_genera\_incertae\_sedis;Other;Other | 0 | 0.1% | 0.1% | 0.1% |
|  | Bacteria;Other;Other;Other;Other | 1 | 30.4% | 30.5% | 30.4% |
|  | Bacteria;Planctomycetes;Planctomycetacia;Planctomycetales;Planctomycetaceae | 0 | 0.0% | 0.0% | 0.0% |
|  | Bacteria;Proteobacteria;Alphaproteobacteria;Caulobacterales;Caulobacteraceae | 0 | 0.3% | 0.3% | 0.3% |
|  | Bacteria;Proteobacteria;Alphaproteobacteria;Other;Other | 0 | 5.9% | 5.9% | 5.9% |
|  | Bacteria;Proteobacteria;Alphaproteobacteria;Rhizobiales;Bradyrhizobiaceae | 0 | 3.0% | 3.0% | 3.0% |
|  | Bacteria;Proteobacteria;Alphaproteobacteria;Rhizobiales;Brucellaceae | 0 | 0.1% | 0.1% | 0.1% |
|  | Bacteria;Proteobacteria;Alphaproteobacteria;Rhizobiales;Hyphomicrobiaceae | 0 | 0.0% | 0.0% | 0.0% |
|  | Bacteria;Proteobacteria;Alphaproteobacteria;Rhizobiales;Methylobacteriaceae | 0 | 0.0% | 0.0% | 0.0% |
|  | Bacteria;Proteobacteria;Alphaproteobacteria;Rhizobiales;Methylocystaceae | 0 | 0.0% | 0.0% | 0.0% |
|  | Bacteria;Proteobacteria;Alphaproteobacteria;Rhizobiales;Other | 0 | 0.9% | 0.9% | 0.9% |
|  | Bacteria;Proteobacteria;Alphaproteobacteria;Rhizobiales;Rhizobiaceae | 0 | 0.1% | 0.1% | 0.1% |
|  | Bacteria;Proteobacteria;Alphaproteobacteria;Rhizobiales;Xanthobacteraceae | 0 | 0.0% | 0.0% | 0.0% |
|  | Bacteria;Proteobacteria;Alphaproteobacteria;Rhodospirillales;Acetobacteraceae | 0 | 0.1% | 0.1% | 0.1% |
|  | Bacteria;Proteobacteria;Alphaproteobacteria;Rhodospirillales;Other | 0 | 0.1% | 0.1% | 0.1% |
|  | Bacteria;Proteobacteria;Alphaproteobacteria;Rickettsiales;Rickettsiaceae | 0 | 0.0% | 0.0% | 0.0% |
|  | Bacteria;Proteobacteria;Alphaproteobacteria;Sphingomonadales;Erythrobacteraceae | 0 | 0.0% | 0.0% | 0.0% |
|  | Bacteria;Proteobacteria;Alphaproteobacteria;Sphingomonadales;Other | 0 | 0.2% | 0.2% | 0.2% |
|  | Bacteria;Proteobacteria;Alphaproteobacteria;Sphingomonadales;Sphingomonadaceae | 0 | 0.1% | 0.1% | 0.1% |
|  | Bacteria;Proteobacteria;Betaproteobacteria;Burkholderiales;Alcaligenaceae | 0 | 0.0% | 0.0% | 0.0% |
|  | Bacteria;Proteobacteria;Betaproteobacteria;Burkholderiales;Burkholderiaceae | 0 | 0.3% | 0.3% | 0.3% |
|  | Bacteria;Proteobacteria;Betaproteobacteria;Burkholderiales;Comamonadaceae | 0 | 0.1% | 0.1% | 0.0% |
|  | Bacteria;Proteobacteria;Betaproteobacteria;Burkholderiales;Other | 0 | 1.1% | 1.1% | 1.1% |
|  | Bacteria;Proteobacteria;Betaproteobacteria;Burkholderiales;Oxalobacteraceae | 0 | 0.4% | 0.4% | 0.4% |
|  | Bacteria;Proteobacteria;Betaproteobacteria;Neisseriales;Neisseriaceae | 0 | 0.0% | 0.0% | 0.0% |
|  | Bacteria;Proteobacteria;Betaproteobacteria;Nitrosomonadales;Nitrosomonadaceae | 0 | 0.0% | 0.0% | 0.0% |
|  | Bacteria;Proteobacteria;Betaproteobacteria;Other;Other | 0 | 8.1% | 8.1% | 8.2% |
|  | Bacteria;Proteobacteria;Betaproteobacteria;Rhodocyclales;Rhodocyclaceae | 0 | 0.0% | 0.0% | 0.0% |
|  | Bacteria;Proteobacteria;Deltaproteobacteria;Bdellovibrionales;Bacteriovoracaceae | 0 | 0.0% | 0.0% | 0.0% |
|  | Bacteria;Proteobacteria;Deltaproteobacteria;Desulfuromonadales;Geobacteraceae | 0 | 0.3% | 0.3% | 0.3% |
|  | Bacteria;Proteobacteria;Deltaproteobacteria;Desulfuromonadales;Other | 0 | 0.0% | 0.0% | 0.0% |
|  | Bacteria;Proteobacteria;Deltaproteobacteria;Myxococcales;Cystobacteraceae | 0 | 0.4% | 0.4% | 0.4% |
|  | Bacteria;Proteobacteria;Deltaproteobacteria;Myxococcales;Haliangiaceae | 0 | 0.6% | 0.6% | 0.6% |
|  | Bacteria;Proteobacteria;Deltaproteobacteria;Myxococcales;Nannocystaceae | 0 | 0.0% | 0.0% | 0.0% |
|  | Bacteria;Proteobacteria;Deltaproteobacteria;Myxococcales;Other | 0 | 3.4% | 3.4% | 3.4% |
|  | Bacteria;Proteobacteria;Deltaproteobacteria;Myxococcales;Polyangiaceae | 0 | 0.4% | 0.4% | 0.3% |
|  | Bacteria;Proteobacteria;Deltaproteobacteria;Other;Other | 0 | 0.6% | 0.6% | 0.6% |
|  | Bacteria;Proteobacteria;Gammaproteobacteria;Enterobacteriales;Enterobacteriaceae | 0 | 0.0% | 0.0% | 0.0% |
|  | Bacteria;Proteobacteria;Gammaproteobacteria;Gammaproteobacteria\_incertae\_sedis;Solimonas | 0 | 0.0% | 0.0% | 0.0% |
|  | Bacteria;Proteobacteria;Gammaproteobacteria;Legionellales;Coxiellaceae | 0 | 0.0% | 0.0% | 0.0% |
|  | Bacteria;Proteobacteria;Gammaproteobacteria;Legionellales;Legionellaceae | 0 | 0.0% | 0.0% | 0.0% |
|  | Bacteria;Proteobacteria;Gammaproteobacteria;Other;Other | 0 | 1.7% | 1.7% | 1.7% |
|  | Bacteria;Proteobacteria;Gammaproteobacteria;Pseudomonadales;Pseudomonadaceae | 0 | 0.7% | 0.7% | 0.7% |
|  | Bacteria;Proteobacteria;Gammaproteobacteria;Xanthomonadales;Other | 0 | 0.0% | 0.0% | 0.0% |
|  | Bacteria;Proteobacteria;Gammaproteobacteria;Xanthomonadales;Sinobacteraceae | 0 | 0.0% | 0.0% | 0.0% |
|  | Bacteria;Proteobacteria;Gammaproteobacteria;Xanthomonadales;Xanthomonadaceae | 0 | 0.5% | 0.5% | 0.5% |
|  | Bacteria;Proteobacteria;Other;Other;Other | 0 | 13.5% | 13.5% | 13.5% |
|  | Bacteria;Spirochaetes;Spirochaetes;Spirochaetales;Leptospiraceae | 0 | 0.0% | 0.0% | 0.0% |
|  | Bacteria;TM7;TM7\_genera\_incertae\_sedis;Other;Other | 0 | 0.4% | 0.4% | 0.4% |
|  | Bacteria;Verrucomicrobia;Other;Other;Other | 0 | 0.1% | 0.1% | 0.1% |
|  | Bacteria;Verrucomicrobia;Spartobacteria;Other;Other | 0 | 0.0% | 0.0% | 0.0% |
|  | Bacteria;Verrucomicrobia;Spartobacteria;Spartobacteria\_genera\_incertae\_sedis;Other | 0 | 0.0% | 0.0% | 0.0% |
|  | Bacteria;Verrucomicrobia;Subdivision3;Subdivision3\_genera\_incertae\_sedis;Other | 0 | 0.9% | 0.9% | 0.9% |
|  | Bacteria;Verrucomicrobia;Subdivision5;Subdivision5\_genera\_incertae\_sedis;Other | 0 | 0.0% | 0.0% | 0.0% |
|  | Unclassified;Other;Other;Other;Other | 0 | 0.3% | 0.3% | 0.3% |

|  |  |
| --- | --- |
|  | |
| Taxonomy Summary. Current Level: | |
| View Figure (.pdf)  View Legend (.pdf) |  |
|  |


|  |
| --- |
| View Table (.txt) |

|  |  |  |  |  |  |
| --- | --- | --- | --- | --- | --- |
|  | | Total | | total | 30000 |
| Legend | Taxonomy | count | % | % | % |
|  | Archaea;Euryarchaeota;Other;Other;Other;Other | 0 | 0.0% | 0.0% | 0.0% |
|  | Archaea;Other;Other;Other;Other;Other | 0 | 0.0% | 0.0% | 0.0% |
|  | Bacteria;Acidobacteria;Acidobacteria\_Gp1;Gp1;Other;Other | 0 | 0.2% | 0.2% | 0.2% |
|  | Bacteria;Acidobacteria;Acidobacteria\_Gp2;Gp2;Other;Other | 0 | 1.1% | 1.1% | 1.1% |
|  | Bacteria;Acidobacteria;Acidobacteria\_Gp22;Gp22;Other;Other | 0 | 0.1% | 0.1% | 0.1% |
|  | Bacteria;Acidobacteria;Acidobacteria\_Gp3;Gp3;Other;Other | 0 | 0.9% | 0.9% | 0.9% |
|  | Bacteria;Acidobacteria;Acidobacteria\_Gp4;Gp4;Other;Other | 0 | 0.1% | 0.1% | 0.1% |
|  | Bacteria;Acidobacteria;Acidobacteria\_Gp5;Gp5;Other;Other | 0 | 1.0% | 1.0% | 1.0% |
|  | Bacteria;Acidobacteria;Acidobacteria\_Gp6;Gp6;Other;Other | 0 | 0.1% | 0.1% | 0.0% |
|  | Bacteria;Acidobacteria;Acidobacteria\_Gp7;Gp7;Other;Other | 0 | 0.1% | 0.1% | 0.1% |
|  | Bacteria;Acidobacteria;Holophagae;Holophagales;Holophagaceae;Other | 0 | 0.0% | 0.0% | 0.0% |
|  | Bacteria;Acidobacteria;Other;Other;Other;Other | 0 | 1.4% | 1.4% | 1.4% |
|  | Bacteria;Actinobacteria;Actinobacteria;Acidimicrobiales;Iamiaceae;Iamia | 0 | 0.1% | 0.1% | 0.1% |
|  | Bacteria;Actinobacteria;Actinobacteria;Acidimicrobiales;Other;Other | 0 | 0.0% | 0.0% | 0.0% |
|  | Bacteria;Actinobacteria;Actinobacteria;Actinomycetales;Geodermatophilaceae;Other | 0 | 0.0% | 0.0% | 0.0% |
|  | Bacteria;Actinobacteria;Actinobacteria;Actinomycetales;Microbacteriaceae;Other | 0 | 0.1% | 0.2% | 0.1% |
|  | Bacteria;Actinobacteria;Actinobacteria;Actinomycetales;Micrococcaceae;Other | 0 | 0.0% | 0.0% | 0.0% |
|  | Bacteria;Actinobacteria;Actinobacteria;Actinomycetales;Micromonosporaceae;Actinoplanes | 0 | 0.0% | 0.0% | 0.0% |
|  | Bacteria;Actinobacteria;Actinobacteria;Actinomycetales;Micromonosporaceae;Other | 0 | 0.2% | 0.2% | 0.2% |
|  | Bacteria;Actinobacteria;Actinobacteria;Actinomycetales;Nocardiaceae;Other | 0 | 0.4% | 0.4% | 0.5% |
|  | Bacteria;Actinobacteria;Actinobacteria;Actinomycetales;Nocardioidaceae;Aeromicrobium | 0 | 0.1% | 0.1% | 0.1% |
|  | Bacteria;Actinobacteria;Actinobacteria;Actinomycetales;Nocardioidaceae;Kribbella | 0 | 0.1% | 0.1% | 0.1% |
|  | Bacteria;Actinobacteria;Actinobacteria;Actinomycetales;Nocardioidaceae;Other | 0 | 0.0% | 0.0% | 0.0% |
|  | Bacteria;Actinobacteria;Actinobacteria;Actinomycetales;Other;Other | 0 | 0.9% | 0.9% | 0.9% |
|  | Bacteria;Actinobacteria;Actinobacteria;Actinomycetales;Pseudonocardiaceae;Amycolatopsis | 0 | 0.0% | 0.0% | 0.0% |
|  | Bacteria;Actinobacteria;Actinobacteria;Actinomycetales;Pseudonocardiaceae;Other | 0 | 0.0% | 0.0% | 0.0% |
|  | Bacteria;Actinobacteria;Actinobacteria;Actinomycetales;Pseudonocardiaceae;Pseudonocardia | 0 | 0.0% | 0.0% | 0.0% |
|  | Bacteria;Actinobacteria;Actinobacteria;Actinomycetales;Streptomycetaceae;Kitasatospora | 0 | 0.0% | 0.0% | 0.0% |
|  | Bacteria;Actinobacteria;Actinobacteria;Actinomycetales;Streptomycetaceae;Other | 0 | 0.0% | 0.0% | 0.0% |
|  | Bacteria;Actinobacteria;Actinobacteria;Actinomycetales;Streptomycetaceae;Streptacidiphilus | 0 | 0.0% | 0.0% | 0.0% |
|  | Bacteria;Actinobacteria;Actinobacteria;Actinomycetales;Streptomycetaceae;Streptomyces | 0 | 0.1% | 0.1% | 0.1% |
|  | Bacteria;Actinobacteria;Actinobacteria;Actinomycetales;Streptosporangiaceae;Other | 0 | 0.0% | 0.0% | 0.0% |
|  | Bacteria;Actinobacteria;Actinobacteria;Actinomycetales;Thermomonosporaceae;Actinocorallia | 0 | 0.0% | 0.0% | 0.0% |
|  | Bacteria;Actinobacteria;Actinobacteria;Actinomycetales;Thermomonosporaceae;Other | 0 | 0.0% | 0.0% | 0.0% |
|  | Bacteria;Actinobacteria;Actinobacteria;Other;Other;Other | 0 | 2.1% | 2.1% | 2.1% |
|  | Bacteria;Actinobacteria;Actinobacteria;Solirubrobacterales;Other;Other | 0 | 0.1% | 0.1% | 0.1% |
|  | Bacteria;Actinobacteria;Actinobacteria;Solirubrobacterales;Patulibacteraceae;Patulibacter | 0 | 0.1% | 0.1% | 0.1% |
|  | Bacteria;Actinobacteria;Actinobacteria;Solirubrobacterales;Solirubrobacteraceae;Solirubrobacter | 0 | 0.1% | 0.1% | 0.1% |
|  | Bacteria;Bacteroidetes;Flavobacteria;Flavobacteriales;Cryomorphaceae;Other | 0 | 0.0% | 0.0% | 0.0% |
|  | Bacteria;Bacteroidetes;Flavobacteria;Flavobacteriales;Flavobacteriaceae;Flavobacterium | 0 | 0.1% | 0.1% | 0.1% |
|  | Bacteria;Bacteroidetes;Flavobacteria;Flavobacteriales;Flavobacteriaceae;Other | 0 | 0.1% | 0.1% | 0.1% |
|  | Bacteria;Bacteroidetes;Flavobacteria;Flavobacteriales;Other;Other | 0 | 0.0% | 0.0% | 0.0% |
|  | Bacteria;Bacteroidetes;Other;Other;Other;Other | 0 | 2.1% | 2.1% | 2.1% |
|  | Bacteria;Bacteroidetes;Sphingobacteria;Sphingobacteriales;Chitinophagaceae;Chitinophaga | 0 | 0.0% | 0.0% | 0.0% |
|  | Bacteria;Bacteroidetes;Sphingobacteria;Sphingobacteriales;Chitinophagaceae;Ferruginibacter | 0 | 1.3% | 1.2% | 1.3% |
|  | Bacteria;Bacteroidetes;Sphingobacteria;Sphingobacteriales;Chitinophagaceae;Filimonas | 0 | 0.0% | 0.0% | 0.0% |
|  | Bacteria;Bacteroidetes;Sphingobacteria;Sphingobacteriales;Chitinophagaceae;Flavisolibacter | 0 | 0.0% | 0.0% | 0.0% |
|  | Bacteria;Bacteroidetes;Sphingobacteria;Sphingobacteriales;Chitinophagaceae;Niastella | 0 | 0.6% | 0.6% | 0.6% |
|  | Bacteria;Bacteroidetes;Sphingobacteria;Sphingobacteriales;Chitinophagaceae;Other | 0 | 6.6% | 6.6% | 6.6% |
|  | Bacteria;Bacteroidetes;Sphingobacteria;Sphingobacteriales;Cytophagaceae;Cytophaga | 0 | 0.0% | 0.0% | 0.0% |
|  | Bacteria;Bacteroidetes;Sphingobacteria;Sphingobacteriales;Cytophagaceae;Dyadobacter | 0 | 0.0% | 0.0% | 0.0% |
|  | Bacteria;Bacteroidetes;Sphingobacteria;Sphingobacteriales;Cytophagaceae;Sporocytophaga | 0 | 0.0% | 0.0% | 0.0% |
|  | Bacteria;Bacteroidetes;Sphingobacteria;Sphingobacteriales;Other;Other | 0 | 1.8% | 1.8% | 1.8% |
|  | Bacteria;Bacteroidetes;Sphingobacteria;Sphingobacteriales;Sphingobacteriaceae;Mucilaginibacter | 0 | 1.0% | 0.9% | 1.0% |
|  | Bacteria;Bacteroidetes;Sphingobacteria;Sphingobacteriales;Sphingobacteriaceae;Other | 0 | 0.0% | 0.0% | 0.0% |
|  | Bacteria;Bacteroidetes;Sphingobacteria;Sphingobacteriales;Sphingobacteriaceae;Pedobacter | 0 | 0.0% | 0.0% | 0.0% |
|  | Bacteria;Chlamydiae;Chlamydiae;Chlamydiales;Other;Other | 0 | 0.1% | 0.1% | 0.2% |
|  | Bacteria;Chlamydiae;Chlamydiae;Chlamydiales;Parachlamydiaceae;Neochlamydia | 0 | 0.0% | 0.0% | 0.0% |
|  | Bacteria;Chlamydiae;Chlamydiae;Chlamydiales;Parachlamydiaceae;Other | 0 | 0.0% | 0.0% | 0.0% |
|  | Bacteria;Chlamydiae;Chlamydiae;Chlamydiales;Parachlamydiaceae;Parachlamydia | 0 | 0.0% | 0.0% | 0.0% |
|  | Bacteria;Chloroflexi;Other;Other;Other;Other | 0 | 0.0% | 0.0% | 0.0% |
|  | Bacteria;Firmicutes;Bacilli;Bacillales;Bacillaceae;Other | 0 | 0.0% | 0.0% | 0.0% |
|  | Bacteria;Firmicutes;Bacilli;Bacillales;Other;Other | 0 | 0.1% | 0.1% | 0.1% |
|  | Bacteria;Firmicutes;Bacilli;Bacillales;Paenibacillaceae;Other | 0 | 0.0% | 0.0% | 0.0% |
|  | Bacteria;Firmicutes;Bacilli;Bacillales;Paenibacillaceae;Paenibacillus | 0 | 0.0% | 0.0% | 0.0% |
|  | Bacteria;Firmicutes;Bacilli;Bacillales;Thermoactinomycetaceae;Other | 0 | 0.0% | 0.0% | 0.0% |
|  | Bacteria;Firmicutes;Bacilli;Other;Other;Other | 0 | 0.1% | 0.0% | 0.1% |
|  | Bacteria;Firmicutes;Clostridia;Clostridiales;Clostridiaceae;Clostridium | 0 | 0.0% | 0.0% | 0.0% |
|  | Bacteria;Firmicutes;Clostridia;Other;Other;Other | 0 | 0.1% | 0.1% | 0.1% |
|  | Bacteria;Firmicutes;Other;Other;Other;Other | 0 | 0.5% | 0.5% | 0.5% |
|  | Bacteria;Gemmatimonadetes;Gemmatimonadetes;Gemmatimonadales;Gemmatimonadaceae;Gemmatimonas | 0 | 0.7% | 0.7% | 0.7% |
|  | Bacteria;Nitrospira;Nitrospira;Nitrospirales;Nitrospiraceae;Nitrospira | 0 | 0.0% | 0.0% | 0.0% |
|  | Bacteria;OP10;OP10\_genera\_incertae\_sedis;Other;Other;Other | 0 | 0.1% | 0.1% | 0.1% |
|  | Bacteria;Other;Other;Other;Other;Other | 1 | 30.4% | 30.5% | 30.4% |
|  | Bacteria;Planctomycetes;Planctomycetacia;Planctomycetales;Planctomycetaceae;Other | 0 | 0.0% | 0.0% | 0.0% |
|  | Bacteria;Proteobacteria;Alphaproteobacteria;Caulobacterales;Caulobacteraceae;Phenylobacterium | 0 | 0.3% | 0.3% | 0.3% |
|  | Bacteria;Proteobacteria;Alphaproteobacteria;Other;Other;Other | 0 | 5.9% | 5.9% | 5.9% |
|  | Bacteria;Proteobacteria;Alphaproteobacteria;Rhizobiales;Bradyrhizobiaceae;Bradyrhizobium | 0 | 1.9% | 1.9% | 1.9% |
|  | Bacteria;Proteobacteria;Alphaproteobacteria;Rhizobiales;Bradyrhizobiaceae;Nitrobacter | 0 | 0.9% | 0.9% | 0.9% |
|  | Bacteria;Proteobacteria;Alphaproteobacteria;Rhizobiales;Bradyrhizobiaceae;Other | 0 | 0.2% | 0.2% | 0.2% |
|  | Bacteria;Proteobacteria;Alphaproteobacteria;Rhizobiales;Brucellaceae;Brucella | 0 | 0.1% | 0.1% | 0.1% |
|  | Bacteria;Proteobacteria;Alphaproteobacteria;Rhizobiales;Brucellaceae;Other | 0 | 0.0% | 0.0% | 0.0% |
|  | Bacteria;Proteobacteria;Alphaproteobacteria;Rhizobiales;Hyphomicrobiaceae;Devosia | 0 | 0.0% | 0.0% | 0.0% |
|  | Bacteria;Proteobacteria;Alphaproteobacteria;Rhizobiales;Hyphomicrobiaceae;Hyphomicrobium | 0 | 0.0% | 0.0% | 0.0% |
|  | Bacteria;Proteobacteria;Alphaproteobacteria;Rhizobiales;Methylobacteriaceae;Methylobacterium | 0 | 0.0% | 0.0% | 0.0% |
|  | Bacteria;Proteobacteria;Alphaproteobacteria;Rhizobiales;Methylocystaceae;Other | 0 | 0.0% | 0.0% | 0.0% |
|  | Bacteria;Proteobacteria;Alphaproteobacteria;Rhizobiales;Other;Other | 0 | 0.9% | 0.9% | 0.9% |
|  | Bacteria;Proteobacteria;Alphaproteobacteria;Rhizobiales;Rhizobiaceae;Ensifer | 0 | 0.0% | 0.0% | 0.0% |
|  | Bacteria;Proteobacteria;Alphaproteobacteria;Rhizobiales;Rhizobiaceae;Other | 0 | 0.1% | 0.1% | 0.1% |
|  | Bacteria;Proteobacteria;Alphaproteobacteria;Rhizobiales;Xanthobacteraceae;Other | 0 | 0.0% | 0.0% | 0.0% |
|  | Bacteria;Proteobacteria;Alphaproteobacteria;Rhodospirillales;Acetobacteraceae;Other | 0 | 0.1% | 0.1% | 0.1% |
|  | Bacteria;Proteobacteria;Alphaproteobacteria;Rhodospirillales;Other;Other | 0 | 0.1% | 0.1% | 0.1% |
|  | Bacteria;Proteobacteria;Alphaproteobacteria;Rickettsiales;Rickettsiaceae;Rickettsia | 0 | 0.0% | 0.0% | 0.0% |
|  | Bacteria;Proteobacteria;Alphaproteobacteria;Sphingomonadales;Erythrobacteraceae;Other | 0 | 0.0% | 0.0% | 0.0% |
|  | Bacteria;Proteobacteria;Alphaproteobacteria;Sphingomonadales;Erythrobacteraceae;Porphyrobacter | 0 | 0.0% | 0.0% | 0.0% |
|  | Bacteria;Proteobacteria;Alphaproteobacteria;Sphingomonadales;Other;Other | 0 | 0.2% | 0.2% | 0.2% |
|  | Bacteria;Proteobacteria;Alphaproteobacteria;Sphingomonadales;Sphingomonadaceae;Other | 0 | 0.0% | 0.0% | 0.0% |
|  | Bacteria;Proteobacteria;Alphaproteobacteria;Sphingomonadales;Sphingomonadaceae;Sandaracinobacter | 0 | 0.0% | 0.0% | 0.0% |
|  | Bacteria;Proteobacteria;Alphaproteobacteria;Sphingomonadales;Sphingomonadaceae;Sphingobium | 0 | 0.0% | 0.0% | 0.0% |
|  | Bacteria;Proteobacteria;Alphaproteobacteria;Sphingomonadales;Sphingomonadaceae;Sphingomonas | 0 | 0.0% | 0.0% | 0.0% |
|  | Bacteria;Proteobacteria;Betaproteobacteria;Burkholderiales;Alcaligenaceae;Castellaniella | 0 | 0.0% | 0.0% | 0.0% |
|  | Bacteria;Proteobacteria;Betaproteobacteria;Burkholderiales;Alcaligenaceae;Other | 0 | 0.0% | 0.0% | 0.0% |
|  | Bacteria;Proteobacteria;Betaproteobacteria;Burkholderiales;Burkholderiaceae;Burkholderia | 0 | 0.3% | 0.3% | 0.3% |
|  | Bacteria;Proteobacteria;Betaproteobacteria;Burkholderiales;Burkholderiaceae;Chitinimonas | 0 | 0.0% | 0.0% | 0.0% |
|  | Bacteria;Proteobacteria;Betaproteobacteria;Burkholderiales;Burkholderiaceae;Cupriavidus | 0 | 0.0% | 0.0% | 0.0% |
|  | Bacteria;Proteobacteria;Betaproteobacteria;Burkholderiales;Burkholderiaceae;Other | 0 | 0.0% | 0.0% | 0.0% |
|  | Bacteria;Proteobacteria;Betaproteobacteria;Burkholderiales;Comamonadaceae;Other | 0 | 0.1% | 0.1% | 0.0% |
|  | Bacteria;Proteobacteria;Betaproteobacteria;Burkholderiales;Other;Other | 0 | 1.1% | 1.1% | 1.1% |
|  | Bacteria;Proteobacteria;Betaproteobacteria;Burkholderiales;Oxalobacteraceae;Herbaspirillum | 0 | 0.0% | 0.0% | 0.0% |
|  | Bacteria;Proteobacteria;Betaproteobacteria;Burkholderiales;Oxalobacteraceae;Herminiimonas | 0 | 0.1% | 0.1% | 0.0% |
|  | Bacteria;Proteobacteria;Betaproteobacteria;Burkholderiales;Oxalobacteraceae;Massilia | 0 | 0.2% | 0.2% | 0.2% |
|  | Bacteria;Proteobacteria;Betaproteobacteria;Burkholderiales;Oxalobacteraceae;Other | 0 | 0.1% | 0.1% | 0.1% |
|  | Bacteria;Proteobacteria;Betaproteobacteria;Neisseriales;Neisseriaceae;Other | 0 | 0.0% | 0.0% | 0.0% |
|  | Bacteria;Proteobacteria;Betaproteobacteria;Nitrosomonadales;Nitrosomonadaceae;Nitrosospira | 0 | 0.0% | 0.0% | 0.0% |
|  | Bacteria;Proteobacteria;Betaproteobacteria;Other;Other;Other | 0 | 8.1% | 8.1% | 8.2% |
|  | Bacteria;Proteobacteria;Betaproteobacteria;Rhodocyclales;Rhodocyclaceae;Other | 0 | 0.0% | 0.0% | 0.0% |
|  | Bacteria;Proteobacteria;Deltaproteobacteria;Bdellovibrionales;Bacteriovoracaceae;Peredibacter | 0 | 0.0% | 0.0% | 0.0% |
|  | Bacteria;Proteobacteria;Deltaproteobacteria;Desulfuromonadales;Geobacteraceae;Geobacter | 0 | 0.3% | 0.3% | 0.3% |
|  | Bacteria;Proteobacteria;Deltaproteobacteria;Desulfuromonadales;Other;Other | 0 | 0.0% | 0.0% | 0.0% |
|  | Bacteria;Proteobacteria;Deltaproteobacteria;Myxococcales;Cystobacteraceae;Anaeromyxobacter | 0 | 0.0% | 0.0% | 0.0% |
|  | Bacteria;Proteobacteria;Deltaproteobacteria;Myxococcales;Cystobacteraceae;Other | 0 | 0.4% | 0.3% | 0.4% |
|  | Bacteria;Proteobacteria;Deltaproteobacteria;Myxococcales;Cystobacteraceae;Stigmatella | 0 | 0.1% | 0.1% | 0.1% |
|  | Bacteria;Proteobacteria;Deltaproteobacteria;Myxococcales;Haliangiaceae;Haliangium | 0 | 0.6% | 0.6% | 0.6% |
|  | Bacteria;Proteobacteria;Deltaproteobacteria;Myxococcales;Nannocystaceae;Nannocystis | 0 | 0.0% | 0.0% | 0.0% |
|  | Bacteria;Proteobacteria;Deltaproteobacteria;Myxococcales;Other;Other | 0 | 3.4% | 3.4% | 3.4% |
|  | Bacteria;Proteobacteria;Deltaproteobacteria;Myxococcales;Polyangiaceae;Chondromyces | 0 | 0.1% | 0.1% | 0.1% |
|  | Bacteria;Proteobacteria;Deltaproteobacteria;Myxococcales;Polyangiaceae;Other | 0 | 0.0% | 0.0% | 0.0% |
|  | Bacteria;Proteobacteria;Deltaproteobacteria;Myxococcales;Polyangiaceae;Sorangium | 0 | 0.3% | 0.3% | 0.3% |
|  | Bacteria;Proteobacteria;Deltaproteobacteria;Other;Other;Other | 0 | 0.6% | 0.6% | 0.6% |
|  | Bacteria;Proteobacteria;Gammaproteobacteria;Enterobacteriales;Enterobacteriaceae;Other | 0 | 0.0% | 0.0% | 0.0% |
|  | Bacteria;Proteobacteria;Gammaproteobacteria;Gammaproteobacteria\_incertae\_sedis;Solimonas;Other | 0 | 0.0% | 0.0% | 0.0% |
|  | Bacteria;Proteobacteria;Gammaproteobacteria;Legionellales;Coxiellaceae;Aquicella | 0 | 0.0% | 0.0% | 0.0% |
|  | Bacteria;Proteobacteria;Gammaproteobacteria;Legionellales;Legionellaceae;Tatlockia | 0 | 0.0% | 0.0% | 0.0% |
|  | Bacteria;Proteobacteria;Gammaproteobacteria;Other;Other;Other | 0 | 1.7% | 1.7% | 1.7% |
|  | Bacteria;Proteobacteria;Gammaproteobacteria;Pseudomonadales;Pseudomonadaceae;Cellvibrio | 0 | 0.1% | 0.1% | 0.1% |
|  | Bacteria;Proteobacteria;Gammaproteobacteria;Pseudomonadales;Pseudomonadaceae;Other | 0 | 0.1% | 0.1% | 0.1% |
|  | Bacteria;Proteobacteria;Gammaproteobacteria;Pseudomonadales;Pseudomonadaceae;Pseudomonas | 0 | 0.6% | 0.6% | 0.6% |
|  | Bacteria;Proteobacteria;Gammaproteobacteria;Xanthomonadales;Other;Other | 0 | 0.0% | 0.0% | 0.0% |
|  | Bacteria;Proteobacteria;Gammaproteobacteria;Xanthomonadales;Sinobacteraceae;Nevskia | 0 | 0.0% | 0.0% | 0.0% |
|  | Bacteria;Proteobacteria;Gammaproteobacteria;Xanthomonadales;Xanthomonadaceae;Dokdonella | 0 | 0.2% | 0.2% | 0.2% |
|  | Bacteria;Proteobacteria;Gammaproteobacteria;Xanthomonadales;Xanthomonadaceae;Dyella | 0 | 0.1% | 0.1% | 0.0% |
|  | Bacteria;Proteobacteria;Gammaproteobacteria;Xanthomonadales;Xanthomonadaceae;Other | 0 | 0.2% | 0.2% | 0.2% |
|  | Bacteria;Proteobacteria;Gammaproteobacteria;Xanthomonadales;Xanthomonadaceae;Xanthomonas | 0 | 0.0% | 0.0% | 0.0% |
|  | Bacteria;Proteobacteria;Other;Other;Other;Other | 0 | 13.5% | 13.5% | 13.5% |
|  | Bacteria;Spirochaetes;Spirochaetes;Spirochaetales;Leptospiraceae;Turneriella | 0 | 0.0% | 0.0% | 0.0% |
|  | Bacteria;TM7;TM7\_genera\_incertae\_sedis;Other;Other;Other | 0 | 0.4% | 0.4% | 0.4% |
|  | Bacteria;Verrucomicrobia;Other;Other;Other;Other | 0 | 0.1% | 0.1% | 0.1% |
|  | Bacteria;Verrucomicrobia;Spartobacteria;Other;Other;Other | 0 | 0.0% | 0.0% | 0.0% |
|  | Bacteria;Verrucomicrobia;Spartobacteria;Spartobacteria\_genera\_incertae\_sedis;Other;Other | 0 | 0.0% | 0.0% | 0.0% |
|  | Bacteria;Verrucomicrobia;Subdivision3;Subdivision3\_genera\_incertae\_sedis;Other;Other | 0 | 0.9% | 0.9% | 0.9% |
|  | Bacteria;Verrucomicrobia;Subdivision5;Subdivision5\_genera\_incertae\_sedis;Other;Other | 0 | 0.0% | 0.0% | 0.0% |
|  | Unclassified;Other;Other;Other;Other;Other | 0 | 0.3% | 0.3% | 0.3% |
