# Supplementary material for: Stability of operational taxonomic units: an important but neglected property for analyzing microbial diversity
Source: Microbiome. 2015 May 20;3:20. doi: 10.1186/s40168-015-0081-x (PMC4438525; doi:10.1186/s40168-015-0081-x)
Supplement: Additional file 4: — Taxonomic composition from phylum to genus level, comparing 60% and full datasets using CL. All of the subsamples were rarefied to 30,000 sequences per sample (60% of the full dataset) to be included in this analysis. [file 40168_2015_81_MOESM4_ESM.zip › taxa_summary_plots/charts/hFil570eejJ8DnmNhaYwtifCTk1CDc_legend.pdf]

- Archaea;Euryarchaeota;Other;Other
- Archaea;Other;Other;Other
- Bacteria;Acidobacteria;Acidobacteria_Gp1;Gp1
- Bacteria;Acidobacteria;Acidobacteria_Gp2;Gp2
- Bacteria;Acidobacteria;Acidobacteria_Gp22;Gp22
- Bacteria;Acidobacteria;Acidobacteria_Gp3;Gp3
- Bacteria;Acidobacteria;Acidobacteria_Gp4;Gp4
- Bacteria;Acidobacteria;Acidobacteria_Gp5;Gp5
- Bacteria;Acidobacteria;Acidobacteria_Gp6;Gp6
- Bacteria;Acidobacteria;Acidobacteria_Gp7;Gp7
- Bacteria;Acidobacteria;Holophagae;Holophagales
- Bacteria;Acidobacteria;Other;Other
- Bacteria;Actinobacteria;Actinobacteria;Acidimicrobiales
- Bacteria;Actinobacteria;Actinobacteria;Actinomycetales
- Bacteria;Actinobacteria;Actinobacteria;Other
- Bacteria;Actinobacteria;Actinobacteria;Solirubrobacterales
- Bacteria;Bacteroidetes;Flavobacteria;Flavobacteriales
- Bacteria;Bacteroidetes;Other;Other
- Bacteria;Bacteroidetes;Sphingobacteria;Sphingobacteriales
- Bacteria;Chlamydiae;Chlamydiae;Chlamydiales
- Bacteria;Chloroflexi;Other;Other
- Bacteria;Firmicutes;Bacilli;Bacillales
- Bacteria;Firmicutes;Bacilli;Other
- Bacteria;Firmicutes;Clostridia;Clostridiales
- Bacteria;Firmicutes;Clostridia;Other
- Bacteria;Firmicutes;Other;Other
- Bacteria;Gemmatimonadetes;Gemmatimonadetes;Gemmatimonadales
- Bacteria;Nitrospira;Nitrospira;Nitrospirales
- Bacteria;OP10;OP10_genera_incertae_sedis;Other
- Bacteria;Other;Other;Other
- Bacteria;Planctomycetes;Planctomycetacia;Planctomycetales
- Bacteria;Proteobacteria;Alphaproteobacteria;Caulobacterales
- Bacteria;Proteobacteria;Alphaproteobacteria;Other
- Bacteria;Proteobacteria;Alphaproteobacteria;Rhizobiales
- Bacteria;Proteobacteria;Alphaproteobacteria;Rhodospirillales
- Bacteria;Proteobacteria;Alphaproteobacteria;Rickettsiales
- Bacteria;Proteobacteria;Alphaproteobacteria;Sphingomonadales
- Bacteria;Proteobacteria;Betaproteobacteria;Burkholderiales
- Bacteria;Proteobacteria;Betaproteobacteria;Neisseriales
- Bacteria;Proteobacteria;Betaproteobacteria;Nitrosomonadales
- Bacteria;Proteobacteria;Betaproteobacteria;Other
- Bacteria;Proteobacteria;Betaproteobacteria;Rhodocyclales
- Bacteria;Proteobacteria;Deltaproteobacteria;Bdellovibrionales
- Bacteria;Proteobacteria;Deltaproteobacteria;Desulfuromonadales
- Bacteria;Proteobacteria;Deltaproteobacteria;Myxococcales
- Bacteria;Proteobacteria;Deltaproteobacteria;Other
- Bacteria;Proteobacteria;Gammaproteobacteria;Enterobacteriales
- Bacteria;Proteobacteria;Gammaproteobacteria;Gammaproteobacteria_incertae_sedis
- Bacteria;Proteobacteria;Gammaproteobacteria;Legionellales
- Bacteria;Proteobacteria;Gammaproteobacteria;Other
- Bacteria;Proteobacteria;Gammaproteobacteria;Pseudomonadales
- Bacteria;Proteobacteria;Gammaproteobacteria;Xanthomonadales
- Bacteria;Proteobacteria;Other;Other
- Bacteria;Spirochaetes;Spirochaetes;Spirochaetales
- Bacteria;TM7;TM7_genera_incertae_sedis;Other
- Bacteria;Verrucomicrobia;Other;Other
- Bacteria;Verrucomicrobia;Spartobacteria;Other
- Bacteria;Verrucomicrobia;Spartobacteria;Spartobacteria_genera_incertae_sedis
- Bacteria;Verrucomicrobia;Subdivision3;Subdivision3_genera_incertae_sedis
- Bacteria;Verrucomicrobia;Subdivision5;Subdivision5_genera_incertae_sedis
- Unclassified;Other;Other;Other
