# Supplementary material for: Stability of operational taxonomic units: an important but neglected property for analyzing microbial diversity
Source: Microbiome. 2015 May 20;3:20. doi: 10.1186/s40168-015-0081-x (PMC4438525; doi:10.1186/s40168-015-0081-x)
Supplement: Additional file 4: — Taxonomic composition from phylum to genus level, comparing 60% and full datasets using CL. All of the subsamples were rarefied to 30,000 sequences per sample (60% of the full dataset) to be included in this analysis. [file 40168_2015_81_MOESM4_ESM.zip › taxa_summary_plots/charts/K3qZYZ0up21F32Dsgr7nHTQFP37H0g_legend.pdf]

Archaea;Euryarchaeota;Other
Archaea;Other;Other
Bacteria;Acidobacteria;Acidobacteria_Gp1
Bacteria;Acidobacteria;Acidobacteria_Gp2
Bacteria;Acidobacteria;Acidobacteria_Gp22
Bacteria;Acidobacteria;Acidobacteria_Gp3
Bacteria;Acidobacteria;Acidobacteria_Gp4
Bacteria;Acidobacteria;Acidobacteria_Gp5
Bacteria;Acidobacteria;Acidobacteria_Gp6
Bacteria;Acidobacteria;Acidobacteria_Gp7
Bacteria;Acidobacteria;Holophagae
Bacteria;Acidobacteria;Other
Bacteria;Actinobacteria;Actinobacteria
Bacteria;Bacteroidetes;Flavobacteria
Bacteria;Bacteroidetes;Other
Bacteria;Bacteroidetes;Sphingobacteria
Bacteria;Chlamydiae;Chlamydiae
Bacteria;Chloroflexi;Other
Bacteria;Firmicutes;Bacilli
Bacteria;Firmicutes;Clostridia
Bacteria;Firmicutes;Other
Bacteria;Gemmatimonadetes;Gemmatimonadetes
Bacteria;Nitrospira;Nitrospira
Bacteria;OP10;OP10_genera_incertae_sedis
Bacteria;Other;Other
Bacteria;Planctomycetes;Planctomycetacia
Bacteria;Proteobacteria;Alphaproteobacteria
Bacteria;Proteobacteria;Betaproteobacteria
Bacteria;Proteobacteria;Deltaproteobacteria
Bacteria;Proteobacteria;Gammaproteobacteria
Bacteria;Proteobacteria;Other
Bacteria;Spirochaetes;Spirochaetes
Bacteria;TM7;TM7_genera_incertae_sedis
Bacteria;Verrucomicrobia;Other
Bacteria;Verrucomicrobia;Spartobacteria
Bacteria;Verrucomicrobia;Subdivision3
Bacteria;Verrucomicrobia;Subdivision5
Unclassified;Other;Other
