# Supplementary material for: Stability of operational taxonomic units: an important but neglected property for analyzing microbial diversity
Source: Microbiome. 2015 May 20;3:20. doi: 10.1186/s40168-015-0081-x (PMC4438525; doi:10.1186/s40168-015-0081-x)
Supplement: Additional file 4: — Taxonomic composition from phylum to genus level, comparing 60% and full datasets using CL. All of the subsamples were rarefied to 30,000 sequences per sample (60% of the full dataset) to be included in this analysis. [file 40168_2015_81_MOESM4_ESM.zip › taxa_summary_plots/charts/Liij9PGrAWTg0zzZJksuYGI0OszmZ8_legend.pdf]

Archaea;Euryarchaeota;Other;Other;Other
Archaea;Other;Other;Other;Other
Bacteria;Acidobacteria;Acidobacteria_Gp1;Gp1;Other
Bacteria;Acidobacteria;Acidobacteria_Gp2;Gp2;Other
Bacteria;Acidobacteria;Acidobacteria_Gp22;Gp22;Other
Bacteria;Acidobacteria;Acidobacteria_Gp3;Gp3;Other
Bacteria;Acidobacteria;Acidobacteria_Gp4;Gp4;Other
Bacteria;Acidobacteria;Acidobacteria_Gp5;Gp5;Other
Bacteria;Acidobacteria;Acidobacteria_Gp6;Gp6;Other
Bacteria;Acidobacteria;Acidobacteria_Gp7;Gp7;Other
Bacteria;Acidobacteria;Holophagae;Holophagales;Holophagaceae
Bacteria;Acidobacteria;Other;Other;Other
Bacteria;Actinobacteria;Actinobacteria;Acidimicrobiales;Iamiaceae
Bacteria;Actinobacteria;Actinobacteria;Acidimicrobiales;Other
Bacteria;Actinobacteria;Actinobacteria;Actinomycetales;Geodermatophilaceae
Bacteria;Actinobacteria;Actinobacteria;Actinomycetales;Microbacteriaceae
Bacteria;Actinobacteria;Actinobacteria;Actinomycetales;Micrococcaceae
Bacteria;Actinobacteria;Actinobacteria;Actinomycetales;Micromonosporaceae
Bacteria;Actinobacteria;Actinobacteria;Actinomycetales;Nocardiaceae
Bacteria;Actinobacteria;Actinobacteria;Actinomycetales;Nocardioidaceae
Bacteria;Actinobacteria;Actinobacteria;Actinomycetales;Other
Bacteria;Actinobacteria;Actinobacteria;Actinomycetales;Pseudonocardiaceae
Bacteria;Actinobacteria;Actinobacteria;Actinomycetales;Streptomycetaceae
Bacteria;Actinobacteria;Actinobacteria;Actinomycetales;Streptosporangiaceae
Bacteria;Actinobacteria;Actinobacteria;Actinomycetales;Thermomonosporaceae
Bacteria;Actinobacteria;Actinobacteria;Other;Other
Bacteria;Actinobacteria;Actinobacteria;Solirubrobacterales;Other
Bacteria;Actinobacteria;Actinobacteria;Solirubrobacterales;Patulibacteraceae
Bacteria;Actinobacteria;Actinobacteria;Solirubrobacterales;Solirubrobacteraceae
Bacteria;Bacteroidetes;Flavobacteria;Flavobacteriales;Cryomorphaceae
Bacteria;Bacteroidetes;Flavobacteria;Flavobacteriales;Flavobacteriaceae
Bacteria;Bacteroidetes;Flavobacteria;Flavobacteriales;Other
Bacteria;Bacteroidetes;Other;Other;Other
Bacteria;Bacteroidetes;Sphingobacteria;Sphingobacteriales;Chitinophagaceae
Bacteria;Bacteroidetes;Sphingobacteria;Sphingobacteriales;Cytophagaceae
Bacteria;Bacteroidetes;Sphingobacteria;Sphingobacteriales;Other
Bacteria;Bacteroidetes;Sphingobacteria;Sphingobacteriales;Sphingobacteriaceae
Bacteria;Chlamydiae;Chlamydiae;Chlamydiales;Other
Bacteria;Chlamydiae;Chlamydiae;Chlamydiales;Parachlamydiaceae
Bacteria;Chloroflexi;Other;Other;Other
Bacteria;Firmicutes;Bacilli;Bacillales;Bacillaceae
Bacteria;Firmicutes;Bacilli;Bacillales;Other
Bacteria;Firmicutes;Bacilli;Bacillales;Paenibacillaceae
Bacteria;Firmicutes;Bacilli;Bacillales;Thermoactinomycetaceae
Bacteria;Firmicutes;Bacilli;Other;Other
Bacteria;Firmicutes;Clostridia;Clostridiales;Clostridiaceae
Bacteria;Firmicutes;Clostridia;Other;Other
Bacteria;Firmicutes;Other;Other;Other
Bacteria;Gemmatimonadetes;Gemmatimonadetes;Gemmatimonadales;Gemmatimonadaceae
Bacteria;Nitrospira;Nitrospira;Nitrospirales;Nitrospiraceae
Bacteria;OP10;OP10_genera_incertae_sedis;Other;Other
Bacteria;Other;Other;Other;Other
Bacteria;Planctomycetes;Planctomycetacia;Planctomycetales;Planctomycetaceae
Bacteria;Proteobacteria;Alphaproteobacteria;Caulobacterales;Caulobacteraceae
Bacteria;Proteobacteria;Alphaproteobacteria;Other;Other
Bacteria;Proteobacteria;Alphaproteobacteria;Rhizobiales;Bradyrhizobiaceae
Bacteria;Proteobacteria;Alphaproteobacteria;Rhizobiales;Brucellaceae
Bacteria;Proteobacteria;Alphaproteobacteria;Rhizobiales;Hyphomicrobiaceae
Bacteria;Proteobacteria;Alphaproteobacteria;Rhizobiales;Methylobacteriaceae
Bacteria;Proteobacteria;Alphaproteobacteria;Rhizobiales;Methylocystaceae
Bacteria;Proteobacteria;Alphaproteobacteria;Rhizobiales;Other
Bacteria;Proteobacteria;Alphaproteobacteria;Rhizobiales;Rhizobiaceae
Bacteria;Proteobacteria;Alphaproteobacteria;Rhizobiales;Xanthobacteraceae
Bacteria;Proteobacteria;Alphaproteobacteria;Rhodospirillales;Acetobacteraceae
Bacteria;Proteobacteria;Alphaproteobacteria;Rhodospirillales;Other
Bacteria;Proteobacteria;Alphaproteobacteria;Rickettsiales;Rickettsiaceae
Bacteria;Proteobacteria;Alphaproteobacteria;Sphingomonadales;Erythrobacteraceae
Bacteria;Proteobacteria;Alphaproteobacteria;Sphingomonadales;Other
Bacteria;Proteobacteria;Alphaproteobacteria;Sphingomonadales;Sphingomonadaceae
Bacteria;Proteobacteria;Betaproteobacteria;Burkholderiales;Alcaligenaceae
Bacteria;Proteobacteria;Betaproteobacteria;Burkholderiales;Burkholderiaceae
Bacteria;Proteobacteria;Betaproteobacteria;Burkholderiales;Comamonadaceae
Bacteria;Proteobacteria;Betaproteobacteria;Burkholderiales;Other
Bacteria;Proteobacteria;Betaproteobacteria;Burkholderiales;Oxalobacteraceae
Bacteria;Proteobacteria;Betaproteobacteria;Neisseriales;Neisseriaceae
Bacteria;Proteobacteria;Betaproteobacteria;Nitrosomonadales;Nitrosomonadaceae
Bacteria;Proteobacteria;Betaproteobacteria;Other;Other
Bacteria;Proteobacteria;Betaproteobacteria;Rhodocyclales;Rhodocyclaceae
Bacteria;Proteobacteria;Deltaproteobacteria;Bdellovibrionales;Bacteriovoracaceae
Bacteria;Proteobacteria;Deltaproteobacteria;Desulfuromonadales;Geobacteraceae
Bacteria;Proteobacteria;Deltaproteobacteria;Desulfuromonadales;Other
Bacteria;Proteobacteria;Deltaproteobacteria;Myxococcales;Cystobacteraceae
Bacteria;Proteobacteria;Deltaproteobacteria;Myxococcales;Haliangiaceae
Bacteria;Proteobacteria;Deltaproteobacteria;Myxococcales;Nannocystaceae
Bacteria;Proteobacteria;Deltaproteobacteria;Myxococcales;Other
Bacteria;Proteobacteria;Deltaproteobacteria;Myxococcales;Polyangiaceae
Bacteria;Proteobacteria;Deltaproteobacteria;Other;Other
Bacteria;Proteobacteria;Gammaproteobacteria;Enterobacteriales;Enterobacteriaceae
Bacteria;Proteobacteria;Gammaproteobacteria;Gammaproteobacteria_incertae_sedis;Solimonas
Bacteria;Proteobacteria;Gammaproteobacteria;Legionellales;Coxiellaceae
Bacteria;Proteobacteria;Gammaproteobacteria;Legionellales;Legionellaceae
Bacteria;Proteobacteria;Gammaproteobacteria;Other;Other
Bacteria;Proteobacteria;Gammaproteobacteria;Pseudomonadales;Pseudomonadaceae
Bacteria;Proteobacteria;Gammaproteobacteria;Xanthomonadales;Other
Bacteria;Proteobacteria;Gammaproteobacteria;Xanthomonadales;Sinobacteraceae
Bacteria;Proteobacteria;Gammaproteobacteria;Xanthomonadales;Xanthomonadaceae
Bacteria;Proteobacteria;Other;Other;Other
Bacteria;Spirochaetes;Spirochaetes;Spirochaetales;Leptospiraceae
Bacteria;TM7;TM7_genera_incertae_sedis;Other;Other
Bacteria;Verrucomicrobia;Other;Other;Other
Bacteria;Verrucomicrobia;Spartobacteria;Other;Other
Bacteria;Verrucomicrobia;Spartobacteria;Spartobacteria_genera_incertae_sedis;Other
Bacteria;Verrucomicrobia;Subdivision3;Subdivision3_genera_incertae_sedis;Other
Bacteria;Verrucomicrobia;Subdivision5;Subdivision5_genera_incertae_sedis;Other
Unclassified;Other;Other;Other;Other
