# Supplementary material for: Stability of operational taxonomic units: an important but neglected property for analyzing microbial diversity
Source: Microbiome. 2015 May 20;3:20. doi: 10.1186/s40168-015-0081-x (PMC4438525; doi:10.1186/s40168-015-0081-x)
Supplement: Additional file 4: — Taxonomic composition from phylum to genus level, comparing 60% and full datasets using CL. All of the subsamples were rarefied to 30,000 sequences per sample (60% of the full dataset) to be included in this analysis. [file 40168_2015_81_MOESM4_ESM.zip › taxa_summary_plots/charts/nzI3lUHt8zstgmO0xSpzzfZZ2Gm1NW_legend.pdf]

Archaea;Euryarchaeota;Other;Other;Other;Other
Archaea;Other;Other;Other;Other;Other
Bacteria;Acidobacteria;Acidobacteria_Gp1;Gp1;Other;Other
Bacteria;Acidobacteria;Acidobacteria_Gp2;Gp2;Other;Other
Bacteria;Acidobacteria;Acidobacteria_Gp22;Gp22;Other;Other
Bacteria;Acidobacteria;Acidobacteria_Gp3;Gp3;Other;Other
Bacteria;Acidobacteria;Acidobacteria_Gp4;Gp4;Other;Other
Bacteria;Acidobacteria;Acidobacteria_Gp5;Gp5;Other;Other
Bacteria;Acidobacteria;Acidobacteria_Gp6;Gp6;Other;Other
Bacteria;Acidobacteria;Acidobacteria_Gp7;Gp7;Other;Other
Bacteria;Acidobacteria;Holophagae;Holophagales;Holophagaceae;Other
Bacteria;Acidobacteria;Other;Other;Other;Other
Bacteria;Actinobacteria;Actinobacteria;Acidimicrobiales;Iamiaceae;Iamia
Bacteria;Actinobacteria;Actinobacteria;Acidimicrobiales;Other;Other
Bacteria;Actinobacteria;Actinobacteria;Actinomycetales;Geodermatophilaceae;Other
Bacteria;Actinobacteria;Actinobacteria;Actinomycetales;Microbacteriaceae;Other
Bacteria;Actinobacteria;Actinobacteria;Actinomycetales;Micrococcaceae;Other
Bacteria;Actinobacteria;Actinobacteria;Actinomycetales;Micromonosporaceae;Actinoplanes
Bacteria;Actinobacteria;Actinobacteria;Actinomycetales;Micromonosporaceae;Other
Bacteria;Actinobacteria;Actinobacteria;Actinomycetales;Nocardiaceae;Other
Bacteria;Actinobacteria;Actinobacteria;Actinomycetales;Nocardioidaceae;Aeromicrobium
Bacteria;Actinobacteria;Actinobacteria;Actinomycetales;Nocardioidaceae;Kribbella
Bacteria;Actinobacteria;Actinobacteria;Actinomycetales;Nocardioidaceae;Other
Bacteria;Actinobacteria;Actinobacteria;Actinomycetales;Other;Other
Bacteria;Actinobacteria;Actinobacteria;Actinomycetales;Pseudonocardiaceae;Amycolatopsis
Bacteria;Actinobacteria;Actinobacteria;Actinomycetales;Pseudonocardiaceae;Other
Bacteria;Actinobacteria;Actinobacteria;Actinomycetales;Pseudonocardiaceae;Pseudonocardia
Bacteria;Actinobacteria;Actinobacteria;Actinomycetales;Streptomycetaceae;Kitasatospora
Bacteria;Actinobacteria;Actinobacteria;Actinomycetales;Streptomycetaceae;Other
Bacteria;Actinobacteria;Actinobacteria;Actinomycetales;Streptomycetaceae;Streptacidiphilus
Bacteria;Actinobacteria;Actinobacteria;Actinomycetales;Streptomycetaceae;Streptomyces
Bacteria;Actinobacteria;Actinobacteria;Actinomycetales;Streptosporangiaceae;Other
Bacteria;Actinobacteria;Actinobacteria;Actinomycetales;Thermomonosporaceae;Actinocorallia
Bacteria;Actinobacteria;Actinobacteria;Actinomycetales;Thermomonosporaceae;Other
Bacteria;Actinobacteria;Actinobacteria;Other;Other;Other
Bacteria;Actinobacteria;Actinobacteria;Solirubrobacterales;Other;Other
Bacteria;Actinobacteria;Actinobacteria;Solirubrobacterales;Patulibacteraceae;Patulibacter
Bacteria;Actinobacteria;Actinobacteria;Solirubrobacterales;Solirubrobacteraceae;Solirubrobacter
Bacteria;Bacteroidetes;Flavobacteria;Flavobacteriales;Cryomorphaceae;Other
Bacteria;Bacteroidetes;Flavobacteria;Flavobacteriales;Flavobacteriaceae;Flavobacterium
Bacteria;Bacteroidetes;Flavobacteria;Flavobacteriales;Flavobacteriaceae;Other
Bacteria;Bacteroidetes;Flavobacteria;Flavobacteriales;Other;Other
Bacteria;Bacteroidetes;Other;Other;Other;Other
Bacteria;Bacteroidetes;Sphingobacteria;Sphingobacteriales;Chitinophagaceae;Chitinophaga
Bacteria;Bacteroidetes;Sphingobacteria;Sphingobacteriales;Chitinophagaceae;Ferruginibacter
Bacteria;Bacteroidetes;Sphingobacteria;Sphingobacteriales;Chitinophagaceae;Filimonas
Bacteria;Bacteroidetes;Sphingobacteria;Sphingobacteriales;Chitinophagaceae;Flavisolibacter
Bacteria;Bacteroidetes;Sphingobacteria;Sphingobacteriales;Chitinophagaceae;Niastella
Bacteria;Bacteroidetes;Sphingobacteria;Sphingobacteriales;Chitinophagaceae;Other
Bacteria;Bacteroidetes;Sphingobacteria;Sphingobacteriales;Cytophagaceae;Cytophaga
Bacteria;Bacteroidetes;Sphingobacteria;Sphingobacteriales;Cytophagaceae;Dyadobacter
Bacteria;Bacteroidetes;Sphingobacteria;Sphingobacteriales;Cytophagaceae;Sporocytophaga
Bacteria;Bacteroidetes;Sphingobacteria;Sphingobacteriales;Other;Other
Bacteria;Bacteroidetes;Sphingobacteria;Sphingobacteriales;Sphingobacteriaceae;Mucilaginibacter
Bacteria;Bacteroidetes;Sphingobacteria;Sphingobacteriales;Sphingobacteriaceae;Other
Bacteria;Bacteroidetes;Sphingobacteria;Sphingobacteriales;Sphingobacteriaceae;Pedobacter
Bacteria;Chlamydiae;Chlamydiae;Chlamydiales;Other;Other
Bacteria;Chlamydiae;Chlamydiae;Chlamydiales;Parachlamydiaceae;Neochlamydia
Bacteria;Chlamydiae;Chlamydiae;Chlamydiales;Parachlamydiaceae;Other
Bacteria;Chlamydiae;Chlamydiae;Chlamydiales;Parachlamydiaceae;Parachlamydia
Bacteria;Chloroflexi;Other;Other;Other;Other
Bacteria;Firmicutes;Bacilli;Bacillales;Bacillaceae;Other
Bacteria;Firmicutes;Bacilli;Bacillales;Other;Other
Bacteria;Firmicutes;Bacilli;Bacillales;Paenibacillaceae;Other
Bacteria;Firmicutes;Bacilli;Bacillales;Paenibacillaceae;Paenibacillus
Bacteria;Firmicutes;Bacilli;Bacillales;Thermoactinomycetaceae;Other
Bacteria;Firmicutes;Bacilli;Other;Other;Other
Bacteria;Firmicutes;Clostridia;Clostridiales;Clostridiaceae;Clostridium
Bacteria;Firmicutes;Clostridia;Other;Other;Other
Bacteria;Firmicutes;Other;Other;Other;Other
Bacteria;Gemmatimonadetes;Gemmatimonadetes;Gemmatimonadales;Gemmatimonadaceae;Gemmatimonas
Bacteria;Nitrospira;Nitrospira;Nitrospirales;Nitrospiraceae;Nitrospira
Bacteria;OP10;OP10_genera_incertae_sedis;Other;Other;Other
Bacteria;Other;Other;Other;Other;Other
Bacteria;Planctomycetes;Planctomycetacia;Planctomycetales;Planctomycetaceae;Other
Bacteria;Proteobacteria;Alphaproteobacteria;Caulobacterales;Caulobacteraceae;Phenylobacterium
Bacteria;Proteobacteria;Alphaproteobacteria;Other;Other;Other
Bacteria;Proteobacteria;Alphaproteobacteria;Rhizobiales;Bradyrhizobiaceae;Bradyrhizobium
Bacteria;Proteobacteria;Alphaproteobacteria;Rhizobiales;Bradyrhizobiaceae;Nitrobacter
Bacteria;Proteobacteria;Alphaproteobacteria;Rhizobiales;Bradyrhizobiaceae;Other
Bacteria;Proteobacteria;Alphaproteobacteria;Rhizobiales;Brucellaceae;Brucella
Bacteria;Proteobacteria;Alphaproteobacteria;Rhizobiales;Brucellaceae;Other
Bacteria;Proteobacteria;Alphaproteobacteria;Rhizobiales;Hyphomicrobiaceae;Devosia
Bacteria;Proteobacteria;Alphaproteobacteria;Rhizobiales;Hyphomicrobiaceae;Hyphomicrobium
Bacteria;Proteobacteria;Alphaproteobacteria;Rhizobiales;Methylobacteriaceae;Methylobacterium
Bacteria;Proteobacteria;Alphaproteobacteria;Rhizobiales;Methylocystaceae;Other
Bacteria;Proteobacteria;Alphaproteobacteria;Rhizobiales;Other;Other
Bacteria;Proteobacteria;Alphaproteobacteria;Rhizobiales;Rhizobiaceae;Ensifer
Bacteria;Proteobacteria;Alphaproteobacteria;Rhizobiales;Rhizobiaceae;Other
Bacteria;Proteobacteria;Alphaproteobacteria;Rhizobiales;Xanthobacteraceae;Other
Bacteria;Proteobacteria;Alphaproteobacteria;Rhodospirillales;Acetobacteraceae;Other
Bacteria;Proteobacteria;Alphaproteobacteria;Rhodospirillales;Other;Other
Bacteria;Proteobacteria;Alphaproteobacteria;Rickettsiales;Rickettsiaceae;Rickettsia
Bacteria;Proteobacteria;Alphaproteobacteria;Sphingomonadales;Erythrobacteraceae;Other
Bacteria;Proteobacteria;Alphaproteobacteria;Sphingomonadales;Erythrobacteraceae;Porphyrobacter
Bacteria;Proteobacteria;Alphaproteobacteria;Sphingomonadales;Other;Other
Bacteria;Proteobacteria;Alphaproteobacteria;Sphingomonadales;Sphingomonadaceae;Other
Bacteria;Proteobacteria;Alphaproteobacteria;Sphingomonadales;Sphingomonadaceae;Sandaracinobacter
Bacteria;Proteobacteria;Alphaproteobacteria;Sphingomonadales;Sphingomonadaceae;Sphingobium
Bacteria;Proteobacteria;Alphaproteobacteria;Sphingomonadales;Sphingomonadaceae;Sphingomonas
Bacteria;Proteobacteria;Betaproteobacteria;Burkholderiales;Alcaligenaceae;Castellaniella
Bacteria;Proteobacteria;Betaproteobacteria;Burkholderiales;Alcaligenaceae;Other
Bacteria;Proteobacteria;Betaproteobacteria;Burkholderiales;Burkholderiaceae;Burkholderia
Bacteria;Proteobacteria;Betaproteobacteria;Burkholderiales;Burkholderiaceae;Chitinimonas
Bacteria;Proteobacteria;Betaproteobacteria;Burkholderiales;Burkholderiaceae;Cupriavidus
Bacteria;Proteobacteria;Betaproteobacteria;Burkholderiales;Burkholderiaceae;Other
Bacteria;Proteobacteria;Betaproteobacteria;Burkholderiales;Comamonadaceae;Other
Bacteria;Proteobacteria;Betaproteobacteria;Burkholderiales;Other;Other
Bacteria;Proteobacteria;Betaproteobacteria;Burkholderiales;Oxalobacteraceae;Herbaspirillum
Bacteria;Proteobacteria;Betaproteobacteria;Burkholderiales;Oxalobacteraceae;Herminiimonas
Bacteria;Proteobacteria;Betaproteobacteria;Burkholderiales;Oxalobacteraceae;Massilia
Bacteria;Proteobacteria;Betaproteobacteria;Burkholderiales;Oxalobacteraceae;Other
Bacteria;Proteobacteria;Betaproteobacteria;Neisseriales;Neisseriaceae;Other
Bacteria;Proteobacteria;Betaproteobacteria;Nitrosomonadales;Nitrosomonadaceae;Nitrosospira
Bacteria;Proteobacteria;Betaproteobacteria;Other;Other;Other
Bacteria;Proteobacteria;Betaproteobacteria;Rhodocyclales;Rhodocyclaceae;Other
Bacteria;Proteobacteria;Deltaproteobacteria;Bdellovibrionales;Bacteriovoracaceae;Peredibacter
Bacteria;Proteobacteria;Deltaproteobacteria;Desulfuromonadales;Geobacteraceae;Geobacter
Bacteria;Proteobacteria;Deltaproteobacteria;Desulfuromonadales;Other;Other
Bacteria;Proteobacteria;Deltaproteobacteria;Myxococcales;Cystobacteraceae;Anaeromyxobacter
Bacteria;Proteobacteria;Deltaproteobacteria;Myxococcales;Cystobacteraceae;Other
Bacteria;Proteobacteria;Deltaproteobacteria;Myxococcales;Cystobacteraceae;Stigmatella
Bacteria;Proteobacteria;Deltaproteobacteria;Myxococcales;Haliangiaceae;Haliangium
Bacteria;Proteobacteria;Deltaproteobacteria;Myxococcales;Nannocystaceae;Nannocystis
Bacteria;Proteobacteria;Deltaproteobacteria;Myxococcales;Other;Other
Bacteria;Proteobacteria;Deltaproteobacteria;Myxococcales;Polyangiaceae;Chondromyces
Bacteria;Proteobacteria;Deltaproteobacteria;Myxococcales;Polyangiaceae;Other
Bacteria;Proteobacteria;Deltaproteobacteria;Myxococcales;Polyangiaceae;Sorangium
Bacteria;Proteobacteria;Deltaproteobacteria;Other;Other;Other
Bacteria;Proteobacteria;Gammaproteobacteria;Enterobacteriales;Enterobacteriaceae;Other
Bacteria;Proteobacteria;Gammaproteobacteria;Gammaproteobacteria_incertae_sedis;Solimonas;Other
Bacteria;Proteobacteria;Gammaproteobacteria;Legionellales;Coxiellaceae;Aquicella
Bacteria;Proteobacteria;Gammaproteobacteria;Legionellales;Legionellaceae;Tatlockia
Bacteria;Proteobacteria;Gammaproteobacteria;Other;Other;Other
Bacteria;Proteobacteria;Gammaproteobacteria;Pseudomonadales;Pseudomonadaceae;Cellvibrio
Bacteria;Proteobacteria;Gammaproteobacteria;Pseudomonadales;Pseudomonadaceae;Other
Bacteria;Proteobacteria;Gammaproteobacteria;Pseudomonadales;Pseudomonadaceae;Pseudomonas
Bacteria;Proteobacteria;Gammaproteobacteria;Xanthomonadales;Other;Other
Bacteria;Proteobacteria;Gammaproteobacteria;Xanthomonadales;Sinobacteraceae;Nevskia
Bacteria;Proteobacteria;Gammaproteobacteria;Xanthomonadales;Xanthomonadaceae;Dokdonella
Bacteria;Proteobacteria;Gammaproteobacteria;Xanthomonadales;Xanthomonadaceae;Dyella
Bacteria;Proteobacteria;Gammaproteobacteria;Xanthomonadales;Xanthomonadaceae;Other
Bacteria;Proteobacteria;Gammaproteobacteria;Xanthomonadales;Xanthomonadaceae;Xanthomonas
Bacteria;Proteobacteria;Other;Other;Other;Other
Bacteria;Spirochaetes;Spirochaetes;Spirochaetales;Leptospiraceae;Turneriella
Bacteria;TM7;TM7_genera_incertae_sedis;Other;Other;Other
Bacteria;Verrucomicrobia;Other;Other;Other;Other
Bacteria;Verrucomicrobia;Spartobacteria;Other;Other;Other
Bacteria;Verrucomicrobia;Spartobacteria;Spartobacteria_genera_incertae_sedis;Other;Other
Bacteria;Verrucomicrobia;Subdivision3;Subdivision3_genera_incertae_sedis;Other;Other
Bacteria;Verrucomicrobia;Subdivision5;Subdivision5_genera_incertae_sedis;Other;Other
Unclassified;Other;Other;Other;Other;Other
