# Supplementary material for: Stability of operational taxonomic units: an important but neglected property for analyzing microbial diversity
Source: Microbiome. 2015 May 20;3:20. doi: 10.1186/s40168-015-0081-x (PMC4438525; doi:10.1186/s40168-015-0081-x)
Supplement: Additional file 6: Tables S1 and S2 — Table 1. Multiple comparisons of unstable sequences between different clustering methods after the Kruskal-Wallis test. Table 2. Multiple comparisons of MCC values between different clustering methods after the Kruskal-Wallis test. [file 40168_2015_81_MOESM6_ESM.docx]

Stable operational taxonomic units for studying microbial ecology

He *et al.*

**Table 1: Multiple comparisons of unstable sequences between different clustering methods after kruskal-wallis test.**

|  | SL | AL | DGC | AGC | Dereplication | Closed-ref | Open-ref |
| --- | --- | --- | --- | --- | --- | --- | --- |
| CL | F | T | T | T | T | T | T |
| SL |  | T | F | T | T | T | T |
| AL |  |  | F | F | T | T | T |
| DGC |  |  |  | T | T | T | T |
| AGC |  |  |  |  | T | T | F |
| Dereplication |  |  |  |  |  | F | F |
| Closed-ref |  |  |  |  |  |  | F |

**T: P<0.05**

**F: P>0.05.**

**Table 2: Multiple comparisons of MCC values between different clustering methods after kruskal-wallis test.**

|  | SL | AL | DGC | AGC | Dereplication | Closed-ref | Open-ref |
| --- | --- | --- | --- | --- | --- | --- | --- |
| CL | T | F | F | T | T | T | T |
| SL |  | T | F | F | T | T | F |
| AL |  |  | T | T | T | T | T |
| DGC |  |  |  | F | T | T | T |
| AGC |  |  |  |  | T | T | F |
| Dereplication |  |  |  |  |  | F | F |
| Closed-ref |  |  |  |  |  |  | F |

**T: P<0.05**

**F: P>0.05.**
